# Allele-specific copy-number discovery from whole-genome and whole-exome sequencing

**DOI:** 10.1093/nar/gkv319

**Published:** 2015-04-16

**Authors:** WeiBo Wang, Wei Wang, Wei Sun, James J. Crowley, Jin P. Szatkiewicz

**Affiliations:** 1 Department of Computer Science, University of North Carolina at Chapel Hill, NC 27599-3175, USA; 2 Department of Computer Science, University of California, Los Angeles, CA 90095, USA; 3 Department of Biostatistics, University of North Carolina at Chapel Hill, NC 27599-7400, USA; 4 Department of Genetics, University of North Carolina at Chapel Hill, NC 27599-7264, USA

## Abstract

Copy-number variants (CNVs) are a major form of genetic variation and a risk factor for various human diseases, so it is crucial to accurately detect and characterize them. It is conceivable that allele-specific reads from high-throughput sequencing data could be leveraged to both enhance CNV detection and produce allele-specific copy number (ASCN) calls. Although statistical methods have been developed to detect CNVs using whole-genome sequence (WGS) and/or whole-exome sequence (WES) data, information from allele-specific read counts has not yet been adequately exploited. In this paper, we develop an integrated method, called AS-GENSENG, which incorporates allele-specific read counts in CNV detection and estimates ASCN using either WGS or WES data. To evaluate the performance of AS-GENSENG, we conducted extensive simulations, generated empirical data using existing WGS and WES data sets and validated predicted CNVs using an independent methodology. We conclude that AS-GENSENG not only predicts accurate ASCN calls but also improves the accuracy of total copy number calls, owing to its unique ability to exploit information from both total and allele-specific read counts while accounting for various experimental biases in sequence data. Our novel, user-friendly and computationally efficient method and a complete analytic protocol is freely available at https://sourceforge.net/projects/asgenseng/.

## INTRODUCTION

Copy-number variants (CNVs) are a major form of genetic variation in mammals (1–4) and a risk factor for various human diseases (5–11). Indeed, CNV assessment is beginning to become a routine part of the diagnostic workup for some medical conditions, including neurobehavioral disorders (12–15). CNV assessment is also important in functional genomic studies since failing to account for copy-number differences can result in misinterpretation of data from RNA-seq, chromatin immunoprecipitation (ChIP-seq), DNase-hypersensitive site mapping (DNase-seq) or formaldehyde-assisted isolation of regulatory elements (FAIRE-seq) (16,17). For these reasons, accurate detection of CNVs is of paramount importance; and allele-specific copy number (ASCN) calls are highly desirable as it is also important to know how CNVs are allocated in diploid organisms (18,19). For example, ASCN analysis of breast tumors allowed the construction of a genome-wide map of allelic skewness in breast cancer (20). Furthermore, many recessive Mendelian disorders, such as Cohen syndrome (21), often result from the unmasking of a deleterious allele by a one copy deletion. Therefore, allele-specific CNV calls provide crucial additional information for disease studies.

Using genome-wide single-nucleotide polymorphism (SNP) arrays (22–25), allele-specific intensity signals for two SNP alleles (denoted as alleles A and B) can be obtained and integrated in CNV detection. ASCN calls can then be generated (e.g. A, AAB, BBB, ABBB). ASCN calls provide a more accurate characterization of the underlying DNA sequence of each individual, thereby reducing the rate of apparent Mendelian inconsistencies (26,27) and could improve statistical power for tests of association with complex diseases (28). Several methods have been developed for CNV detection using allele-specific probe intensities from SNP arrays (27,29,30). With Affymetrix array data, Birdsuite uses a hidden Markov model (HMM) and defines allele-specific properties of each probe through HMM emission probability (23). With Illumina array data, raw intensity data are transformed into the total intensity from both alleles (i.e. ‘log R Ratio’ or LRR) and the relative ratio of the intensity between two alleles (i.e. B Allele Frequency or BAF). HMM-based methods, such as PennCNV (29) and GenoCN (30), jointly analyze LRR and BAF in the likelihood. According to simulations and studies on individuals with known CNVs, integrating allele-specific information in array-based CNV calling not only yields ASCN but also improves the accuracy of total copy-number calls.

Recent advances in high-throughput sequencing (HTS) (31–33) are promoting whole-genome sequencing (WGS) or whole-exome sequencing (WES) as an all-in-one high-throughput assay for characterizing SNPs and CNVs. HTS-based CNV detection methods utilize a variety of signals to make calls, including read-pair, split-read or read-depth information (3,34–36). Analogous to microarray-based methods, allele-specific information could also be leveraged for HTS-based CNV detection. For cancer studies, specific methods (Patchwork (37), SomatiCA (38), WaveCNV (39), ADTEx (40)) have been developed to incorporate allele-specific information in detecting copy number aberration using tumor/normal sample pairs. Such methods typically apply a two-step approach, where read-depth ratios of the tumor/normal pairs and the minor allele frequency data are analyzed separately. However, for detecting germline CNVs, allele-specific information has not been extensively explored in the literature. With WGS data, ERDS (41) is the only existing method that leverages allele-specific information but it has a number of limitations. For example, deletions are detected by simultaneous analysis of read-depth and the total number of heterozygous SNPs followed by refinement of smaller segments (<10 kb) using read-pair information; however, duplications are detected using read-depth only. Further, ERDS estimates total copy-numbers but it is not capable of estimating ASCN. For WES data, many effective methods have been developed to estimate rare or common CNVs (42–52); however, none of the existing methods leverage allele-specific information in CNV detection or are capable of estimating ASCN. To overcome these deficiencies, here we develop a novel method that uses allele-specific information to aid the detection of both deletions and duplications and is capable of determining ASCN from both WGS and WES data.

When analyzing HTS data, it is critical to correct for various sources of experimental bias that distort the quantitative relationship between read-depth and true copy number, hindering the ability for accurate CNV detection (31,53,54). While cancer studies afford themselves the use of tumor/normal pairs, numerous techniques have been developed for germline CNV studies to normalize read-depth data (55–64). For WGS, most existing methods (56,57,60) use a two-step approach, where read-depth data from a single-genome are first adjusted to account for the effect of known sources of bias (e.g. GC content) and then the adjusted read-depth is segmented to predict CNVs. Recently we developed GENSENG (54), a one-step approach that simultaneously corrects for various sources of bias, both known and unknown, and segment read-depth data. Based on extensive evaluation, we have demonstrated that this one-step approach improves CNV detection for read-depth-based CNV detection (54). Exome sequencing introduces additional sources of noise to the raw read-depth data (42–52,65) and methods developed for WES data typically leverage the large-scale nature of exome sequencing projects for noise-reduction/data-normalization. Based on their noise-reduction techniques, most existing WES methods can be classified into two categories: either multivariate methods including principle component analysis (PCA) and singular value decomposition (SVD), or reference-set methods (43,47,48,50–52,65). The PCA/SVD methods assume that most variation observed in the sample-by-target read-depth matrix is due to noise with little contribution from CNVs and therefore remove several of the strongest variance components for the purpose of noise reduction. In this paradigm, XHMM (42) applies a PCA that is optimized for detecting rare CNVs (frequency <5%), whereas common CNVs could not fit in this model. CoNIFER (49) applies an SVD and removes the first 12–15 variance components for detecting rare CNVs but five components for common CNVs. However, as the frequencies of CNVs cannot be known before they are detected, it is challenging to determine how to choose the top-K variance components in order to prevent the PCA/SVD methods from removing true CNV signals (65). Alternatively, the reference-set methods create a baseline for each exon target from a reference group of copy-number 2, where the baseline from the reference set captures technical variation but not variation due to CNVs. Then read-depth ratios of test samples versus the baseline are computed for the purpose of noise reduction (43,47,48,50–52). However, the power to detect common CNVs is often limited, owing to the difficulty in constructing the true reference set in the presence of common CNVs, especially when the CNV frequency is high and unknown (48,50). Here we demonstrate that allele-specific read count can be leveraged to identify the proper reference group with copy-number 2 and this method subsequently improves detection of common CNVs at any frequency.

The aim of this study was to develop an integrated method, named AS-GENSENG, that can (1) detect CNVs by jointly exploiting patterns in total- and allele-specific read count, (2) estimate ASCN and (3) be applicable to both WGS and WES data. For bias correction, we inherited the one-step approach used by GENSENG (54) and leveraged allele-specific information for normalizing WES data. We evaluated AS-GENSENG using simulation and WGS or WES data from the 1000 Genomes Project (1000GP) (3,34) and compared our method to a number of state-of-the-art CNV detection algorithms in the literature (41,42,48,49,56). Furthermore, we validated a subset of CNV calls with an independent and highly accurate technology (NanoString nCounter) (66–70). In summary, we conclude that AS-GENSENG not only predicts accurate ASCN calls but also improves the accuracy of total copy number calls. For WGS data, AS-GENSENG has better overall performance in detecting CNVs than several state-of-the-arts methods for WGS data. For WES data, AS-GENSENG has better sensitivity and comparable specificity for detecting common CNVs. Our novel, user-friendly and computationally efficient method is available at https://sourceforge.net/projects/asgenseng/.

## MATERIALS AND METHODS

### Method summary

**Figure 1.**
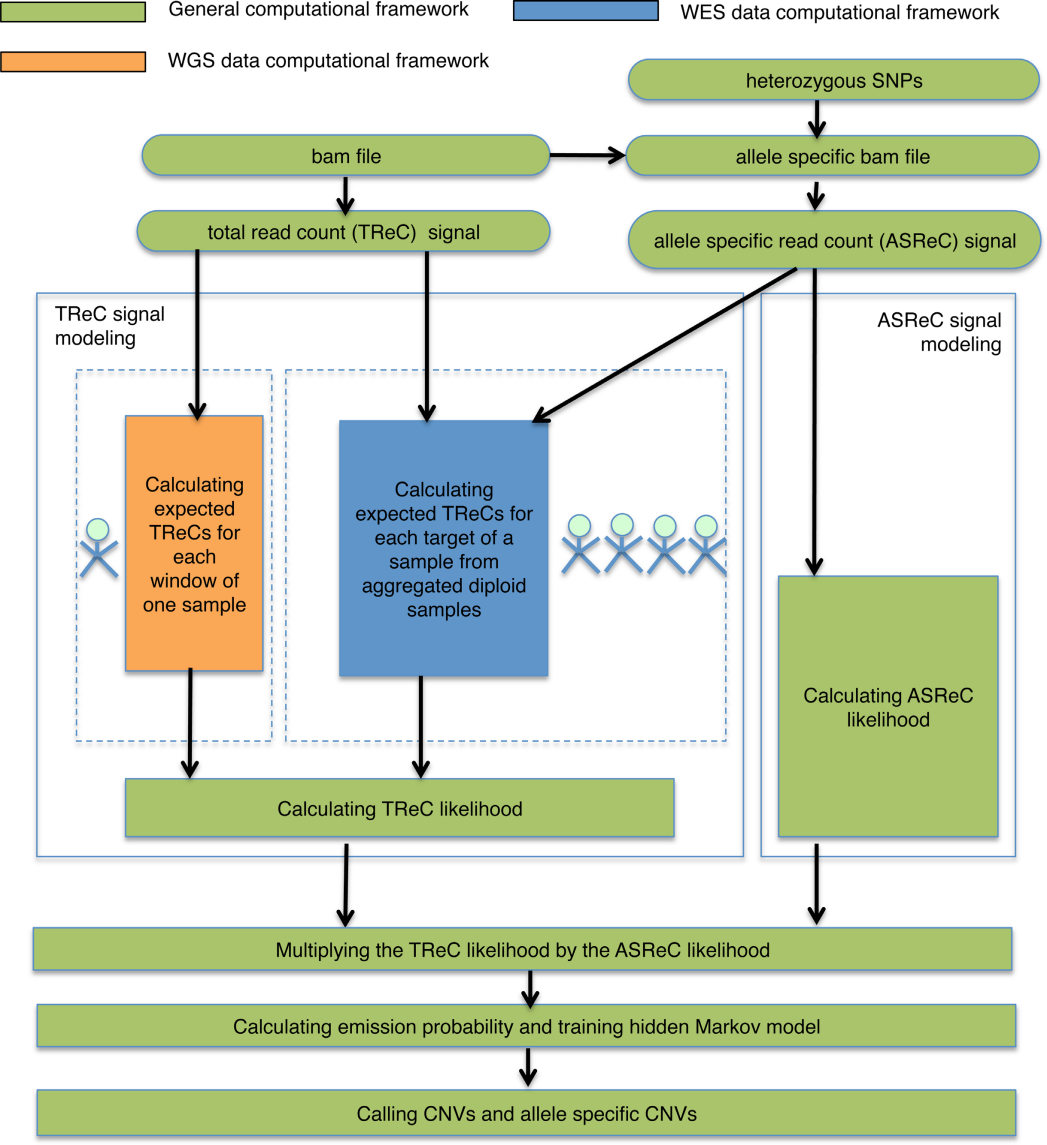
Method overview. Our method is a hidden Markov model-based algorithm. We compute the total read count (TReC) and allele-specific read count (ASReC) from the alignments (.bam file) and the allele-specific alignments at each genomic region (i.e. a window or a target). To infer the underlying copy number, we calculate the likelihood of the observed TReC and the ASReC from the estimated expected TReC for each possible underlying copy number. We calculate the likelihoods of TReC and ASReC separately. For the TReC likelihood, the calculations for whole-genome sequencing (WGS) data and whole-exome sequencing (WES) data are different. For WGS, we estimate the expected TReC using one sample; for WES, we estimate the expected TReC by aggregating multiple samples. In addition to calculating the TReC likelihood, we also utilize the ASReC likelihood in order to improve CNV-detection performance. We insert the product of the two likelihoods into the hidden Markov-model emission probability. After this training, we call CNV by identifying the change of the most likely underlying copy number as the CNV breakpoint. We call allele-specific CNV by choosing the most likely allelic configuration for each CNV.

**Figure 2.**
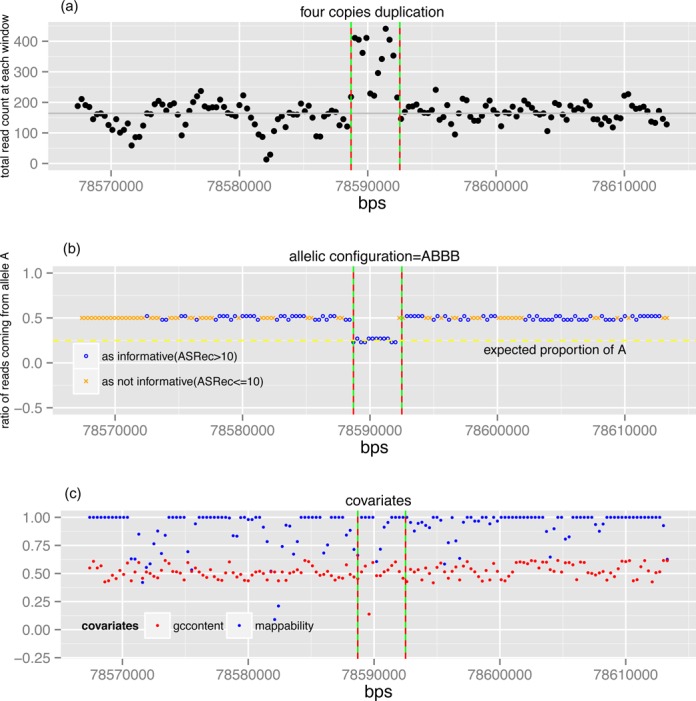
Example of CNV predicted by AS-GENSENG through joint analysis of read-count, allele-specific information and bias correction. In each panel, the X-axis indicates genomic position in chromosome one in base pairs. (**a**) Black dots on the Y-axis indicate read-count signal; red dashed lines are boundaries from ASGENSENG prediction; green solid lines are ground-truth boundaries; and gray lines are the median read-count of the chromosome. (**b**) Symbols on the Y-axis indicate the ratio of proportion of reads coming from allele A. A blue circle indicates that the number of allele-specific reads at the corresponding genomic region is >10 (i.e. AS informative); an orange cross indicates that the number of allele-specific reads at the corresponding genomic region is ≤10 (i.e. not AS informative); the yellow dotted line is the expected proportion of allele-specific reads coming from allele A. (**c**) GC content and mappability of the region. These data predict a duplication with four-copies and an allelic configuration of ABBB. Although the TReCs at some regions are highly affected by mappability and GC content, AS-GENSENG still makes the correct CNV call. This result illustrates the method's favorable sensitivity for detecting duplications from noisy regions, by employing simultaneous bias correction and jointly using both read-count and allele-specific information in the inference.

HTS captures multiple sources of information in one experiment. Inspired by the successful integration of probe intensity and SNP genotypes in array-based CNV calling, here we develop an analogous method for HTS-based CNVs detection. Figure 1 provides an overview of our method. First, AS-GENSENG jointly exploits patterns in both Total Read Count (TReC) and Allele-Specific Read Count (ASReC) signals. While TReC is analogous to the total intensity from SNP arrays, ASReC is analogous to the allelic intensity from SNP arrays with expected patterns for each copy number state (Table 1). A CNV is indicated by higher- or lower-than-expected TReC and deviated ASReC values in comparison with copy-number 2 regions. Various sources of experimental bias are simultaneously accounted for in the CNV calling process (known biases accounted for via a covariate method and unknown biases via the over-dispersion parameter and the noise component of a mixture model) (54). Figure 2 shows an example CNV of copy-number 4 (enclosed by vertical lines) flanked by regions with copy-number 2. After accounting for bias (Figure 2c), the TReC in the enclosed region is approximately two times higher than that of the flanking region (Figure 2a), supporting a duplication of copy-number 4. The ASReC in the enclosed region is 0.25 and deviates from 0.5 in the flanking region, supporting copy-number 4 with an allelic configuration of ABBB (Figure 2b). Additional examples can be found in Supplementary Figures S3–S15 for CNVs with various copy numbers. These observations suggest that jointly exploiting patterns in both TReC and ASReC should improve the ability to detect both deletions and duplications.

**Table 1.**
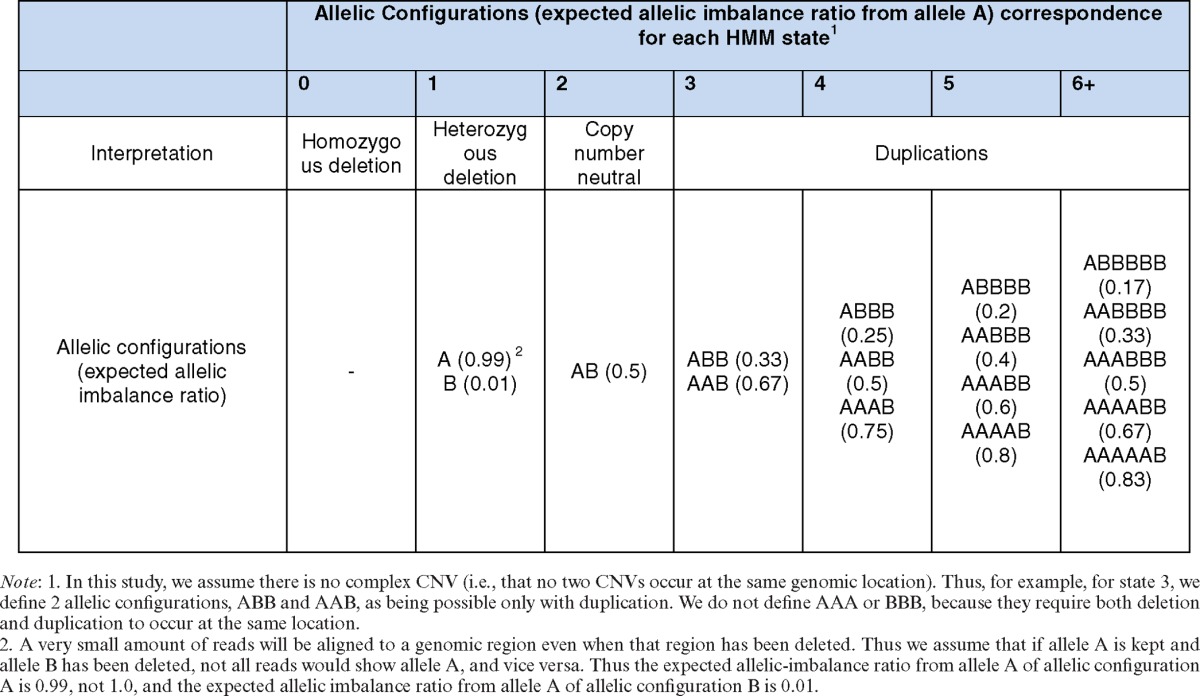
Allelic configuration correspondences for each HMM state

**Figure 3.**
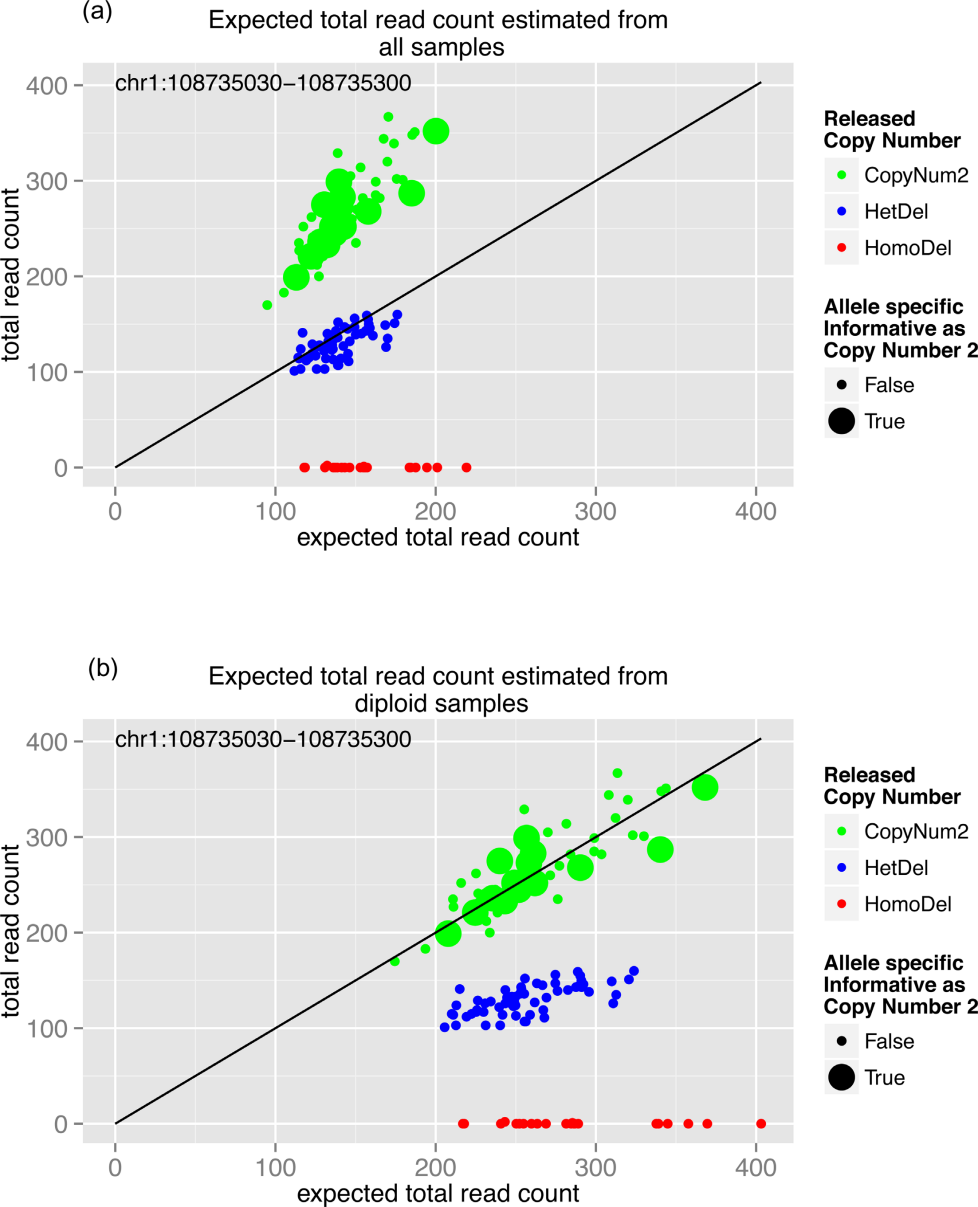
Example of a common deletion correctly identified by AS-GENSENG using WES data. The X-axis of each subfigure indicates the expected read-count at the exome-capturing target and the Y-axis indicates the observed read-count. The genomic position of the target is shown in the top-left corner and each dot in each subfigure indicates one sample. The color of the dots indicates copy-number information released by 1000GP: green denotes samples having copy-number 2; blue denotes samples having a one copy deletion (i.e. HetDel); and red denotes samples having a two-copy deletion (i.e. HomoDel). In addition, the size of dots indicates whether the sample should be estimated as copy-number 2 using the allele-specific information (i.e. AS informative as copy-number 2). This figure shows an example of a target where a common deletion exits. (**a**) When estimated from all samples the expected read-counts are not accurate because they are not the same as the observed read-counts for those diploid samples (the slope for the two-copy samples is greater than 1). (**b**) When estimated from the entire group of samples that have the same allele-specific information as copy-number 2, as shown on the right, the expected read-counts are accurate (the slope for the two copy samples in near 1). These results illustrate the accuracy of AS-GENSENG in detecting CNV on WES data even when a common CNV exists.

Furthermore, ASReC is useful for detecting common CNVs (i.e. >5% frequency) from WES data because it accurately identifies the reference copy-number 2 group without prior assumption of CNV frequency. Figure 3 shows an example of this phenomenon using 1000GP WES data (3,34), where >40% samples have a deletion (copy-number 0 or 1) over an exon target and the observed TReC values are compared to TReC expected for copy-number 2 reference. At least two approaches were developed to estimate the expected TReC. The first approach (50) uses the median or trimmed mean of all samples to estimate the expected TReC (47,48,50). However, given the common CNV, the median of all samples is far from the median of the copy-number 2 group and this approach leads to incorrect inference of the underlying copy numbers (Figure 3a). The second approach (48) constructs an optimized reference group of copy-number 2 by ranking the correlations of TReC between the reference and the test exomes and assuming that the CNV is not present in the reference. However, empirical results suggested that this approach had limited power for detecting common CNVs, presumably because the no-CNV assumption does not always hold in the selected reference exomes (48). In contrast, AS-GENSENG uses ASReC to properly identify the reference group of copy-number 2 (Figure 3b), yielding accurate estimation of expected TReC, and correct inference of the underlying copy numbers for this target.

### Data preparation

A brief description is provided below and detailed information can be found in the Supplementary materials.

#### Input files

We used WGS and WES data from HapMap individuals sequenced as a part of the 1000GP (3,34). The WGS samples included two HapMap samples of European ancestry (NA12891, NA12892), deeply sequenced (∼30×) using Illumina Genome Analyzer platforms. The WES samples include 324 individuals from four different populations sequenced to an average depth of ∼100× using Nimblegen and Agilent capture kits followed by Illumina sequencing. A consensus target-region list is defined by first intersecting the WES target design files with the NCBI CCDS database and then adding 50 bp at either side of each consensus target, resulting in 193 637 consensus exon targets and ∼47 Mbps captured in each WES sample. We obtained all alignment files from the 1000GP FTP sites (see the Web Resources section) (aligned using BWA (71) (v0.5.5) to hg19/NCBI37 (3,34)) and used hg19 coordinates throughout this study. In addition to alignment files, AS-GENSENG requires dense SNP genotypes in order to compute ASReC, and covariate files (genomic GC content and mappability) in order to account for their effects in CNV calling. We first obtained SNP genotypes from the HapMap project FTP sites (see the Web Resources section) (72) and then carried out imputation to obtain phased and dense SNP genotypes (>200 k for each sample). Given the SNP genotypes, we used the extractAsReads function in R/asSeq (see the Web Resources section) (73,74) to compute allele-specific alignments (i.e. aligned reads that could be confidently assigned to one particular SNP allele).

#### Total and allele-specific read counts

Following quality control of the alignment files, we extracted confidently aligned reads and computed TReC and AsReC given defined counting units. For WGS data, we divided the genome into sliding windows and computed TReC and AsReC in each window. Window size is a tuning parameter because of its influence on signal-to-noise-ratio of the read-depth data. In this study we used 500-bp-sliding windows with a sliding step of 100 bp, determined via both simulation and real-data analysis (see the Supplementary Methods). For WES data, we computed TReC and AsReC in each exon target. Although each region has only one TReC value, the ASReC in each region consists of two values: *o*^(*A*)^ (i.e. the total number of A-allele reads) and o^(*as*)^ = *o*^(*A*)^ + *o*^(*B*)^ (i.e. the number of A-allele reads plus B-allele reads). Note that *o*^(*as*)^ is smaller than TReC because many reads do not overlap a heterozygous SNP.

#### High-confidence CNV data

For sensitivity evaluation, we used previously published high-confidence CNVs in the same samples. For WGS samples, we used the high-confidence deletions (2200 for NA12891 and 2055 for NA12892) established by 1000GP (3,34,55) (see the Web Resources section). This data set had been validated using independent technologies as having high specificity (<4% FDR (false discovery rate)) and considered as best available high-confidence CNVs for these samples (3,34,55). For the 324 samples with WES data, WGS data were also available from the 1000GP (3,34), from which high-confidence genome-wide deletions have been established (see the Web Resources section). These genome-wide deletions were validated by independent technologies as having high specificity (FDR < 10%) by 1000GP (3,34). Similar to (65), we intersected these genome-wide deletions with the exon target list (≥1-bp overlap) to obtain high-confidence deletions in the target regions (i.e. exonic deletions). In total, there were 9192 exonic deletions for the 324 WES samples. As WGS is a more powerful technology in identifying CNVs than WES, these high-confidence exonic deletions provide both validity and accuracy in evaluating the sensitivity of exonic-CNVs identified by WES (65).

### Hidden Markov model

We developed an HMM classifying each genomic region (a window for WGS data or an exon target for WES data) to a copy-number state based on maximum *a posteriori* probability. In comparison with other segmentation methods such as circular binary segmentation (75), the use of HMM allows the joint analysis of multiple sources of information (TReC, ASReC, covariate values) as well as the modeling of integer copy numbers. Our HMM consists of multiple components (Supplementary Figure S16) with separate emission probability modules for WGS and WES data (Figure 1). A brief summary of the key HMM components is blow and details are available in the Supplementary data.

#### HMM state

The total number of hidden states is an input parameter and can be specified by users. For data sets used in this study, we set seven hidden states that, respectively, represent copy numbers of 0, 1, 2, 3, 4, 5 and 6 or more. In this work, the duplications with 6 or more copies were collapsed into one state because they were difficult to differentiate. To model ASCN, we defined several possible allelic configurations for each state (Table 1). For example, we defined AAB and ABB as the two possible allelic configurations for copy-number 3.

#### Transition probability

We model the state transitions using a first-order time-homogeneous Markov process (i.e. the state in one genomic region is affected only by the immediately previous region). Under this setting, the transition probability describes the probability of having a copy number change between two adjacent genomic regions. The transition probability is characterized as a square matrix, of which the dimension is the number of states and the (*i, j*) element is the probability of transition from state *i* to state *j*. We set the transition probability matrix according to our intuition that the copy number state is unlikely to change for nearby genomic regions but is likely to change for genomic regions that are far apart. Thus the self-transition probability (i.e. the diagonal values of the matrix) is much larger than the transition probability of transiting to other states. We assumed that most windows would have copy-number 2. Thus, the self-transition probability for state 2 would be higher than that of other states. In addition, the probability of transiting to state 2 would also be larger than the probability of transiting to other states. To handle the problem of varying distance between targets in WES data, we further modified each element in the transition matrix as suggested in Fromer *et al*. (42). The new element for the (*i, j*) element $a^{\prime} (i,j)$ would be a mixture of two original elements at (*i, j*) and (2, *j*), as $a^{\prime} (i,j) = e^{-d/D} \times a(i,j) + (1 - e^{- d/D} ) \times a(2,j)$, where *d* is the distance between two targets and *D* is the average distance between all targets.

#### Emission probability for WGS data

Emission probability specifies the likelihood of observing the TReC and ASReC inputs given the underling copy number and covariate information at the region. Given the underlying state, TReC and ASReC are independent and thus the likelihood can be factorized. The first factor is the probability of observing the TReC given the covariates and underling states, and the second factor is the probability of observing the ASReC from allele A (*o*^(*A*)^), given the overall ASReC and underlying states.

Following GENSENG (54), the likelihood of observing TReC is modeled by a mixture model of negative binomial distribution (NB) and uniform distribution. Known sources of bias such as GC content and mappability are included as the covariates of NB regression to account for their effect in CNV calling. Unknown sources of bias are accounted for by the NB overdispersion parameter and the uniform distribution (54). The method aggregates TReCs from all windows of one sample to estimate the expected TReC for each copy number state, with the assumption that the TReC would be proportional to the underlying copy number. The overdispersion parameter is estimated from the data using the Newton–Raphson method (detailed in the Supplementary Methods).

The likelihood of observing ASReC is modeled by a beta binomial distribution (BetaB), which is an extension of a binomial distribution to allow for possible overdispersion (73). Specifically, let *o*^(*A*)^ follow a binomial distribution with the number of trials *o*^(*as*)^ and the probability of success *p_S_*. If *p_S_* follows a beta distribution with parameters *α* and *β*, the resulting distribution of *o*^(*A*)^ is a beta-binomial distribution. This method adapts a commonly used strategy to parametrize a beta-binomial distribution by $\pi = \alpha /(\alpha + \beta )\;{\rm and}\;\theta = 1/(\alpha + \beta )$. Thus the likelihood of a beta-binomial distribution becomes

\begin{equation*} \begin{array}{*{20}l} {\ell (o^{(A)} ;o^{(as)} ,\pi ,\theta ) = } \\ {\left( {\begin{array}{*{20}c} {o^{(as)} } \\ {o^{(A)} } \\ \end{array}} \right)\frac{{\prod\nolimits_{k = 0}^{o^{(A)} - 1} {(\pi + k\theta )} \prod\nolimits_{k = 0}^{o^{(as)} - o^{(A)} - 1} {(1 - \pi + k\theta )} }}{{\prod\nolimits_{k = 1}^{o^{(as)} - 1} {(1 + k\theta )} }}} \\ \end{array}, \end{equation*}

where *π* is the expected proportion of AS reads from allele A (e.g. *π* = 0.33 for allelic configuration ABB). *θ* is a dispersion parameter. If there is no overdispersion, then *θ* = 0 and *o*^(*A*)^ follows a binomial distribution. In this work, we empirically set *θ* = 0.1. An underlying copy number has several possible allelic configurations (Table 1). We thus formulate the likelihood as the likelihood of a mixture distribution across all possible allelic configurations (e.g. for copy-number 3, the likelihood would be $(\ell (o^{(A)} ;o^{(as)} ,0.33,0.1) + \ell (o^{(A)} ;o^{(as)} ,0.67,0.1))/2$).

Taken together, the emission probability becomes $e(i,j) = \frac{c}{R} + (1 - c)\Pr (o_i^{{\rm all}} |q_i = j,x_i )\Pr (o_i^{(A)} |o_i^{as} ,q_i = j)$, where *e*(*i, j*) is the emission probability of the *i*th genomic region given the underlying copy number *q_i_* = *j* (0 > *j* > 6); *c* is the proportion of random uniform component which is constant for all states; *R* is the maximum read count; 1/*R* is the uniform density; $(o_i^{{\rm all}} ,o_i^{as} ,o_i^{(A)} )$ is the input observations tuple representing the TReC, total ASReC and ASReC from allele A of the *i*th genomic region, respectively; and *x_i_* is the input covariates for the *i*th genomic region. For data sets used in this study, *c* was set at 0.01 and was determined empirically by initializing the model with varying values of *c* and identifying the maximizer of the data likelihood.

#### Emission probability for WES data

The quantitative relationship between underlying copy number and read-count data is additionally distorted by target- and sample-specific biases in exome capture, which requires data normalition prior to computing emission probability. Our normalization belongs to the reference-set category of methods but we developed a new procedure of using ASReC for accurate identification of the reference-set. This procedure consists of five steps: (i) for each sample and each target, compute the ratio between TReC of the given target and the sum of TReC of all targets given the sample. This results in a target-by-sample matrix of normalized TReC values; (ii) for each target but cross all samples, use R/MixTools to cluster samples based on the normalized TReC. (iii) Use ASReC to compute the probability of being copy-number 2 for each sample by dividing the BetaB likelihood of being copy-number 2 with the sum of Beta likelihoods of being each copy number (0,1,2, …6+). The BetaB likelihood is computed as before (see Emission probability for WGS data: likelihood of ASReC). (iv) For each cluster, compute the average probability of being copy-number 2 of all samples belonging to the cluster. Compare and choose the cluster with the largest probability of being copy-number 2 as the reference group. (v) Compute the median of the reference group as the best estimate of the expected TReC. At this end, we are ready to incorporate the expected TReC into the HMM framework as before (see Emission probability for WGS data: likelihood of TReC) and use of all available information in the data to infer the underlying copy number.

#### HMM training and inference of total copy number

HMM training provides the maximum-likelihood estimate of the HMM parameters. To improve computational efficiency, transition-probability parameters were specified using prior knowledge and user preference (54), and emission probability parameters were estimated using the Baum–Welch algorithm (76). Using the estimated parameters, we compute the posterior probability of each genomic region belonging to a particular state and assign the most likely state for each region. The confidence score is computed as the sum of the posterior probabilities in regions spanned by a CNV.

#### Inference of ASCN

We assign the most likely ASCN given the most likely copy number call. For example, if the most likely copy number for a variant is 3, AS-GENSENG chooses between ABB and AAB. It first selects windows with ASReC larger than a threshold (10 in this study) in the region and computes the average ASReC of selected windows. If no window were selected, we would not infer ASCN because the ASReC is not informative. Otherwise, we would compute the likelihood of AAB and ABB using BetaB distribution and choose the one with the largest likelihood as the inferred ASCN.

### Performance evaluation: WGS data

A short description is provided below and detailed procedures can be found in the Supplementary materials.

#### Competing methods

We used both simulation and empirical data to assess the performance of AS-GENSENG in comparison with state-of-the-art WGS methods including GENSENG (54), CNVnator (56) and ERDS (41). We used the recommended parameters and QC filters for competing methods. For example, with CNVnator (56) we used the q0 filter that filters out any predictions that have >50% reads with zero-valued MAPQ (i.e. reads with multiple mapping locations). With ERDS (41) we removed deletions that are <10 kb and do not have supporting read-pairs. The methodological differences between all four WGS methods compared here are detailed in Supplementary Table S4a. The differences between AS-GENSENG and GENSENG are highlighted in Supplementary Table S4b. AS-GENSENG differs from existing methods mainly in its incorporation of AS information, simultaneous bias correction and ability to detect ASCN in addition to total copy number.

#### Simulation study

Two sets of simulation were conducted. In the first simulation, we generated read-count data (i.e. TReC and ASReC) in a single sample by using the chromosome 1 WGS data from NA12891 as the template and implanting 200 CNVs by modifying read-counts within CNVs. Briefly, TReC was simulated using a negative binomial regression model taken into account the effects of GC content and mapability, and ASReC was simulated using a beta-binomial distribution. In the second simulation, we generated paired-end reads from a pair of CNV-containing hypothetical chromosomes, created by implanting 200 artificial CNVs into chromosome 1 of the human reference genome (hg19). Artificial CNVs were created by modifying the sequence within each variant according to its copy number. Based on the hypothetical chromosomes, we applied SAMTools's wgsim with default values to generate 100-bp paired-end reads. In total, 50 millions read pairs were generated and yielded ∼40× coverage. We simulated allele-specific reads using heterozygous SNPs from NA12891. In each simulated data set, the 200 implanted CNVs included 60% deletions with copy numbers 0–1 and 40% duplications with copy numbers 3–6, median size 3000 bp.

#### Evaluation metrics

To evaluate sensitivity and (FDR), we focused on autosomal CNVs and intersected the predicted CNVs of different methods with the known CNVs. The known CNVs (Supplementary Figure S17) are either the simulated ground-truth CNVs for the simulated data or the 1000GP-released high-confidence deletions for the empirical data. Sensitivity was calculated as the proportion of known CNVs overlapped by predicted CNVs with the correct CNV type. Following 1000GP, we defined CNV type as deletions (integer copy-number 0 or 1) or duplications (integer copy number ≥3). Sensitivity for detecting duplications in the two HapMap individuals was not evaluated because of the lack of high-confidence duplications (3,34). The FDR for the simulation data was calculated as the proportion of predicted CNVs not overlapped with the known CNVs. Because the true negatives for the two HapMap individuals are not known, we used the total number of base pairs and the total number of calls as a surrogate measure for specificity. A 50% reciprocal overlap was used as the overlapping criterion in all WGS comparisons. To evaluate the AS information, we reported the ASCN set. In the simulation, we had the ground-truth ASCN set (ASReC > 10). Thus we reported the sensitivity and FDR based on the ground-truth ASCN set and compared with the values on the entire set. With the empirical data, we reported the number of detected ASCNs.

#### Performance on low coverage data

CNV detection performance depends on sequencing coverage, especially for read-depth-based methods. In the comparative analyses conducted in this study, both simulated (40×) and the 1000GP data (>30×) had high coverage. Thus we carried out a computational experiment in order to identify the lower bound on sequencing coverage that AS-GENSENG can handle. In this experiment, we first used Picard's DownSampleSam.jar tool to down-sample the high coverage data to varying coverage of 30×, 20×, 10× and 5× and next applied AS-GENSENG to each resulting data set and evaluated the performance using the same metrics.

### Performance evaluation: WES data

A short description is provided below and detailed procedures can be found in the Supplementary materials.

#### Competing methods

We used both simulation and empirical data to assess the performance of AS-GENSENG in comparison with state-of-the-art WES methods including Conifer (49), XHMM (42) and ExomeDepth (48). We used the recommended parameters and QC filters for each competing method. For example, with Conifer, we removed five SVD components for detecting common CNVs (5, 10 and 20% frequency) and 10 components for detecting rare CNVs (1% frequency) (49). With XHMM, we used the default value 30 for CNV quality threshold (42). Methodological differences are detailed in Supplementary Table S5. The primary novel aspect of AS-GENSENG is its use of allele specific information in the modeling and its ability to detect ASCN.

#### Simulation study

We used chr11 WES data of HapMap sample HG00264 as the template and simulated read-counts (i.e. TReC and ASReC) for 100 WES samples. This simulated data set contained 1000 deletions and 1000 duplications with allele frequencies of 1 and 5% and 200 deletions and 200 duplications with allele frequencies of 10 and 20%. See Supplementary Figure S18 for detailed description of the simulation pipeline.

#### Evaluation metrics

In addition to metrics used for WGS data, here we further applied the SuperArray Validation (SAV) (3,34) to evaluate the FDR for 1000GP WES data. The SuperArray integrated available intensity data for HapMap samples from three array platforms (Affymetrix 6.0, Illumina 1 M and a custom Nimblegen aCGH array with 4 938 838 probes) into a high-density virtual array. A non-parametric testing procedure is developed to calibrate predicted CNVs using SuperArray. The rule of thumb of the procedure is that the intensity data of samples with lower underlying copy number tend to be lower than samples with higher underlying copy number.

### CNV validation using NanoString technology

In order to validate randomly selected deletions and duplications, we utilized an independent methodology, NanoString nCounter, a proven and high-throughput method for CNV verification (66–69). We focused on validating AS-GENSENG's ability to detect CNVs from WES data and NA12272 was randomly chosen from the 324 HapMap samples for which WES data were analyzed. Our first goal was to compare AS-GENSENG calls in sample NA12272 with the relative copy number estimated by NanoString (following the analysis method in (70)). It is important to note that, for each probe, NanoString requires samples known to be copy-number 2, so we relied on the absolute copy number reported in Conrad *et. al.* (2) to calibrate the NanoString calls (i.e. indicate which samples have copy-number 2). In addition, we would separate the calls with overlapping SNPs from the calls without overlapping SNPs to study the effect of using SNP information in CNV detection.

Our second goal was to compare AS-GENSENG calls in sample NA12272 with two other members of the trio (paternal: NA12272; maternal: NA12273; and child: NA10837) in order to identify Mendelian inconsistencies. DNA for each of these samples was acquired from the Coriell repository (see the Web Resources section) and used as input for the NanoString nCounter CNV assay, according to the manufacturer's instructions. In short, 600 ng of genomic DNA was fragmented to ∼500 bp by digestion with AluI and subject to a multiplex hybridization reaction involving all probes. We designed a custom NanoString probe set (using NanoString's nDesign Gateway software) targeting 11 deletions and 14 duplications predicted by AS-GENSENG, with each locus targeted by a single custom probe. The custom probes were 70–100 bp in length, each was placed in the middle of a targeted CNV and all satisfied the internal design parameters used by NanoString, such as good GC-content and not-overlapping segmental duplication or repetitive elements. The probe set also included eight negative control probes that target artificial sequences, and 10 normalization probes that target autosomal loci that are invariant in copy number. Data analysis was conducted as in (70).

## RESULTS

### CNV detection in whole-genome sequencing-simulation data

In order to assess the performance of our method for predicting CNVs from WGS data, we applied AS-GENSENG to two sets of simulated data. We first conducted 100 simulations of TReC and ASReC affected by implanted CNVs. We expected both the TReC and ASReC within the CNV-implanted windows to be affected. Since not every CNV region has enough allele-specific reads to provide informative ASReC, in this work, we defined a CNV region informative for ASCN (i.e. ASCNV) if the ASReC was >10. Supplementary Figures S1 and S2 show examples of simulations, including simulated copy number, TReC at each simulated genomic region, simulated ASCNs and covariates (GC content and mappability). For each simulation, we implanted 200 CNVs (122 deletions and 78 duplications on average); 27 were ASCNVs (12 deletions and 15 duplications) on average. We estimated sensitivity and FDR by intersecting the AS-GENSENG-predicted CNVs with the implanted CNVs (using ≥50% reciprocal overlapping as the criterion; illustrations of sensitivity and FDR calculation are shown in Supplementary Figure S17). Results are detailed in Supplementary Table S6 and are summarized below.

On average, AS-GENSENG predicted 180 ground-truth CNVs (90% sensitivity) from each simulated data set, including 113 deletions (92% sensitivity) and 68 duplications (86% sensitivity). Regarding ASCNVs, AS-GENSENG predicted 25 ground-truth ASCNVs (93% sensitivity), including 11 AS deletions (98% sensitivity) and 14 AS duplications (90% sensitivity). Therefore, we observe slightly higher sensitivity for detecting AS events and, furthermore, the FDR for ASCNVs is 0.3%, much lower than that for standard CNVs (14.5%). This lower FDR results from the fact that read-count signals alone are vague for differentiating between copy-number 2 and copy-numbers 3 or 1. Without ASReC, the algorithm could make false-positive calls. However, the difference in allele-specific proportion is much clearer between copy-number 2 and copy-numbers 3 or 1 (0.5 in copy-number 2 compared to 0.33 or 0.67 in copy-number 3, and 0.99 or 0.01 in copy-number 1). As a result, the FDR of ASCNVs is much lower.

We next simulated sequencing reads affected by implanted CNVs. We simulated 200 CNVs (119 deletions and 81 duplications), 62 were ASCNVs (15 deletions and 47 duplications), and estimated sensitivity and FDR using the same criterion. We first compared AS-GENSENG's performance for standard CNVs versus ASCNVs and then compared AS-GENSENG to CNVnator and ERDS for the ability to detect standard CNVs. Results are detailed in Supplementary Tables S7 and S8 and summarized below. For standard CNVs, AS-GENSENG predicted 193 ground-truth CNVs resulting in 97% sensitivity (117 deletions with 98% sensitivity and 76 duplications with 94% sensitivity). For ASCNVs, AS-GENSENG predicted 61 ground-truth AS-CNVs resulting in 98% sensitivity (15 AS deletions with 100% sensitivity and 46 AS duplications with 98% sensitivity), slightly higher than that for standard CNVs. Further, AS-GENSENG's FDR for ASCNVs is 4%, lower than that for standard CNVs (7%). Compared to other methods on the ability to detect standard CNVs, AS-GENSENG had the highest sensitivity and lowest FDR (sensitivity 6% higher than CNVnator and 1% higher than ERDS; FDR 8% lower than CNVnator and 23% lower than ERDS).

In summary, simulation results suggested that incorporating ASReC improves the sensitivity and specificity of CNV detection.

### CNV detection in whole-genome sequencing real data

To further evaluate the performance of our method for WGS data, we analyzed 1000GP (3,34) data. We applied AS-GENSENG, GENSENG (54), CNVnator (56) and ERDS (41) to the high-coverage WGS data for samples NA12891 and NA12892 and compared the predicted CNVs to a high-confidence, published data set available for these samples (3,34,55) (2200 deletions in NA12891 and 2055 deletions in NA12892 but no high-confidence duplications available). AS-GENSENG differs from existing methods mainly in its incorporation of AS information for both deletions and duplications (in comparison with GENSENG, CNVnator and ERDS) and its simultaneous bias correction (in comparison with CNVnator and ERDS). The methodological differences between these methods are detailed in Supplementary Table S4.

First, we compared AS-GENSENG to GENSENG and CNVnator, both relying only on TReC for CNV detection. As shown in Table 2(a), the sensitivity for detecting deletions by AS-GENSENG was 56% for NA12891 and 53% for NA12892, which is higher than GENSENG (50% for NA12891 and 49% for NA12892) and CNVnator (37% for NA12891 and 34% for NA12892). We also found several examples of high-confidence deletions that were missed by GENSENG but were recovered by AS-GENSENG (Supplementary Figures S3–S15). Due to the relatively high level of noise in the TReC of these relatively small deletions (<10 windows), the TReC signal by itself does not provide enough evidence for GENSENG to call deletions. However, the imbalance of ASReC signals in these examples strongly implies underlying CNVs. Thus, by incorporating ASReC with TReC, AS-GENSENG successfully recovered these deletions.

**Table 2.**
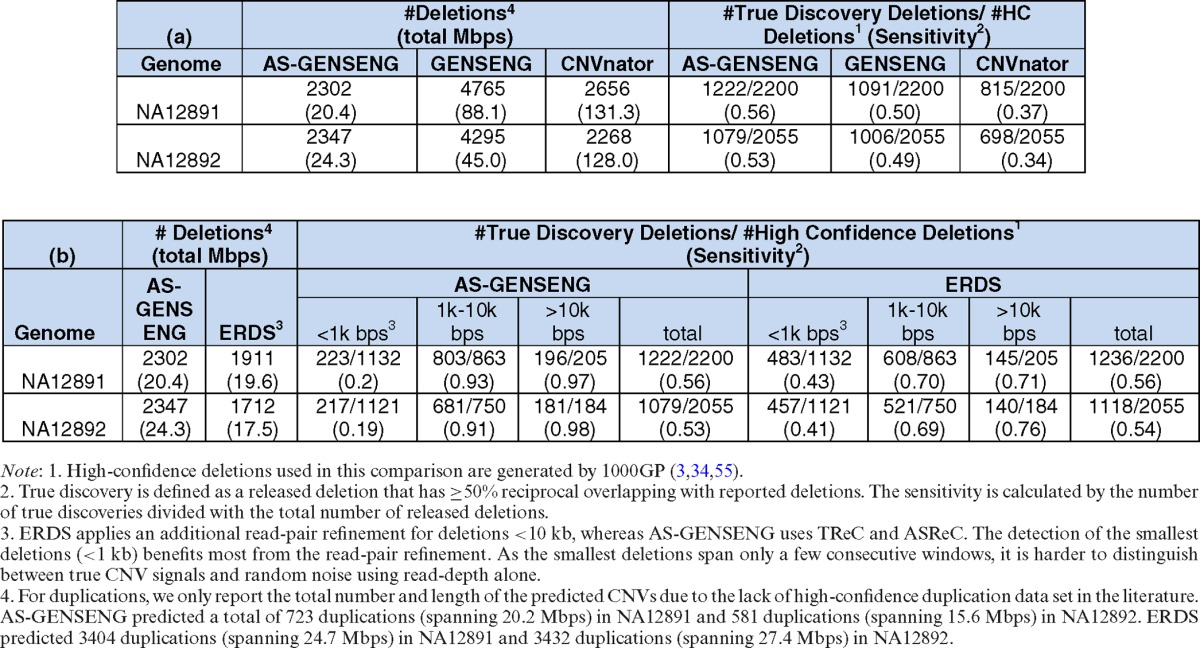
Performance assessment based on WGS data of two HapMap samples

We then compared the specificity of various methods. Because the high-confidence CNV data set does not provide information on the true negatives for assessing specificity, we used the volume (i.e. the total number and total base pairs) of the predicted CNVs as a surrogate measurement of specificity. As shown in Table 2(a), the volume of AS-GENSENG is much smaller than GENSENG and CNVnator, suggesting improved specificity. Second, we compared AS-GENSENG to another integrated method, ERDS. ERDS incorporates the rate of heterozygous SNPs in detecting deletions and further refine the smallest deletion calls (<10 kb) using read-pair information; but ERDS relies only on TReC in detecting duplications. Thus in our sensitivity evaluation, we stratified the comparative analysis by the size of the high-confidence deletions in three categories (<1 kb,1–10 kb and >10 kb). Finally, we applied AS-GENSENG, CNVnator and ERDS to the WGS data from a HapMap trio (NA12891, NA12892, NA12878) and computed the rate of Mendelian inconsistencies as a measure of specificity. By intersecting CNV calls in the child (NA12878) with CNV calls in the parents, we found that AS-GENSENG had the lowest Mendelian error rate (25%) among all CNVs predicted in the child (23% for deletions and 28% for duplication), whereas CNVnator had 48% Mendelian errors (47% for deletions and 52% for duplications) and ERDS had 55% Mendelian errors (48% for deletions and 57% for duplications).

As shown in Table 2(b), in the >10-kb category when both ERDS and AS-GENSENG incorporate SNP information with TReC, AS-GENSENG achieved 26% higher sensitivity in NA12891 (97 versus 71%) and 22% higher sensitivity in NA12892 (98 versus 76%). In the 1–10-kb category even after ERDS applied read-pair information for call refinement, AS-GENSENG achieved 23% higher sensitivity in NA12891 (93 versus 70%) and 22% higher sensitivity in NA12892 (91 versus 69%). In the <1-kb category AS-GENSENG had lower sensitivity than ERDS, which can be attributed to two factors: (i) AS-GENSENG can only detect CNVs of twice or more of the window size (i.e. >600 bp), whereas ERDS does not have this limitation; (ii) within the category of CNVs >600 bp and smaller than 1 kb, ERDS has advantage by additionally using read-pair information. We then examined the volume of CNV calls as a surrogate measure of specificity. AS-GENSENG predicted slightly higher number of deletions than ERDS (2303 versus 1911 in NA12891 and 2347 versus 1712 in NA12892), suggesting comparable specificity; but predicted a much smaller number of duplications than ERDS (723 versus 3404 in NA12891 and 581 versus 3432 in NA12892), suggesting improved specificity.

In summary, when applied to high-coverage WGS data, AS-GENSENG outperforms existing methods for detecting deletions that are >1 kb. It gives the best sensitivity (∼5% higher than GENSENG, ∼20% higher than CNVnator and more than 20% higher than ERDS) and among the best specificity (only slight larger than ERDS in the deletion calls). These results suggest that incorporating AS information improves the accuracy of CNV detection. Further, in regard to ASCNVs, AS-GENSENG is the only method that can predict ASCN call from WGS data in germline DNA samples. In this experiment, AS-GENSENG predicted 576 AS deletions and 205 AS duplications in NA12891, 664 AS deletions and 173 AS duplications in NA12892.

As expected, we find that the higher the sequencing coverage, the better the performance for AS-GENSENG to detect CNVs. The lowest bound of sequencing coverage that AS-GENSENG still achieves a reasonable sensitivity is 10× (Supplementary Tables S9 and S10). At a low coverage of 5×, AS-GENSENG's sensitivity is remarkably reduced (i.e. 29% reduction in detecting deletions and 15% reduction in detecting duplications in simulation study, and 11% reduction in deletions for 1000GP WGS data).

### CNV detection in whole-exome sequencing-simulation data

In order to calibrate the performance of our method for WES data, we applied AS-GENSENG to simulated data sets and evaluated the sensitivity and FDR by comparing the predicted CNVs with the implanted ground-truth CNVs (Supplementary Table S11). In particular, we evaluated AS-GENSENG's ability to detect CNVs at varying allele frequencies. Following the criterion of rare CNVs (<5% in the population (42)), we simulated both rare (1%) and common CNVs (5, 10 and 20%). First, we calculated sensitivities (using ≥1-bp overlap) for the entire set of implanted CNVs or ASCNVs. The sensitivities ranged from 81 to 91% for various CNV frequency settings and there was no remarkable difference in sensitivity between the rare CNV and common CNV sets. For example, the respective sensitivities are 0.89 for deletions and 0.91 for duplications on the 1% CNV frequency set, and 0.85 for deletions and 0.90 for duplications on the 20% CNV frequency set. Regarding ASCNVs, all sensitivities were >90% and better than the corresponding regular CNV values. For example, with a CNV frequency of 5%, we observed a 10% improvement for deletions and 2% improvement for duplications. Second, we evaluated FDR and found that AS-GENSENG had very low FDR for both CNV and ASCNV (most <1%). These results suggest that, when applied to WES data, AS-GENSENG can robustly detect both rare and common CNVs at varying frequencies and that incorporating ASReC improves the accuracy for CNV detection.

Next, using the same simulated WES data sets, we compared AS-GENSENG with three state-of-the-art methods, XHMM (42), Conifer (49) and ExomeDepth (48) (see Supplementary Table S5 for a detailed method comparison). XHMM and Conifer use PCA/SVD-based normalization (49). ExomeDepth uses a reference-based normalization with an optimized reference-set (48). AS-GENSENG uses a reference-based normalization and its novelty is its explicit use of ASReC to identify the correct reference group of copy-number 2, which is critical for data normalization and the detection of common CNVs of unknown frequencies. The results of the comparative analysis are summarized in Supplementary Table S12. We find that the sensitivity of AS-GENSENG is higher than XHMM, Conifer and ExomeDepth for all CNV frequencies, especially in detecting common CNV (AS-GENSENG sensitivity is 89.6% for CNV frequency 1% while XHMM is 87.7%, Conifer is 53.3% and ExomeDepth sensitivity is 82.4%; AS-GENENG sensitivities are higher than 80% for CNV frequency >5% while XHMM sensitivities are less than 6%, Conifer sensitivities are less than 30% and ExomeDepth sensitivities are less than 61%). While AS-GENSENG demonstrated consistently good sensitivity across frequency categories, the sensitivities of XHMM, Conifer and ExomeDepth decrease in common CNVs. For the most common CNVs (frequency = 20%), AS-GENSENG was >100× more sensitive than XHMM, 60X more sensitive than Conifer and 2.5× more sensitive than ExomeDepth (AS-GENSENG sensitivity is 87.4%; XHMM is 0.2%; Conifer is 1.3%; ExomeDepth is 34.2%).

Presumably, at higher CNV frequencies, CNV signals may have stronger contributions to the very variance components that are excluded by the SVD method with an arbitrary threshold (65); and TReC may not identify the true reference copy-number 2 set. We find that the FDR of AS-GENSENG is <1% for all settings, the FDR of XHMM is <2% for most settings, the FDR of Conifer is <2% for most settings and the FDR of ExomeDepth is <1% for all settings, suggesting similar, high specificity. In summary, these results suggest that reference-based normalization combined with assumption-free identification of the copy-number 2 reference, such as using ASReC as implemented in AS-GENSENG, is critical for robust detection of common CNVs at varying frequencies that cannot be known *a priori*.

### CNV detection in real whole-exome-sequencing data

To further evaluate the performance of CNV detection in WES data, we applied AS-GENSENG, Conifer (49), XHMM (42) and ExomeDepth (48) to the WES data of 324 HapMap samples (Table 3). The total numbers of CNVs called from AS-GENSENG, Conifer and XHMM are comparable. AS-GENSENG predicted 4839 deletions and 2648 duplications in total from the 324 samples, while Conifer predicted 2194 deletions and 3450 duplications, XHMM predicted 3006 deletions and 3660 duplications. ExomeDepth predicted 74 463 deletions and 47 816 duplications, which is similar to literature results using ExomeDepth or ∼300 CNVs per sample with around two thirds of CNVs as deletions, ((48); the Web Resources section). To evaluate sensitivity, we compared these call sets with the high-confidence exonic deletions as described in the Materials and Methods section and then repeated this analysis separately for rare (<5% frequency) and common (>5% frequency) high-confidence exonic deletions. Sensitivity for duplications was not evaluated due to the lack of high-confidence duplication call sets in the literature (3,34). Key results of the sensitivity evaluation are summarized below.

**Table 3.**
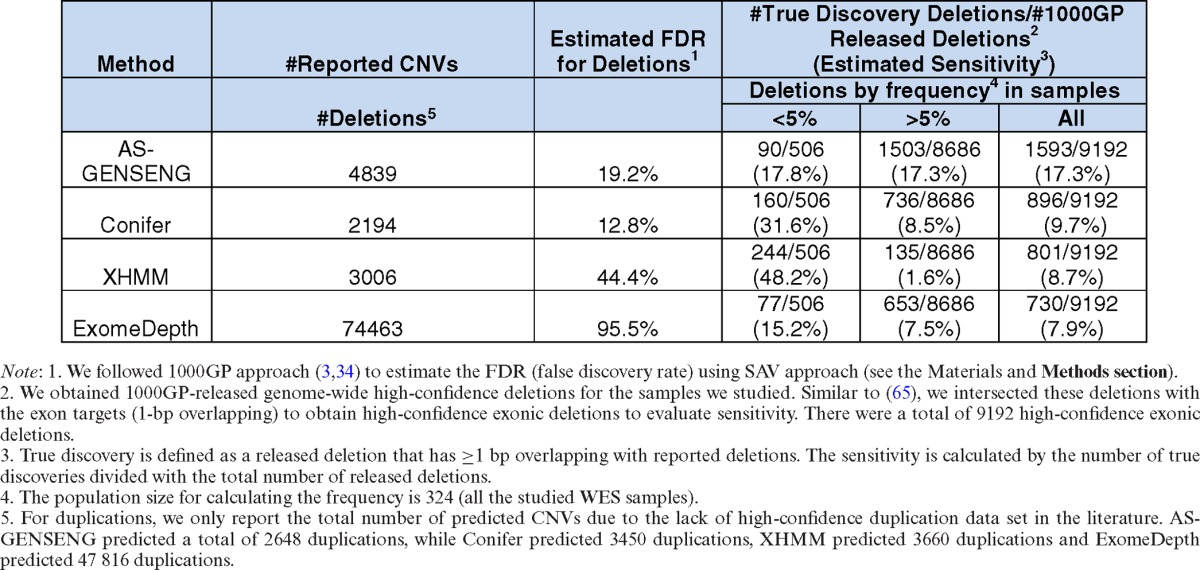
Performance assessment based on whole-exome sequencing data of 324 HapMap samples

First, AS-GENSENG demonstrated the highest overall sensitivity for detecting high-confidence exonic deletions (7.6% higher than Conifer, 8.6% higher than XHMM and 9.4% higher than ExomeDepth). Second, the sensitivity estimates of AS-GENSENG are consistent across CNV frequency categories, whereas the sensitivity estimates of the other three methods varied considerably between rare and common deletions. Third, for rare deletions, XHMM had the highest sensitivity; and for common deletions, AS-GENSENG had the highest sensitivity. Fourth, we note that the relatively low sensitivities of all methods observed in our evaluation are not surprising; similar results have been reported in recently published independent studies (65,77). This may reflect technological differences between WES (from which the CNV call sets were generated) and WGS (from which the high-confidence exonic deletions were obtained). Typically, WGS is more powerful in detecting CNVs and does not suffer from the additional systematic biases introduced in the exome capturing step (65).

To evaluate FDR, we followed the SAV approach developed by 1000GP (3,34) (see ‘Evaluation metrics’). For deletions, there were 334 predicted deletion regions (where at least one sample has deletion call) in the AS-GENENG calls set and 32 of these regions had *P*-value >0.5 based on the Wilcoxon Rank Sum test, which yielded an FDR of 19.2%. Similarly, the FDR was 12.8% in Conifer calls set (3 with *P*-value >0.5 among a total of 47 regions), 44.4% in XHMM calls set (16 with *P*-value >0.5 among a total of 72 regions) and 95.5% in ExomeDepth calls set (747 with *P*-value >0.5 among a total of 1564 regions). For duplications, there were 169 predicted duplication regions (where at least one sample has duplication call) in the AS-GENSENG calls set and 20 regions had *P*-value >0.5, which yielded a 23.7% FDR. Similarly, the FDR was 50.5% in Conifer calls set (27 with *P*-value >0.5 among a total of 107 regions), 14.9% in XHMM calls set (14 with *P*-value >0.5 among a total of 188 regions) and 85.8% in ExomeDepth calls set (391 with *P*-value >0.5 among a total of 911 regions). With SAV, the FDR is not defined for CNVs in individual samples but rather to CNV regions in the collection of all samples, and therefore we did not evaluate FDR stratified by frequency as we did in the sensitivity comparison.

In summary, on the 324 WES samples evaluated in this study, AS-GENSENG demonstrated the best sensitivity for detecting common deletions and comparable specificity to other state-of-the-art methods. The performance of AS-GENSENG was consistent across CNV frequency categories, which can be attributed to its ability to accurately identify the reference copy-number 2 group two using ASReC and free of assumptions (see Figure 3 and Supplementary Figures S19–S29 for examples). As expected, XHMM had the best sensitivity for detecting rare deletions because its PCA normalization and HMM parameters were optimized to detect rare variants, assuming that most variation in read-depth was due to noise. Finally, in regard to ASCN, AS-GENSENG is the only method that could predict ASCN from WES data. In this experiment, AS-GENSENG detected 2091 ASCNV (525 AS deletions and 1566 AS duplications) from the 324 HapMap samples.

### CNV validation using NanoString technology

**Figure 4.**
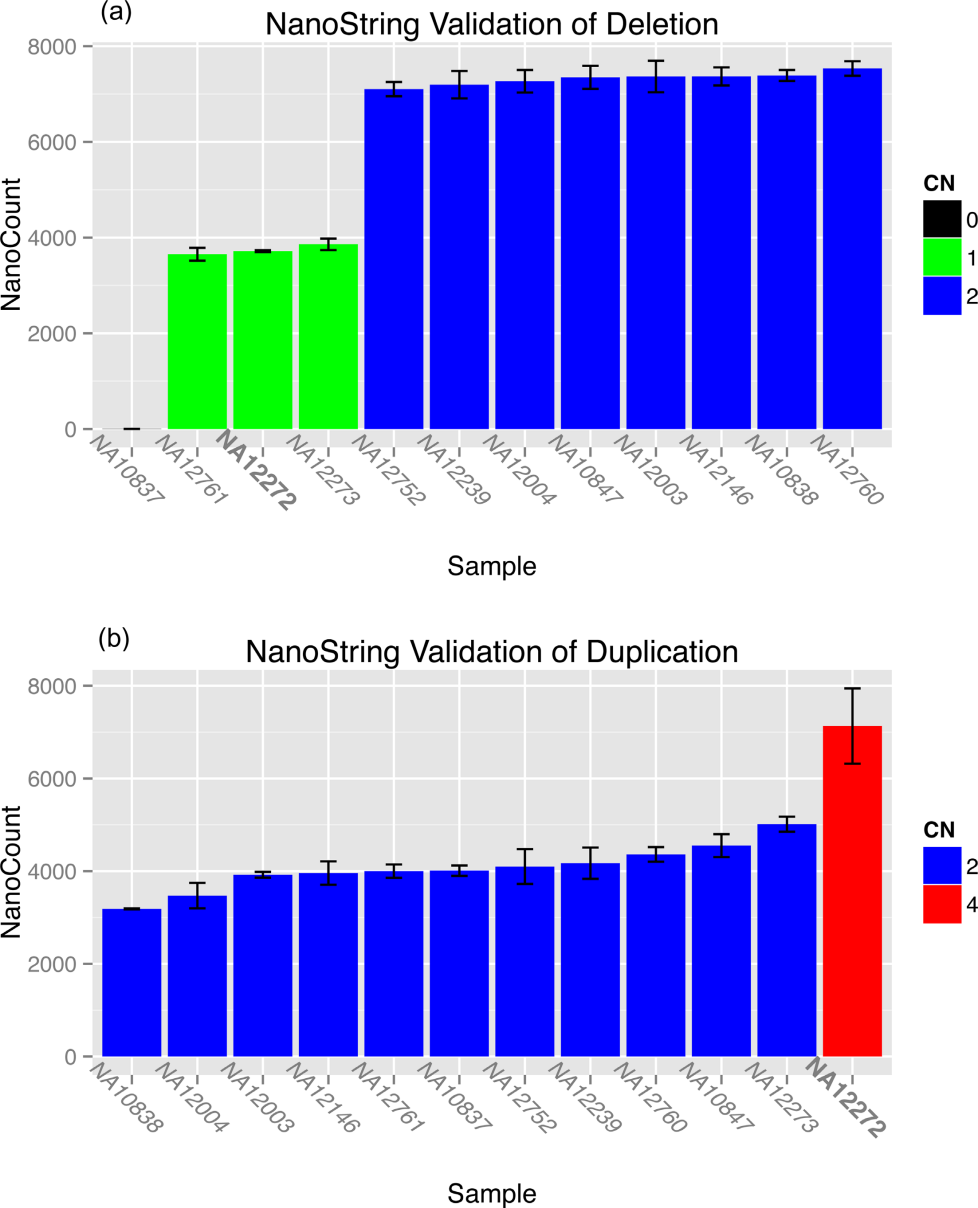
Examples of NanoString nCounter Technology Validated AS-GENSENG CNV Calls. There are two examples of a validated deletion call (**a**) and a validated duplication call (**b**) made by AS-GENSENG in sample NA12272 shown in the figure. The height of bar represents the NanoString normalized count. AS-GENSENG studied sample NA12272 is highlighted in bold in the X-axis. The error bar indicates data from two runs of the validation procedure. (a) In the deletion call, AS-GENSENG made a copy-number 1 deletion in NA12272, and it is validated because the count measured from NA12272 is half of the count from samples having copy-number 2. (b) In the duplication call, AS-GENSENG made four-copy number duplication in NA12272, and it is validated because the count measured from NA12272 is twice of the count from samples having copy-number 2.

We decided to use an independent methodology (NanoString) to validate randomly selected deletions and duplications predicted from WES data. First, we compared AS-GENSENG calls in sample NA12272 with the relative copy number estimated by NanoString (following the method in (70)). It is important to note that, for each probe, NanoString requires samples known to be copy-number 2, so we relied on the absolute copy number reported in Conrad *et. al.* (2) to calibrate the NanoString calls (i.e. indicate which samples have copy-number 2). A deletion was validated if its NanoString count is at least 50% smaller than the median of NanoString counts of copy-number 2 samples (Figure 4a). Among the 11 randomly selected AS-GENSENG deletions, NanoString identified 9 as true deletions, yielding a validation rate of 82% (or 18% false discovery). A duplication was validated if its NanoString count is at least 50% larger than the median of NanoString counts of copy-number 2 samples (Figure 4b). Among the 14 randomly selected AS-GENSENG duplications, NanoString identified 12 as duplications, yielding a validation rate 86% (or 14% false discovery). The results obtained from NanoString validation are similar to the results based on our SAV described above.

There are multiple possible explanations for the AS-GENSENG calls that failed to validate. First, it is possible that AS-GENSENG produced false positive calls. Second, it is also possible that false negatives exist in the Conrad *et. al.* data set (2) leading to improper calibration to copy-number 2. Third, and perhaps most likely, the issue could simply be a matter of probe placement, since we tested just one probe per CNV region. We decided to test a limited number of probes per CNV in order to maximize the number of CNVs tested, however, this also limits the accuracy for each single region. The probe size (<100 bp) is also much smaller than the tested region (>1000 bp). Furthermore, due to the limitation of the probe design, the probes are not always placed in the middle of the region, so the issue may be due to CNV resolution. Second, in order to evaluate the contribution of SNP information, we repeated the analysis separately for CNV calls with and without SNPs. SNPs were found in all 11 deletion calls. In the 14 duplication calls, 10 had SNPs of which 9 were validated (90% validated); whereas 4 did not have SNPs of which 3 were validated (75% validated). The increased validation rate in duplications with SNPs suggests that ASReC improved detection accuracy. Finally, as a secondary analysis, we also compared AS-GENSENG calls in sample NA12272 with the NanoString estimated CNV calls in the complete trio containing NA12272 (paternal: NA12272; maternal: NA12273; and child: NA10837). For each CNV, we looked for Mendelian inconsistencies in the NanoString copy number estimates. We found that among the 11 AS-GENSENG deletions, 10 were consistent. We found that all 12 AS-GENSENG duplications were consistent in this trio. If we assume that the NanoString copy number estimates in the parents were accurate, this analysis suggested 9% Mendelian error for deletions and 0% Mendelian error for duplications.

## DISCUSSION

We have developed an integrated and novel method (AS-GENSENG) that exploits the rich information in both total (TReC) and allele-specific read-depth (ASReC) to detect CNVs and ASCNVs from both WGS and WES data. We use HMM to infer the underlying integer copy numbers and combine the joint analysis of TReC and ASReC with simultaneous bias correction in data likelihood. The WGS module of AS-GENSENG is applicable to a single genome, while the WES model is applicable to large-scale exome data. To our knowledge, AS-GENSENG is the first tool capable of detecting ASCNVs from HTS data in germline DNA samples.

Analogous to the previous success with array-based CNV calling, we have demonstrated that joint analysis of TReC and ASReC not only allows the estimation of ASCN but also improves the estimation of total copy number (e.g. 1 copy deletion, 3 copy duplications). We show through numerous examples, using both WGS and WES data, that incorporating ASReC improves the performance of CNV detection. In addition, one novel component of AS-GENSENG is the use of beta-binomial distribution to incorporate allele-specific information. This approach, applied to model both deletions and duplications rather than only deletions, does not restrict the analysis to inbred genomes (59) and does not require human effort to call ASCNV (37). We have also shown that ASReC can be leveraged to accurately identify the copy-number-2 reference-group from an exon target, crucial for accurate CNV calling in WES data. Although previous studies (49) have applied sophisticated analysis techniques to deal with the common CNV problem, we have shown that using ASReC is a novel and effective strategy to tackle this problem.

We are aware of several limitations with AS-GENSENG and have recommend alternative strategies. First, we focused on the accurate detection of simple CNVs and computed TReC using reads with unambiguous mapping in the reference genome. This approach results in lower power to detect complex CNVs within repeated sequences. For detecting CNVs in repeat-rich region, we recommend the use of specialized methods that are capable of considering all mapping positions and handling the uncertainty of read mapping (60,64,78–81). Second, the WGS module of our method used a sliding window approach to compute TReC and ASReC. This approach results in lower power to detect CNVs that are <1 kb. For detecting deletions <1 kb, we recommend ERDS (41) or Genome STRiP (55) as these methods further utilize read-pair information for improved detection. A similar refinement pipeline using read-pair information will be implemented in a future release of AS-GENSENG. Third, while our WES module is robust against CNV frequency, its power for detecting rare exonic CNVs is lower than methods that are optimized for this class of variants. In this paradigm, XHMM appears to have superior sensitivity for detecting rare CNVs from WES data and the quality score provided by XHMM could be informative in downstream analyses in order to improve specificity (42). Finally, INDELs (insertions and deletions <50 bp (3,34,35)) could not be detected by AS-GENSENG and require specialized algorithms (35,82).

We aimed to conduct a comprehensive evaluation by comparing the performance of AS-GENSENG to multiple state-of-the-art methods. In order to provide an unbiased evaluation, we applied each method using its recommended parameters and quality control filters. Through independent evaluations conducted by the 1000GP, Genome STRiP (55) was regarded as the best performing among existing methods for WGS data. Genome STRiP is a multi-sample method and requires at least 20 or 30 samples (see ‘vii’ in the Web Resources section). In this study, we focused on detecting CNVs from a single genome and therefore did not compare AS-GENSENG with Genome STRiP.

We used multiple approaches (i.e. simulation, SAV, trio-analysis, NanoString) to evaluate FDR as it is more challenging to estimate without the knowledge of true false negative CNVs in the genome. For AS-GENSENG, although the absolute FDR observed in real data is higher than that observed in simulation, the relative FDR is still lower than other methods under comparison.

In this study, all analyses were performed in a high-throughput cluster-computing environment where each computing node had a shared memory of 48 GB. Sequencing data were split into individual chromosomes and chromosome-wise data were then analyzed in parallel on multiple computing nodes. Thus, the running time of a method is determined by the most time-consuming chromosome. Given read-depth data from WGS, AS-GENSENG can call CNVs for a sample with ∼30× coverage within 2 h, while ERDS and CNVnator in <1 h. For normalized read-depth data from WES, all three competing methods (AS-GENSENG, XHMM and Conifer) can call CNVs within 1 h for 300 samples and 200K exon targets.

In sum, we have developed a novel method AS-GENSENG with the following distinguishing features: (i) joint analysis of both TReC and ASReC while accounting for various experimental biases in sequence data, (ii) ability to detect both CNVs and ASCNVs from both WGS data and WES data and (iii) ability to leverage ASReC and large-scale nature of WES projects for effective data normalization and accurate detection of common CNVs with various frequencies. Through rigorous assessment using simulation, empirical data and independent technology, we have demonstrated the superior performance of AS-GENSENG in numerous examples. We conclude that AS-GENSENG not only predicts accurate allele-specific CNV calls but also improves the accuracy of total copy number calls.

## AVAILABILITY

The AS-GENSENG software and source code are freely available at https://sourceforge.net/projects/asgenseng.

## WEB RESOURCES

- The 1000GP alignment files: ftp://ftp-trace.ncbi.nih.gov/1000genomes/ftp/data/.
- SNP genotypes from the HapMap project: http://hapmap.ncbi.nlm.nih.gov/downloads/phasing/2009--02_phaseIII/HapMap3_r2/CEU/TRIOS/.
- The R/asSeq package: http://www.bios.unc.edu/∼weisun/software/asSeq.htm.
- The 1000GP released deletion set: ftp://ftp.broadinstitute.org/pub/svtoolkit/misc/1kg/NGPaper/.
- The Coriell repository: http://ccr.coriell.org.
- The R/ExomeDepth package: http://cran.r-project.org/web/packages/ExomeDepth/vignettes/ExomeDepth-vignette.pdf.
- The Genome STRiP FAQ: http://gatkforums.broadinstitute.org/discussion/1490/frequently-asked-questions.

## SUPPLEMENTARY DATA

Supplementary Data are available at NAR Online.

SUPPLEMENTARY DATA
