# Supplementary material for: Allele-specific copy-number discovery from whole-genome and whole-exome sequencing
Source: Nucleic Acids Res. 2015 Apr 16;43(14):e90. doi: 10.1093/nar/gkv319 (PMC4538801; doi:10.1093/nar/gkv319)
Supplement: SUPPLEMENTARY DATA [file supp_43_14_e90__index.html]

Allele-specific copy-number discovery from whole-genome and whole-exome sequencing — SUPPLEMENTARY DATA 

# Allele-specific copy-number discovery from whole-genome and whole-exome sequencing

## SUPPLEMENTARY DATA

**Files in this Data Supplement:**

- SUPPLEMENTARY DATA
