# Supplementary material for: Allele-specific copy-number discovery from whole-genome and whole-exome sequencing
Source: Nucleic Acids Res. 2015 Apr 16;43(14):e90. doi: 10.1093/nar/gkv319 (PMC4538801; doi:10.1093/nar/gkv319)
Supplement: SUPPLEMENTARY DATA [file supp_gkv319_nar-00589-met-n-2015-File006.pdf]

Supplementary Methods for "Allele-specific copy-number discovery from whole-genome and whole-exome sequencing"

WeiBo Wang, Wei Wang, Wei Sun, James J. Crowley, and Jin Szatkiewicz

Contents

Supplementary Methods 3

1 Data preparation 3

1.1 Sequencing reads . . . 3

1.1.1 Whole-genome sequencing data and alignment . . . 3

1.1.2 Whole-exome sequencing data and alignment . . . 4

1.1.3 Targets definition . . . 4

1.1.4 Alignment tool and reference . . . 4

1.1.5 Read quality control . . . 4

1.1.6 Heterozygous SNPs . . . 4

1.1.7 Allele-specific alignments . . . 5

1.2 Selection of genomic regions to be studied . . . 5

1.2.1 Window in WGS data, and selection of window size . . . 5

1.2.2 Exon capturing targets in WES data . . . 5

1.3 Read depth and covriates generation . . . 6

1.3.1 Counting method . . . 6

1.3.2 Counting for the WGS data . . . 6

1.3.3 Counting for the WES data . . . 6

1.3.4 Computation of the covariates . . . 6

2 Hidden Markov Model 7

2.1 Hidden state . . . 8

2.2 Transition probability . . . 8

2.3 EmissionProbability . . . 8

2.3.1 Likelihood of ASReC . . . 9

2.3.2 Likelihood of TReC . . . 10

2.3.3 Expected TReC for WGS data . . . 10

2.3.4 Expected TReC for WES data . . . 10

2.3.5 Overall emission probability . . . 12

2.4 Calling AS-CNV . . . 12

2.5 Training . . . 13

2.5.1 Transition probability and Initial probability . . . 13

2.5.2 E-step . . . 13
2.5.3 M-step . . . 14
2.5.4 Autoregressive HMM . . . 15
2.6 Post-processing . . . 16

3 Experiment **16**
3.1 WGS simulation . . . 16
3.2 WES simulation . . . 17

4 Reference **18**

List of Figures

S1 Example of ASCNV in WGS simulation data . . . 20
S2 Example of ASCNV in WGS simulation data . . . 21
S3 Detected high-confidence deletion after incorporating AS information in WGS data . 22
S4 Detected high-confidence deletion after incorporating AS information in WGS data . 23
S5 Detected high-confidence deletion after incorporating AS information in WGS data . 24
S6 Detected high-confidence deletion after incorporating AS information in WGS data . 25
S7 Detected high-confidence deletion after incorporating AS information in WGS data . 26
S8 Detected high-confidence deletion after incorporating AS information in WGS data . 27
S9 Detected high-confidence deletion after incorporating AS information in WGS data . 28
S10 Detected high-confidence deletion after incorporating AS information in WGS data . 29
S11 Detected high-confidence deletion after incorporating AS information in WGS data . 30
S12 Detected high-confidence deletion after incorporating AS information in WGS data . 31
S13 Detected high-confidence deletion after incorporating AS information in WGS data . 32
S14 Detected high-confidence deletion after incorporating AS information in WGS data . 33
S15 Detected high-confidence deletion after incorporating AS information in WGS data . 34
S16 HMM flowchart to infer allele-specific CNV . . . 35
S17 Illustration of sensitivity and FDR calculation . . . 36
S18 Whole-exome sequencing-simulation flowchart . . . 37
S19 Better estimation of TReC for WES after incorporating AS information . . . 38
S20 Better estimation of TReC for WES after incorporating AS information . . . 38
S21 Better estimation of TReC for WES after incorporating AS information . . . 39
S22 Better estimation of TReC for WES after incorporating AS information . . . 39
S23 Better estimation of TReC for WES after incorporating AS information . . . 40
S24 Better estimation of TReC for WES after incorporating AS information . . . 40
S25 Better estimation of TReC for WES after incorporating AS information . . . 41
S26 Better estimation of TReC for WES after incorporating AS information . . . 41
S27 Better estimation of TReC for WES after incorporating AS information . . . 42
S28 Better estimation of TReC for WES after incorporating AS information . . . 42
S29 Better estimation of TReC for WES after incorporating AS information . . . 43

List of Tables

S1 Notations 44
S2 allelic configurations and weights for each state 45
S3 beta-binomial parameters of allelic configurations of states 45
S4 Methodologies comparisons among WGS methods 46
S5 Methodologies comparisons among WES methods 47
S6 Performance comparison on WGS-read-count-simulation data 48
S7 Performance comparison on WGS-sequencing-read-simulation data 49
S8 Performance comparison with WGS methods on simulation data 50
S9 Performance comparison on WGS simulation down-sampled data 51
S10 Performance comparison on WGS empirical down-sampled data 52
S11 Performance comparison on WES-simulation data 53
S12 Performance comparison with WES methods on WES-simulation data 54

Supplementary Methods

The supplementary methods section mainly consists of three parts, which are the data preparation, the Hidden Markov Model, and the experiment. In the data preparation, we provide details about the procedure of generating input data of the proposed CNV detection algorithm. Our algorithm is based on the hidden Markov model, and we mathematically characterize the topology of the HMM used in this work. Specifically, we focus on two contributions of this work. The first one is modeling the ASReCs, and incorporating them into the hidden Markov model emission probability. The second one is aggregating multiple individuals to estimate the expected TReCs of copy number 2 (referred as expected TReCs in the following discussion) for exome sequencing data, and adapting the properties of exome sequencing into the HMM. In the experiment, we mainly introduce the simulations setup in this work. For the ease of reference, please refer to Table S1 for the list of notations used in the supplementary methods. .

1 Data preparation

The data preparation section comprises 3 main steps.

- The procedure of obtaining of sequencing reads and generating allele-specific reads (AS reads).
- The procedure of selecting genomic regions to be studied.
- The procedure of generating read depth and covariates for each selected genomic region.

1.1 Sequencing reads

1.1.1 Whole-genome sequencing data and alignment

The two HapMap samples studied in this work are European ancestry (NA12891, NA12892), sequenced to $\sim$ 30X coverage on average using the Illumina Genome Analyzer (I and II) platform. Sequencing reads are a mixture of single-end and paired-end with varied reads lengths (36bp,

51bp). The genome sequence data are all downloaded in the form of ".bam" alignment files from `ftp://ftp-trace.ncbi.nih.gov/1000genomes/ftp/data/`. Details about the alignments could be found at `ftp://ftp-trace.ncbi.nih.gov/1000genomes/ftp/README.alignment_data`.

1.1.2 Whole-exome sequencing data and alignment

The whole-exome sequenced samples include 324 individuals from 4 different populations (European, Asian, American, and African) sequenced using different capture platforms, which were 'SeqCap EZ Human Library' (v1.0 and v2.0) from Nimblegen, and 'SureSelect All Exon V2 Target Enrichment' kit from Agilent. Sequencing reads are all paired-end, and the read length is the same within each sample (among the studied samples we find 3 different read length: 76bp, 90bp, and 100bp). The exome sequencing data are all downloaded in the form of ".bam" alignment files from `ftp://ftp-trace.ncbi.nih.gov/1000genomes/ftp/data/`. Details about the alignments could be found at `ftp://ftp-trace.ncbi.nih.gov/1000genomes/ftp/README.alignment_data`.

1.1.3 Targets definition

We downloaded the capturing target regions list of the whole-exome sequencing study from the 1000GP ftp site `ftp://ftp-trace.ncbi.nih.gov/1000genomes/ftp/technical/reference/exome_pull_down_targets/20130108.exome.targets.bed`. The list contained 193,637 exome capture targets and the targets spanned ~47Mbps. Details about the capturing targets could be found at (`ftp://ftp-trace.ncbi.nih.gov/1000genomes/ftp/technical/reference/exome_pull_down_targets/20130108.exome.targets.bed.README`).

1.1.4 Alignment tool and reference

Reads were aligned using BWA [6] (v0.5.5) by 1000GP, and the reference is the human reference genome NCBI37. Details about the alignment procedure could be found at `ftp://ftp.ncbi.nlm.nih.gov/1000genomes/ftp/README.alignment_data`. For sequencing reads with the coordinate in NCBI36, we use liftOver to convert them to NCBI37.

1.1.5 Read quality control

We did the following steps to do the quality control. 1. Remove any read that fails quality checks (in the Flag field), such as either a PCR duplicate or an optical duplicate. 2. Extract all single-end reads and properly paired paired-end reads (based on the Flag field information). 3. Extract confidently aligned reads with MAPQ $\geq$ a specified threshold. In this study, we use MAPQ $\geq$10, which is empirically determined.

1.1.6 Heterozygous SNPs

AS-GENSENG requires dense, phased genotypes to make proper allele specific CNV calls (alleles A and B must be consistent across different markers). SNP genotypes were first obtained from microarray or sequence-based SNP-calling algorithms, such as samtools [7] or GATK [9, 3, 11]. We then carried out imputation to obtain a phased and dense list of input SNPs. For the two studied WGS samples, we used MaCH-Admix [8] to impute from the relatively sparse HapMap3 r2 SNPs list (obtained from microarray `http://hapmap.ncbi.nlm.nih.gov/downloads/phasing/`

2009-02_phaseIII/HapMap3_r2/CEU/TRIOS/) to generate a dense-phased SNP list. The imputation for the reference SNP panel was 1000 GP-released PhaseI V3 haplotypes on 1092 samples (phased, downloaded from ftp://share.sph.umich.edu/1000genomes/fullProject/2012.03.14/phase1_release_v3.20101123.snps_indels_svs.genotypes.refpanel.ALL.vcf.gz.tgz). The SNP coordinates reported in HapMap3 r2 were translated from NCBI36 to NCBI37 using liftOver; the reference panel was NCBI37. After imputation, we extracted 198k and 200k phased heterozygous SNPs from samples NA12891 and NA12892, respectively. For the 324 WES samples, we used an in-house python script to extract phased heterozygous SNPs from the reference panel (on average, 2.2 million heterozygous SNPs per sample). The coordinates were NCBI37.

1.1.7 Allele-specific alignments

Allele-specific alignments (AS alignments) are aligned reads that could be confidently assigned to one particular SNP allele. We used the function extractAsReads in R/asSeq (http://www.bios.unc.edu/~weisun/software/asSeq.htm) to extract AS alignments passing QC from alignment files. For each aligned read, the asSeq package searched the heterozygous SNP list by coordinates and counted the number of SNPs from alleles A or B that the read carried. If the count of allele A was >0, asSeq output the read to the A allele file, and if the count of allele B is >0, asSeq output it to the B allele file. If both counts were >0, the read was treated as an error, because each read is expected to carry only one allele. Such reads were discarded, but they were rare (<0.1%) in our experiment.

1.2 Selection of genomic regions to be studied

1.2.1 Window in WGS data, and selection of window size

In this study, we used 500bp-sliding windows with a step size of 100bp and this choice was first determined via simulation and then verified empirically. In our simulation study, we first simulated sequencing reads from a hypothetical CNV-containing genome (see Section 3.1 for detail), and then computed four sets of read-depth data for various sizes of sliding windows: 200bps, 300bps, 400bps and 500bps (each with a step size of 100bp). The best detection performance was achieved for 500bp-windows (93% sensitivity and 4% FDR). When 200bp-windows were used, we observed slightly improved sensitivity (94%) but much higher FDR (9%). This result was verified by empirical experiments, where we applied AS-GENSENG to 1000GP WGS data using both 500bp- and 200bp-sliding windows. Results for 500bp-windows are reported in Table 2 of the main paper. When 200bp-windows were used, we observed higher sensitivity (∼3% higher for each HapMap sample analyzed in this study), but much reduced specificity (>20% more predicted CNV calls). Therefore, both simulation and real-data analysis confirmed the choice of 500bp-sliding windows used in this study, presumably because it resulted in better signal-to-noise ratios in comparison to smaller size windows.

1.2.2 Exon capturing targets in WES data

We excluded a small proportion of targets. We first removed targets have too small TReC value (mean TReC < 1). We then removed targets that are in complex genomic regions (determined by our mappability scores, see below) and thus the TReC signal in these signals are not reliable.

1.3 Read depth and covriates generation

The input data is a sequence of two-tuples for each studied genomic region (window, or target) represent by
$\{O, X\} = \{o_1......o_T, x_1......x_T\}$, where T is the total number of genomic regions studied of a chromosome. $o_t = (o_t^{all}, o_t^{(A)}, o_t^{as})$ denotes the TReC, ASReC from allele A, and ASReC from both alleles of the tth genomic region. $x_t = (g_t, l_t)$ denotes the covariates of the tth genomic region, where g_t represents the GC content, and l_t denotes the mappability score. We will first introduce the counting method for generating TReC and ASReC, and then introduce the method to compute the covariate values.

1.3.1 Counting method

When counting, we ensured that each fragment is counted only once. The studied WGS sequencing data are a mixture of single end read and paired ends read. Thus we counted them separately. To properly handle with WES sequencing reads which may span multiple targets, we characterized the corresponding DNA fragment of each paired ends read as the leftmost aligned position and the rightmost aligned position of the two ends, and count on the basis of such fragment. Below we give the details.

1.3.2 Counting for the WGS data

The following method could be used to count both TReC and ASReC of the WGS data. If the input are alignments of all reads, it will generate TReC, while if the input are allele-specific alignments (AS alignments), it will generate ASReC.

1. If two ends of a pair fall in two windows, assign 1/2 to each window where the ends fall;
2. If both ends of a pair fall in the same window, assign 1 to the window;
3. If paired-ended but only one-end present, assign 1/2 to the window where the ends fall;
4. If single-end, always assign 1 to the window where the end falls.

1.3.3 Counting for the WES data

The following method could be used to count both TReC and ASReC of the WES data. If the input are alignments of all reads, it will generate TReC, while if the input are AS alignments, it will generate ASReC.

1. Characterize the fragment as the left-most aligned position and the right-most aligned position of the two paired ends.

2. Check the quality of the characterized fragment. Remove if it looks abnormal, e.g. the length of its spanned region is much larger than the library insert size.

3. Intersect the spanned region with the targets list. Assign 1 to the overlapped target if it only intersects with one target. Assign $1/N$ to overlapped targets if it intersects with N targets.

1.3.4 Computation of the covariates

We computed two covariates, which are GC content and mappability scores, for each studied genomic region to quantify the known bias in order to proper adjust the caused effects on the read depth. The reference we used to generate the covariates are from 1000GP (`ftp://ftp-trace.ncbi.nih.gov/1000genomes/ftp/technical/reference/README.human_g1k_v37.fasta.txt`).

GC content is computed using the following steps. (1) Calculate the proportion of G or C bases in each studied genomic region from a given reference genome.(2) Apply a cubic spline smoothing and then transform the GC proportion based on the fitted curve so that the transformed GC proportion and logarithm of the read count are linearly correlated. (3) The transformed GC proportion is median-centered and is referred to as GC content hereafter.

Mappability score is computed using the following steps. (1) Align the K-mers starting at each base position back to reference genome using a desired aligner, e.g. BWA (Li and Durbin 2009). (2) Identify base positions where the corresponding K-mers are correctly aligned (i.e. there is a unique best hit and it is the true position of the K-mer). (3) Compute mappability score as the proportion of correctly-aligned bases (a.k.a. mappable bases) in a given genomic region.

2 Hidden Markov Model

The hidden Markov model (HMM) used in this work is a time-homogeneous discrete HMM. We use it to segment the genome to regions of same copy number. Below we first give a summary about the mathematic characterization of the HMM used in this study, and then introduce the settings of each component of HMM structure in detail.

In our HMM, time represents the studied genomic regions, denoted by t.

The state represents the underlying copy number (CN). The state variable $q_t = CN_t$ is hidden and discrete with N possible values, $(0, 1, ..., N-1)$, where N is derived from the data by K-mean clustering the logarithm of the read count. For more precise modeling in this study, we assume seven hidden states representing copy numbers of 0, 1, 2, 3, 4, 5, and 6 or more. Among the 7 states, state 2 represents the copy number neural (2). Our modeling can be extended to arbitrary positive integer hidden state N. A particular sequence of the states is described by $q = (q_1, ..., q_T)$, where T is the total number of studied genomic regions in a chromosome. Let π_j be the initial probability of state j, which is the probability of the state of the first window being state j.

The underlying hidden Markov chain is defined by state transitions $P(q_t|q_{t-1})$ and is represented by a time-independent stochastic transition matrix $A = \{a_{jz}\} = P(q_t = z|q_{t-1} = j)$.The transitions in this work is modeled as a first-order time-homogeneous Markov process (the state in one genomic region is only affected by its immediately previous region and will only affect its immediately next region), i.e. $P(q_t = z|q_{t-1} = j) = P(q_t = z|q_{t-1} = j, q_{t-2} =, ..., q_1 =)$. For WES data, due to the sparse nature of exome capturing, the distance between adjacent targets may be extreme large. To model it properly, we add a distance-dependent exponential attenuation factor to the state transition matrix $A = \{f(a_{jz})\}$. We will introduce how f is setting for different states in the coming sections.

Each copy number state emits an observation, the read count. In this work, O_t, consists of three discrete count variable. One is the count of all reads (o_t^{all}), the other two are the count of all AS reads, o_t^{as} and the AS reads coming from allele A, $o_t^{(A)}$. We denoted the emission probability of a particular observation at a particular time t for state j as $e(t, j) = Pr(o_t = (o_t^{all}, o_t^{(A)}, o_t^{as})|q_t = j, x_t)$. For a detailed description of the emission probability, see the following Section.

We use the Baum-Welch algorithm [12] to find the maximum likelihood estimates (MLE) of the HMM parameters. Following Bilmes [1], we define the complete-date likelihood and solve the Q function in order to find the maximum likelihood estimates (MLE) of the HMM parameters.

A standard HMM assumes the Markov property, $P(q_t|q_{t-1}, q_{t-2}, q_{t-3}, ...q_1) = P(q_t|q_{t-1})$. An additional assumption that is often employed is that the observations are independent given the

states, $O_t \perp O_i(i \neq t)|q_t$, which is valid when the genomic regions are non-overlapping. When the genomic regions are overlapping (in our WGS, windows are overlapping), this assumption is invalid; and instead, the observations are drawn from an autoregression process [5]. We have implemented an autoregressive HMM to model this feature of the data.

2.1 Hidden state

Each hidden state represents a copy number. In this work, we assume seven hidden states representing copy numbers of 0, 1, 2, 3, 4, 5, and 6 or more according to practical concerns (computation and data availability). We did not model allele-specific configuration as states in HMM because of two reasons. The first reason is that not every genomic region has AS reads. The second reason is that the allele-specific state of one genomic region does not necessary depend with its adjacent genomic region.

2.2 Transition probability

The setting of transition probability matrix $A = \{a_{jz}\}$ follows two intuitions. First the genomic regions near by tend to be in the same state. As a result, the self-transition probability a_{jj} will be much larger among the transition probability transiting from state j to other states. Its effect is that the adjacent genomic regions will more likely be in the state. The second intuition is that most genomic region should be in copy number neural region. Its implications are two folds. First is that the self transition probability of the normal state (2 in this work) is higher than other states, i.e., genomic regions are more likely in the copy number neural regions. Second is that the probability transiting to copy number neural state is higher than transiting to other states.

Due to the sparse capturing property of exome capture, we add a distance-dependent exponential attenuation factor [4] to $\{a_{jz}\}$. The intuition is that two targets far away are not necessary to have the same copy number, i.e. when the distance to the previous targets is extremely larger, the copy number information of previous target is not as much useful for inferring the state of the current target as when the distance is small. For targets far away, the probability transiting from previous state is similar to transiting from normal copy number to the current state. We use $e^{\frac{-d}{D}}$ to measure if two targets are extreme far away relative to the average, where d is the distance of the current target, and D is the average distance for all targets. We combine the intuition into the following formula $a'_{jz} = e^{\frac{-d}{D}} \times a_{jz} + (1 - e^{\frac{-d}{D}}) \times a_{2z}$. When the distance is relative large, $e^{\frac{-d}{D}}$ is close to 0, and a_{2z} is the dominant factor, and vice versa. In this work, state 2 represents the copy number neural.

2.3 EmissionProbability

Each copy number state emits an observation, the read count. However, the underline copy number is un-observed, a.k.a, hidden states. In this work, we calculate the likelihoods of observing the TReC signal, including ASReC, for each possible underline copy number. The likelihoods are incorporated as the emission probability. We model the likelihood of observing the TReC and ASReC as $Pr(o_t = (o_t^{all}, o_t^{(A)}, o_t^{as})|q_t = j, x_t)$, where $o_t^{all}, o_t^{(A)}, o_t^{as})$ are the TReC and ASReC, q_t is the underline hidden copy number, and x_t is the covariates at this genomic region. The likelihood could be divided into three factors,

$$Pr(o_t = (o_t^{all}, o_t^{(A)}, o_t^{as})|q_t = j, x_t) = Pr(o_t^{all}|q_t = j, x_t)Pr(o_t^{as}|q_t = j, o_t^{all}, x_t)Pr(o_t^{(A)}|o_t^{as}, o_t^{all}, q_t = j, x_t).$$

$Pr(o_t^{all}|q_t = j, x_t)$ is the likelihood of observing TReC o_t^{all} for state j. It could be modeled as a negative binomial distribution, we denote it as $\ell(o_t^{all}|j) = Pr(o_t^{all}|q_t = j, x_t)$, and will discuss it in detail later. Now let's look at the second factor $Pr(o_t^{as}|q_t = j, o_t^{all}, x_t)$. Given TReC o_t^{all}, the ASReC o_t^{as} is conditional independent of the state j and the covariates x_t. Thus $Pr(o_t^{as}|q_t = j, o_t^{all}, x_t) = Pr(o_t^{as}|o_t^{all})$. $Pr(o_t^{as}|o_t^{all})$ depends on the number of heterozygous SNPs in the tth genomic region. In this study we modeled it as a constant. Finally let's look at the third factor $Pr(o_t^{(A)}|o_t^{as}, o_t^{all}, q_t = j, x_t)$, which is the likelihood of observing ASReC and distribution of ASReC from one of the two alleles. Given the particular state j and ASReC o^{as}, the ASReC from allele A $o^{(A)}$ is conditional independent of TReC o_t^{all}, and the covariates x_t. Thus $P(o_t^{(A)}|o_t^{as}, o_t^{all}, q_t = j, x_t) = P(o_t^{(A)}|o_t^{as}, q_t = j)$. We modeled the allele-specific likelihood (AS likelihood) as a beta-binomial distribution, we denote it as $\ell(o_t^{(A)}|o_t^{as}, j) = P(o_t^{(A)}|o_t^{as}, q_t = j)$, and will discuss it in detail later. Thus the overall likelihood $\ell(o_t|t) = \ell(o_t^{all}|j) * \ell(o_t^{(A)}|o_t^{as}, j)$ is the product of likelihood of TReC and likelihood of ASReC. To further model the background noise, we model the emission probability as a mixture of a uniform distribution and the TReC likelihood.

$$e(t, j) = c/R_m + (1 - c)\ell(o_t|t) \quad (1)$$

where c is the proportion of the random uniform component and is fixed as constant for each state; and R_m is the largest read count among all genomic regions and thus $1/R_m$ is the uniform density.

2.3.1 Likelihood of ASReC

We first introduce the likelihood calculation of ASReC as it is the same for both WGS data and WES data. Each particular copy number has different allelic configurations. For example, one copy deletion has two allelic configurations, A or B. For each possible allelic configuration, we could model its likelihood following a beta-binomial distribution $\varphi(o^{(A)}, o_t^{as}, \pi, \theta)$. π is the expected proportion of AS reads from allele A, e.g.,π equals 0.33 for allelic configuration ABB. θ is a dispersion parameter. If there is no over-dispersion, then φ degenerates to binomial distribution. The likelihood of a state is its weighted average sum of likelihoods of all its possible allelic configurations. We listed all possible allelic configurations of each state of the 7 states setting in this work, and corresponding weights in Table SS2. Note that we assume no complex CNV in this study, i.e., no two CNV happen at the same locus. Thus ABB and AAB are the only two possible allelic configurations for one copy duplication (state 3). AAA and BBB are not studied, as they indicate one copy deletion and three copy duplication happened at the same locus. We give the likelihood for each of the 7 states setting used in this work below:

- if $j = 0$,$\ell(o_t^{(A)}|o_t^{as}, j)= w_{0,0}\varphi(o^{(A)}, o_t^{as}, p_{0,0}, \theta)$.
- if $j = 1$, $\ell(o_t^{(A)}|o_t^{as}, j)=w_{1,0}\varphi(o^{(A)}, o_t^{as}, p_{1,0}, \theta)+w_{1,1}\varphi(o^{(A)}, o_t^{as}, p_{1,1}, \theta)$.
- if $j = 2$, $\ell(o_t^{(A)}|o_t^{as}, j)=w_{2,0}\varphi(o^{(A)}, o_t^{as}, p_{2,0}, \theta)$.
- if $j = 3$, $\ell(o_t^{(A)}|o_t^{as}, j)=w_{3,0}\varphi(o^{(A)}, o_t^{as}, p_{3,0}, \theta) + w_{3,1}\varphi(o^{(A)}, o_t^{as}, p_{3,2}, \theta)$.

- if $j=4$, $\ell(o_t^{(A)}|o_t^{as},j)$=$w_{4,0}\varphi(o^{(A)},o_t^{as},p_{4,0},\theta)+w_{4,1}\varphi(o^{(A)},o_t^{as},p_{4,1},\theta)+w_{4,2}\varphi(o^{(A)},o_t^{as},p_{4,2},\theta)$.
- if $j=5$, $\ell(o_t^{(A)}|o_t^{as},j)$=$w_{5,0}\varphi(o^{(A)},o_t^{as},p_{5,0},\theta)+w_{5,1}\varphi(o^{(A)},o_t^{as},p_{5,1},\theta)+w_{5,2}\varphi(o^{(A)},o_t^{as},p_{5,2},\theta)$ $+\ w_{5,3}\varphi(o^{(A)},o_t^{as},p_{5,3},\theta)$.
- if $j=6+$, $\ell(o_t^{(A)}|o_t^{as},j)$=$w_{6,0}\varphi(o^{(A)},o_t^{as},p_{6,0},\theta)+w_{6,1}\varphi(o^{(A)},o_t^{as},p_{6,1},\theta)+w_{6,2}\varphi(o^{(A)},o_t^{as},p_{6,2},\theta)$ $+\ w_{6,3}\varphi(o^{(A)},o_t^{as},p_{6,3},\theta)$+$w_{6,4}\varphi(o^{(A)},o_t^{as},p_{6,4},\theta)$.

where φ is the beta-binomial distribution. $p_{j,h}$ is the expected proportion of AS reads from allele A. Please refer to Table SS3 for the values of $p_{j,h}$ using j and h as reference. $w_{j,h}$ are the weights of all allelic configurations of state j. Please refer to Table SS2 for the values of each weight and the corresponding allelic configurations. θ is the over-dispersion parameter of the beta-binomial distribution. We set it as 0.1 from empirical study.

2.3.2 Likelihood of TReC

We assume that TReC follows negative binomial distribution. It provides a over-dispersion parameter to accommodate the larger variance comparing with poisson distribution. We have shown in our early work (GENSENG) that the assumption holds for the TReC data. We need to obtain the expected TReC μ_j for each possible underline copy number j (j is the index of the copy number state, or CN_t), and calculate the likelihood. In this work, we apply two approaches to compute μ_j for WGS data and WES data separately.

2.3.3 Expected TReC for WGS data

We use the linear regression formula to estimate the expected value from the underline copy number.

$$\log(\mu_{tj}) \quad = \quad \beta_0 + \beta_1 * \log(CN_t) + \beta_2 * \log(l_t) + \beta_3 * \log(g_t) \tag{2}$$

where t denotes the tth window, j emphasizes the dependency of the expected μ_t on the copy number CN_t. In addition to the dependency between expected TReC and copy number, we also find that in our studied WGS data, expected TReC is affected by the corresponding covariates(l_t is the mappability score, g_t is the GC content). The effect the covariates could be appropriately accommodated in this formula. For computational convenience, we set $CN_t = 0.5$ when $j = 0$, and set $CN_t = j$ when $j > 0$. Also it is quite convent to incorporate new covariates into this formula. We employ a log link function to acknowledge the fact that $\mu_{tj} > 0$. $\beta_0, \beta_1, \beta_2, \beta_3$ are the regression coefficients. Specifically, $\beta_0 = \log(\alpha_0)$, is the intercept parameter and is interpreted as the average level of read count signal when all covariates are equal to zero. β_1 is the amount of increase of read count for every unit increase of copy number, CN. β_2 is the amount of increase of read count for every unit increase of the mappability score, l. β_3 is the amount of increase of read count for every unit increase of the GC content, g.

2.3.4 Expected TReC for WES data

We use data across windows to fit the equation 2 assuming copy number having the same effect across different windows. This assumption does not hold for WES data due to the non-uniform TReC signal between targets. Thus we apply a different approach, which aggregated multiple

samples of one target to estimate the expected TReC. In the estimation, we assume the ratio between the TReC and the sum of TReCs of all targets is consistent between samples. Thus we use the median ratio as the expected ratio, and obtain the expected TReC through multiplying the expected ratio with the sum of TReCs of all targets in the sample.

$$\mu_{k,t} = d_k * e^{median_{1\leq k\leq N}(log(\frac{o_{k,t}}{d_k}))}.$$

where $\mu_{k,t}$ is the expected TReC of sample k at target t, $o_{k,t}$ is the TReC of sample k at target t, d_k is the sum of TReCs of all targets of sample k. Divergent from the expected value indicates the existence of CNV. We collect the difference with the expected TReC from the targets of one sample, and estimate the underline copy number. However, when facing common CNV, the ratios of samples affected by the common CNV will be remarkably different from the ratios of samples having copy number 2. The median ratio of all samples will be in between. The median of all samples does not longer provide a baseline for samples having copy number 2. In order to obtain such a baseline, we develop the following method, which use the AS information to find a group of samples potential having copy number 2.

- Find the number of clusters of samples data using the boot.comp function from R/mixtools package. We pass all ratios of samples in as the data, set the max.comp parameter as 3, set the B parameter is set as 5, and set the mix.type parameter as "normalmix". Denote the found number of samples as K.

- Cluster the samples using the normalmixEM function from R/mixtools package. We pass all ratios of samples in as the data, and set the k parameter as K.

- Use AS information to find the cluster of samples potential having copy number 2. For each sample in one cluster, we calculate its likelihood being state j using $\ell(o_t^{(A)}|o_t^{as}, j)$, if it is allele-specific informative (o^{as} is greater than a given threshold, 10 in this study, denote as AS informative in the following discussion). Then we calculate the likelihood of a cluster being state j by multiplying the likelihood of all AS informative samples in the cluster. We finally calculate the posterior probability of a cluster having copy number 2 through dividing the likelihood of the cluster being state 2 by the sum of likelihood of the cluster being each state. We select the cluster with the largest posterior probability being state 2 as the cluster containing no CNV individuals. Denote the cluster as G, which contains the index of samples, and its cardinality as N', the expected value is calculated as

  $$\mu_{k,t} = d_k * e^{median_{1\leq k\leq N'}(log(\frac{o_{G(k),t}}{d_{G_k}}))}.$$

  Note that we do not need to know whether there is a common CNV. If there is no common CNV, most of samples will be clustered in one group, and the median ratio of this group is still a good baseline for samples having copy number 2.

We then employ a log link function to acknowledge the fact that $\mu_{k,t} > 0$, and obtain

$$log(\mu_{k,t}) = log(d_k) + median_{1\leq k\leq N'}(log(\frac{o_{G(k),t}}{d_k})) + \beta_1 log(CN_{k,t}/2). \quad (3)$$

β_1 is the regression coefficient. It is the amount of increase of read count for every unit increase of copy number, $CN_{k,t}$. In this work we removed exome targets which are strongly affected by

covariates (i.e. extreme low mappability). Thus we do not add covariates to equation 3. However, any covariates could be easily added into the equation.

Not every target has AS informative data available, as the ASReC may not be large enough. When not available, we develop a comparable targets method to find samples having copy number 2. The assumption is that it is copy number 2 in most targets. We use the TReC ($o_{k,t}$) from other targets with the same target length and similar covariates value as the baseline, and infer that the group with mean TReC closet to the baseline as the group of samples having copy number 2.

- Cluster the samples using the same method.
- Calculate the mean TReC of each clusters. Denote as $mean(G_1), mean(G_2), ..., mean(G_K)$.
- Find targets with the close target length and covariates as the tth target. Denote these targets as comparable targets. For each comparable target, calculate the mean TReC of all individuals and use it as the representative value of the target. Calculate the mean value of the representative value of the comparable targets. Denote it as C.
- Select the cluster which is $\text{argmin}_k |C - mean(G_k)|$ as the cluster of samples having copy number 2.

2.3.5 Overall emission probability

Now we multiply the likelihood of the TReC signal with the likelihood of the ASReC signal, combine the product with a uniform distribution to form a mixture distribution, and finally obtain the overall emission probability equation.

$$e(t,j) = c/R_m + (1-c)\frac{\Gamma(o_t^{all} + 1/\phi_j)}{o_t^{all}!\Gamma(1/\phi_j)} \left(\frac{1}{1+\phi_j \mu_{tj}}\right)^{1/\phi_j} \left(\frac{\phi_j \mu_{tj}}{1+\phi_j \mu_{tj}}\right)^{o_t^{all}} * \sum_{h=0}^{||w_{j,.}||} (w_{j,h}\varphi(o^{(A)}, o_t^{as}, p_{j,h}, \theta)), \quad (4)$$

here c is the proportion of the random uniform component and is fixed as constant for each state; and R_m is the largest read count (o_t^{all}) among all windows and thus $1/R_m$ is the uniform density, ϕ_j is over-dispersion of the negative binomial distribution for state j, and μ_{tj} is the estimated TReC (o_t^{all}) of state j at the tth genomic region (window for WGS data, target for WES data), $||w_{j,.}||$ is the number of the allelic configurations of state j, φ is the beta-binomial distribution, $p_{j,h}$ is the expected proportion of AS reads from allele A, $w_{j,h}$ are the weights of all allelic configurations of state j, and θ is the over-dispersion for beta-binomial distribution.

2.4 Calling AS-CNV

We segment the genomic regions based on their most likely underline copy number. We use the posterior probability assigned to each possible underline copy number at each genomic region by HMM to measure the likelihood. The change of copy number reveal the breakpoints of CNV. Before the final CNV, we remove small CNV calls and merge nearby CNV calls with the same type to do regular post processing. After the regular post processing, we call CNV as segments most likely not having copy number 2, and we assign the most likely copy number in each segment as the inferred copy number to the segment. Then for AS informative CNV (ASReC larger than a given threshold, 10 in this study), we assign the most likely allelic configuration to the CNV. If the assigned copy number is j, we will calculate the beta-binomial likelihood $\phi(o^{(A)}, o^{as}, \pi, \theta)$ for each possible allelic

configuration of j, using the mean $o^{(A)}$ of the CNV as observation, the mean o^{as} of the CNV as the number of trials. π will be the expected proportion of AS reads from allele A of the corresponding allelic configuration (i.e. $\pi = 0.33$ for ABB). We select the allelic configuration with the largest likelihood as the allelic configuration of the CNV.

2.5 Training

It is essential a optimization problem to train the HMM parameters. To save computational time, we use the empirically determined transition probability, initial probability. We optimize the parameters in the emission probability. More specifically, we estimate the mean value μ (a.k.a, expected TReC), and the over-dispersion parameter in negative-binomial distribution. We use the standard Baum-Welsh algorithm to optimize the hidden states parameter in the HMM, which involves calculating the forward-backward probability. The optimization procedure is the same as the one developed in our early work (GENSENG). In the following, we give a concise version for the sake of self-content. Please refer to GENSENG for details. We will first introduce the empirically determined transition and initial probability. In the E-step of the Baum-Welsh algorithm, we will give the formula of forward and backward function. In the M-step of the Baum-Welsh algorithm, we will give the outline of the emission probability estimation algorithms. Finally, we will introduce the autoregressive HMM as the windows setting in our WGS allows overlapping windows.

2.5.1 Transition probability and Initial probability

(a) The number of states:

N is found from the data by finding the number of k-mean clusters of $\log(O)$. Here we assume N=7, for CN = 0,1,2,3,4,5,6+.

(b) Initial state probability, π_j:

For state CN = 2: 0.9995; for other states: (1-0.9995)/(N-1).

(c) Initial state transition probability, a_{jz}:

Self-transition probability: for state CN = 2: 0.9995; for other states: 0.995;

Transition probability to other states, i.e. a_{jz} when $z \neq j$:

Transiting from CN = 2 to $CN < 2$: (1-0.9995)/3; from CN = 2 to $CN > 2$: (1-0.9995)/12;

Transiting from $CN < 2$ to CN = 2: (1-0.995)/9; from $CN < 2$ to $CN < 2$: (1-0.995)/90; from $CN < 2$ to $CN > 2$: (1-0.995)/400;

Transiting from $CN > 2$ to $CN < 2$: (1-0.995)/40; from $CN > 2$ to CN = 2: (1-0.995)/1.25; from $CN > 2$ to $CN > 2$: (1-0.995)/20;

When analyze WES data, append distance-dependent exponential attenuation factor to $\{a_{jz}\}$, $\{a_{jz}\} = e^{\frac{-d}{D}} * \{a_{jz}\} + (1 - e^{\frac{-d}{D}}) * \{a_{2z}\}$, where d is the distance of the current target, and D is the average distance for all targets.

2.5.2 E-step

Forward-function is defined as $f(t, j) = P(o_1, o_2, ...o_t, q_t = j \texttt{ ends at } t|\Lambda_0)$, which Λ_0 are the current parameters of HMM, including transition probability, initial probability, and emission probability. The forward function for the first window $t = 1$ is calculated as $f(1, j) = \pi_z e(1, j)$. And for the windows afterwards, the forward function is calculated as $f(t, j) = e(t, j) \sum_j f(t-1, j) a_t(z, j)$. We use the AS information combined overall emission probability Equation (4) to compute $e(t, j)$.

Backward-function is defined as $b(t,z) = P(o_{t+1}, o_{t+2}, ...o_T | q_t = z \texttt{ ends at } t | \Lambda_0)$. The backward function at the last window $t = T$ is calculated as $b(T, z) = 1$. And for windows before, the backward function is calculated as $b(t-1, z) = \sum_j \left[a_t(z,j)e(t,j)b(t,j)\right]$

Posterior probability The posterior probability could be defined as the product of forward-backward function $\gamma(t,j) = P(q_t = j | \mathbf{O}, \Lambda_0) = \frac{f(1,j)b(1,j)}{p(\mathbf{O}|\Lambda_0)}$

2.5.3 M-step

In the M-step, we use IRLS and Newton-Raphson method to estimate the parameters of the negative binomial distribution.

Estimation of expected TReC using the IRLS method

Step 1. We are optimizing the likelihood of negative binomial distribution with respect to the mean value μ_{tj}, where $t = 1...T$, $CN_t = q_t = 0...j..(N-1)$. The likelihood function of the negative binomial distribution is as follows, where Γ is the Gamma function, ϕ is the over-dispersion, and o_t is the observed TReC, or we refer as o_t^{all} to differentiate with the ASReC. p_{tj} is the weight of state j at window t, we insert the posterior probability of state j at window t as the weight.

$$Lm = \sum_{t=1}^{T}\sum_{j=0}^{N}\left[\log\left(\Gamma(o_t + 1/\phi)\right) - \left(\frac{1}{\phi} + o_t\right)\log(\frac{1}{\phi} + \mu_{tj}) + \log(o_t + 1.0) + o_t\log(\mu_{tj})\right] p_{tj} \quad (5)$$

Step 2. Fit a weighted Poisson regression model using the IRLS procedure.

Step 3. Perform a score test
The score test [2] is used to test whether the overdispersion parameter, ϕ, is significantly greater than 0. If the score test is significant, we

- Estimate ϕ using the Newton-Raphson method as described in the "Estimation of overdispersion using the Newton-Raphson method".
- Proceed to step 4

Step 4. Fit a weighted negative binomial regression model using the IRLS method.

Estimation of overdispersion using the Newton-Raphson method

Given $\log(\mu_{tj})$, we use the Newton-Raphson method to estimate the overdispersion parameter, ϕ. In this study, we estimate one overdispersion parameter ϕ jointly for all states, and set $\phi_j = \phi$ for $j = 0..N-1$.

The following weighted log-likelihood and its first, second derivatives are used in the Newton-Raphson method to estimate ϕ. The weighted log-likelihood is the same as Equation (5).

$$Lm = \sum_{t=1}^{T}\sum_{j=0}^{N}\left[\log\left(\Gamma(o_t + 1/\phi)\right) - \left(\frac{1}{\phi} + o_t\right)\log(\frac{1}{\phi} + \mu_{tj}) + \log(o_t + 1.0) + o_t\log(\mu_{tj})\right] p_{tj}$$

It is computationally slightly easier to estimate $\varphi = 1/\phi$. Then,

$$Lm = \sum_{t=1}^{T}\sum_{j=0}^{N} \left[\log\left(\Gamma(o_t+\varphi)\right) - (\varphi+o_t)\log(\varphi+\mu_{tj}) + \log(o_t+1.0) + o_t\log(\mu_{tj})\right] p_{tj}$$

Thus the score function is

$$Score(\varphi) = \frac{\partial Lm}{\partial \varphi} = \sum_{t=1}^{T}\sum_{j=0}^{N-1}\left[\Psi(o_t+\varphi) - \Psi(\varphi) - \frac{\varphi+o_t}{\varphi+\mu_{tj}} - \log(\varphi+\mu_{tj}) + 1 + \log(\varphi)\right] p_{tj}$$

where $\Psi(x) = \partial\log\Gamma(x)/\partial x$, the digamma function. The observed Fisher information is

$$Info(\varphi) = -\frac{\partial^2 Lm}{\partial \varphi^2} = \sum_{t=1}^{T}\sum_{j=0}^{N-1}\left[-\psi(o_t+\varphi) + \psi(\varphi) + \frac{\mu_{tj}-o_t}{(\varphi+\mu_{tj})^2} + \frac{1}{\varphi+\mu_{tj}} - \frac{1}{\varphi}\right] p_{tj}$$

where $\psi(x) = \partial^2\log\Gamma(x)/\partial x^2$, the trigamma function.

We use the Newton-Raphson method given the score function and the fisher information.
Initialize $\varphi = \frac{\sum_{t=1}^{T}\sum_{j=0}^{N-1} p_{tj}}{\sum_{t=1}^{T}\sum_{j=0}^{N-1} p_{tj}(o_t-\mu_{tj})^2}$
WHILE (ABS(Dev) > Toleration) {
  Dev = $\frac{Score(\varphi)}{Info(\varphi)}$
  φ = φ+Dev
}

2.5.4 Autoregressive HMM

When overlapping windows are used, the observed read count is drawn from an autoregressive process. We implemented an autoregressive HMM. Specifically, a residual term is included as an additional predictor in the negative binomial regression model assuming first order autoregression. Given the notations defined in Section 4.3.4, we obtain:

- $\log(\overline{\mu}_{tj})$: estimated using IRLS method. The number of covariate $M = 1$, $x = \{\texttt{cov}^1\}$, $\texttt{cov}^1 = \{\texttt{cov}^1_1, ..., \texttt{cov}^1_T\}$, $\texttt{cov}^1_t = \{g_t, ...g_t, .., g_t\}$ (g_t repeats for N times).
- $\log(\overline{\mu}'_{tj})$: estimated using IRLS method. The number of covariate $M = 2$, $x = \{\texttt{cov}^1, \texttt{cov}^2\}$, $\texttt{cov}^1 = \{\texttt{cov}^1_1, ..., \texttt{cov}^1_T\}$,$\texttt{cov}^1_1 = \{0, ..., 0\}$ (0 repeats for N times),$\texttt{cov}^1_t = \{\log(o_{t-1}) - \log(\mu_{t-1,0}), \log(o_{t-1}) - \log(\mu_{t-1,1}), ..., \log(o_{t-1}) - \log(\mu_{t-1,N-1})\}, 1 \le t \le T$,$\texttt{cov}^2 = \{\texttt{cov}^2_1, ..., \texttt{cov}^2_T\}$, $\texttt{cov}^2_t = \{g_t, ...g_t, .., g_t\}$ (g_t repeats for N times).

The fitting process is implemented as the following:
Step 1: Fit initial weighted GLM to obtain $\log(\overline{\mu}_{tj})$ as in Section 4.3.4.
Step 2: Compute residual $r_{t-1,j} = \log(o_{t-1}) - \log(\overline{\mu}_{t-1,j})$ for $t > 1$ and let $r_{1,j} = 0$.
Step 3: Refit weighted glm to obtain $\log(\overline{\mu}'_{tj})$ including r_{t-1} as covariate $\texttt{cov}^1$.
Step 4: Compute emission probability using $\log(\overline{\mu}'_{tj})$.
Step 5: Compute Forward, Backward, Posterior probability as before.

Step 6: Parameter update for transition probability as before.

Step 7: Refit weighted glm to obtain $\log(\overline{\mu}_{tj})$ as in Section 4.3.4 using updated posterior probability.

Step 8: Update $r_{t-1} = \log(o_{t-1}) - \log(\overline{\mu}_{t-1,j})$ for $t > 1$ and let $r_{1,j} = 0$. where $\log(\overline{\mu}_{tj})$ is from the Step7.

Step 9: Refit weighted glm to obtain $\log(\overline{\mu}'_{tj})$ including r_{t-1} as covariate $\texttt{cov}^1$, using updated posterior probability.

Step 10: Iterate steps 4-9 until E-M converges.

2.6 Post-processing

Following the discovery in [10], it is crucial to merge CNV calls and filter false positives. We applied the same procedure to merge CNV calls. In addition, we used a combination of read-depth accessible (RDA) filter and confidence score as used in [10] as the filter to remove low confidence calls (WGS, keep RDA<0.15 for state 0 deletion, RDA<0.75 for state 1 deletion, RDA>1.25 for duplication; WES, keep calls with confidence score>0.99).

3 Experiment

In this study, we relied on the simulations to calibrate the performance of our algorithm on ASCNVs mainly due to the lack of empirical ASCN data. We developed two different methods to simulate WGS data and WES data separately. Below we give the details.

3.1 WGS simulation

We conducted two WGS experiments. The first experiment simulates the TReC and ASReC, and the second simulates the sequencing reads. The procedure of the first experiment is described here. We first selected chromosome 1 from HapMap sample NA12891 as a template (which provides the TReC, the covariates, and the ASReC). We assigned copy number 2 to all windows. We then implanted 200 CNVs (around 60% deletions and 40% duplications, median size = 3,000 bps) by modifying the underlying copy number. We assigned a randomly generated copy number range of 3-6 for duplications and 0-1 for deletions. Based on the copy number and the covariates we generated TReC. We passed the covariate matrix (columns were the assigned copy number, mappability score, and GC content respectively; rows were each sliding window) and coefficient vector (all initialized as 1) to the garsim function from R/gsarima to simulate TReC for each window. We applied the NB distribution with the log-link function for the garsim model, where the autoregressive parameter was set to 0.6 (because we used overlapping windows); the zero-correction parameter was set to "zq1" and the inverse of the overdispersion parameter was set to 0.01. In the next step we simulated ASReC. We first simulated AS configuration for each CNV and then used Beta-binomial distribution to simulate ASReC (ASReC is 5% of TReC, and the proportion of reads coming from allele A is determined by AS configuration).

The procedure of the second experiment is described as follows. We mimicked the sequencing experiment by generating paired end reads from a hypothetical chromosome. To simulate reads, we first simulated one pair of chromosomes using human chromosome 1 as the template and implanted 200 CNVs by modifying the sequence (around 60% deletions and 40% duplications, median size = 3000bps). We specified the allelic configuration for each CNV so that the modification was

applied to the proper side of the chromosome (e.g. for a copy number four duplication, if the allelic configuration was ABBB, we duplicated the sequence only from the B allele two more times; if its allelic configuration was AABB, we duplicated the sequences from both alleles once). After creating the hypothetical chromosome, we applied the sequencing simulator, wgsim, as implemented in SAMTools to generate 100bps paired-end short reads. We used the default wgsim values. In total, 50 millions read pairs were generated and yielded on average 40X coverage. We extracted allele specific reads using NA12891 heterozygous SNPs. Finally, we used BWA to align the reads back to unmodified reference to obtain TReC and ASReC.

3.2 WES simulation

We developed a pipeline to simulate the TReC and ASReC of WES data for 100 samples (see Figure S18). It consists of 11 steps summarized here. First, we selected chromosome 11 of one HapMap sample (HG00264) as template. From this template we obtained the exome-capturing target, its TReC, and the sum of the TReCs of all targets. We retained only targets in copy-number-two regions, which were extracted from 1000GP-released CNV analysis results. The next step was to simulate TReC. For each target in the template, we calculated the ratio between its TReC and the sum TReCs of all targets; this serves as the expected ratio of the target. We first simulated the sum of the TReCs of all the targets, to provide a sample that followed a normal distribution with the mean equal to the sum of the template TReCs. We then simulated the expected TReCs, target by target. To simulate the expected TReC for one target, we multiplied the template expected ratio by the sum TReCs of the sample. After simulating the expected TReCs for all targets, we use the garsim function to simulate the overdispersed TReCs. We passed the covariates matrix (using the expected TReCs at each target as the mean value) and coefficient vectors (all 1 for targets) into the garsim function, setting the distribution family as negative-binomial distribution, and the overdispersion parameter as 0.05. The result gave TReCs at each target for one sample. We repeated this procedure 100 times to obtain the TReCs for 100 samples. The next step was to simulate CNV with different population frequencies. To simulate a CNV, we needed to choose the targets and the samples affected by this CNV. For each CNV, we randomly chose 1Ð3 consecutive targets and reduced the expected TReC (e.g., multiplying by 0.5 for state 1) or amplified the expected TReC (e.g., multiplying by two for state four) at these targets. To simulate samples with altered copy number in a population, we defined a variable called CNV frequency that identified the proportion of samples (among the 100 simulated samples) with copy number alterations. The CNV frequency ranges used in this study were 1%, 5%, 10% and 20%, to simulate both rare CNV and common CNV. For one CNV, only the TReCs of the affected individuals were altered. We simulated different numbers of CNVs for different CNV frequencies, because when the CNV frequency is low (1% or 5%), a simulated CNV would affect only a small number of samples, which would cause the average number of CNVs in each sample to be extremely low. This in turn made it difficult to give a reasonably large denominator in the calculations of sensitivity and FDR. Therefore, we simulated 1000 deletions/1000 duplications with CNV frequencies of 1% and 5% and 200 deletions/200 duplications with frequencies of 10% and 20%. The last step was to simulate ASReC. We first simulated ASReC,following a binomial distribution with the mean value being 5% of the RD in the corresponding target. We then simulated the allelic configuration according to the copy number of the target (e.g., AAB or ABB for copy number 3) and obtained the proportion p of allele A (e.g., 0.33 for ABB). Finally we simulated the ASReC of allele A, which follows a beta-binomial distribution with the mean value of p multiplying the corresponding ASReCs and

the overdispersion being 0.1.

4 Reference

References

[1] J A Bilmes. A gentle tutorial of the EM algorithm and its application to parameter estimation for Gaussian mixture and hidden Markov models, 1998.

[2] C B Dean. Testing for Overdispersion in Poisson and Binomial Regression Models. *Journal of the American Statistical Association*, 87(418):451–457, 1992.

[3] Mark A DePristo, Eric Banks, Ryan Poplin, Kiran V Garimella, Jared R Maguire, Christopher Hartl, Anthony A Philippakis, Guillermo del Angel, Manuel A Rivas, Matt Hanna, Aaron McKenna, Tim J Fennell, Andrew M Kernytsky, Andrey Y Sivachenko, Kristian Cibulskis, Stacey B Gabriel, David Altshuler, and Mark J Daly. A framework for variation discovery and genotyping using next-generation DNA sequencing data. *Nature genetics*, 43(5):491–498, 2011.

[4] Menachem Fromer, Jennifer L Moran, Kimberly Chambert, Eric Banks, Sarah E Bergen, Douglas M Ruderfer, Robert E Handsaker, Steven a McCarroll, Michael C O'Donovan, Michael J Owen, George Kirov, Patrick F Sullivan, Christina M Hultman, Pamela Sklar, and Shaun M Purcell. Discovery and statistical genotyping of copy-number variation from whole-exome sequencing depth. *American journal of human genetics*, 91(4):597–607, October 2012.

[5] Biing-Hwang Juang and Lawrence R. Rabiner. Mixture autoregressive hidden Markov models for speech signals. *IEEE Transactions on Acoustics, Speech, and Signal Processing*, 33(6):1404–1413, 1985.

[6] Heng Li and Richard Durbin. Fast and accurate short read alignment with Burrows-Wheeler transform. *Bioinformatics (Oxford, England)*, 25(14):1754–60, July 2009.

[7] Heng Li, Bob Handsaker, Alec Wysoker, Tim Fennell, Jue Ruan, Nils Homer, Gabor Marth, Goncalo Abecasis, and Richard Durbin. The Sequence Alignment/Map format and SAMtools. *Bioinformatics (Oxford, England)*, 25(16):2078–9, August 2009.

[8] Eric Yi Liu, Mingyao Li, Wei Wang, and Yun Li. MaCH-admix: genotype imputation for admixed populations. *Genetic epidemiology*, 37(1):25–37, 2013.

[9] Aaron McKenna, Matthew Hanna, Eric Banks, Andrey Sivachenko, Kristian Cibulskis, Andrew Kernytsky, Kiran Garimella, David Altshuler, Stacey Gabriel, Mark Daly, and Mark A. DePristo. The genome analysis toolkit: A MapReduce framework for analyzing next-generation DNA sequencing data. *Genome Research*, 20(9):1297–1303, 2010.

[10] Jin P Szatkiewicz, WeiBo Wang, Patrick F Sullivan, Wei Wang, and Wei Sun. Improving detection of copy-number variation by simultaneous bias correction and read-depth segmentation. *Nucleic acids research*, 41(3):1519–32, 2013.

[11] Geraldine A. Van der Auwera, Mauricio O. Carneiro, Christopher Hartl, Ryan Poplin, Guillermo del Angel, Ami Levy-Moonshine, Tadeusz Jordan, Khalid Shakir, David Roazen, Joel Thibault, Eric Banks, Kiran V. Garimella, David Altshuler, Stacey Gabriel, and Mark A. DePristo. *Current Protocols in Bioinformatics.* Number SUPL.43. John Wiley & Sons, Inc., Hoboken, NJ, USA, August 2002.

[12] Norman Weiss, George Soules, Leonard E. Baum, and Ted Petrie. A Maximization Technique Occurring in the Statistical Analysis of Probabilistic Functions of Markov Chains, 1970.

Figure S1: Example of ASCNV in WGS simulation data

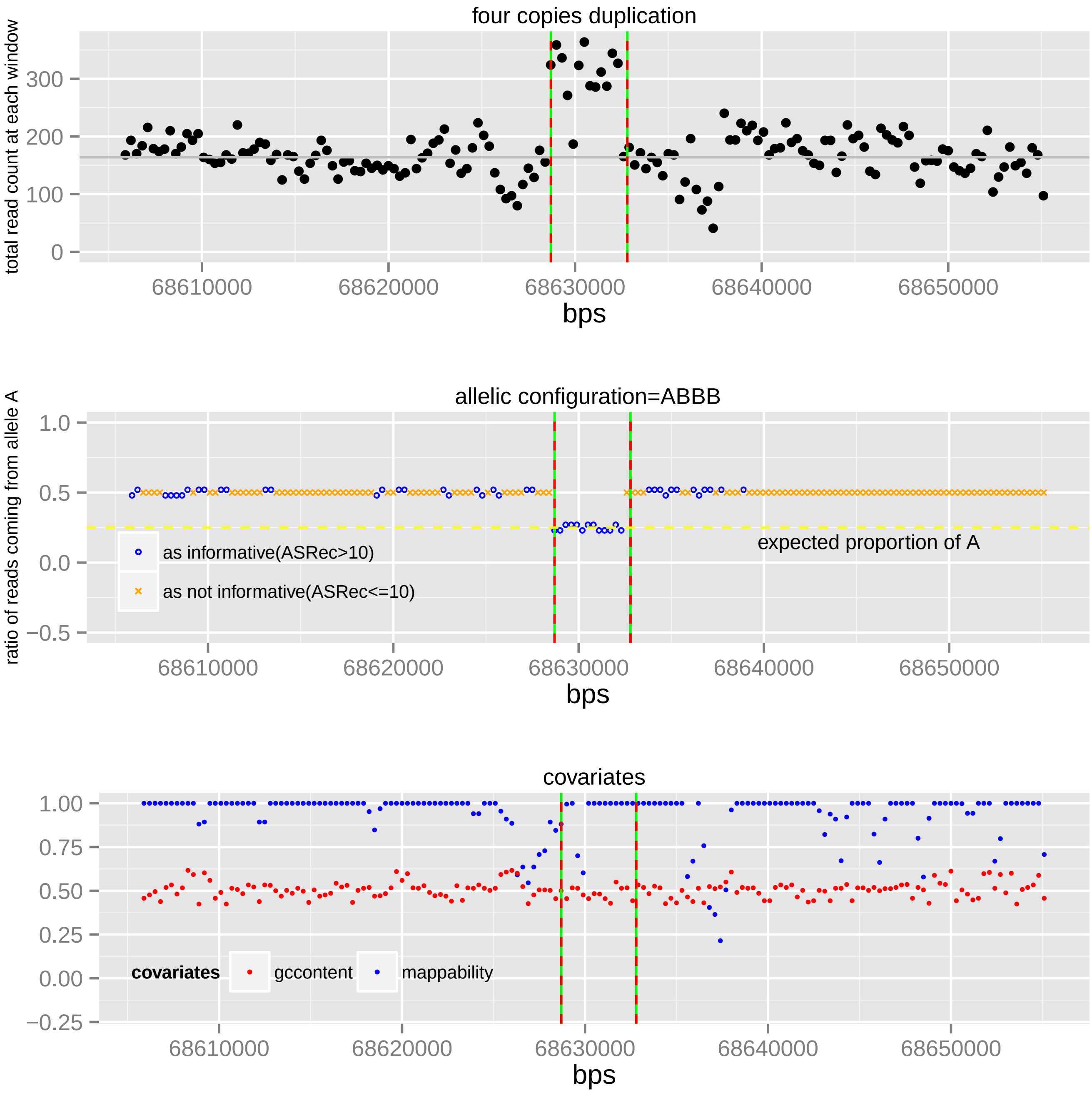

Figure S2: Example of ASCNV in WGS simulation data

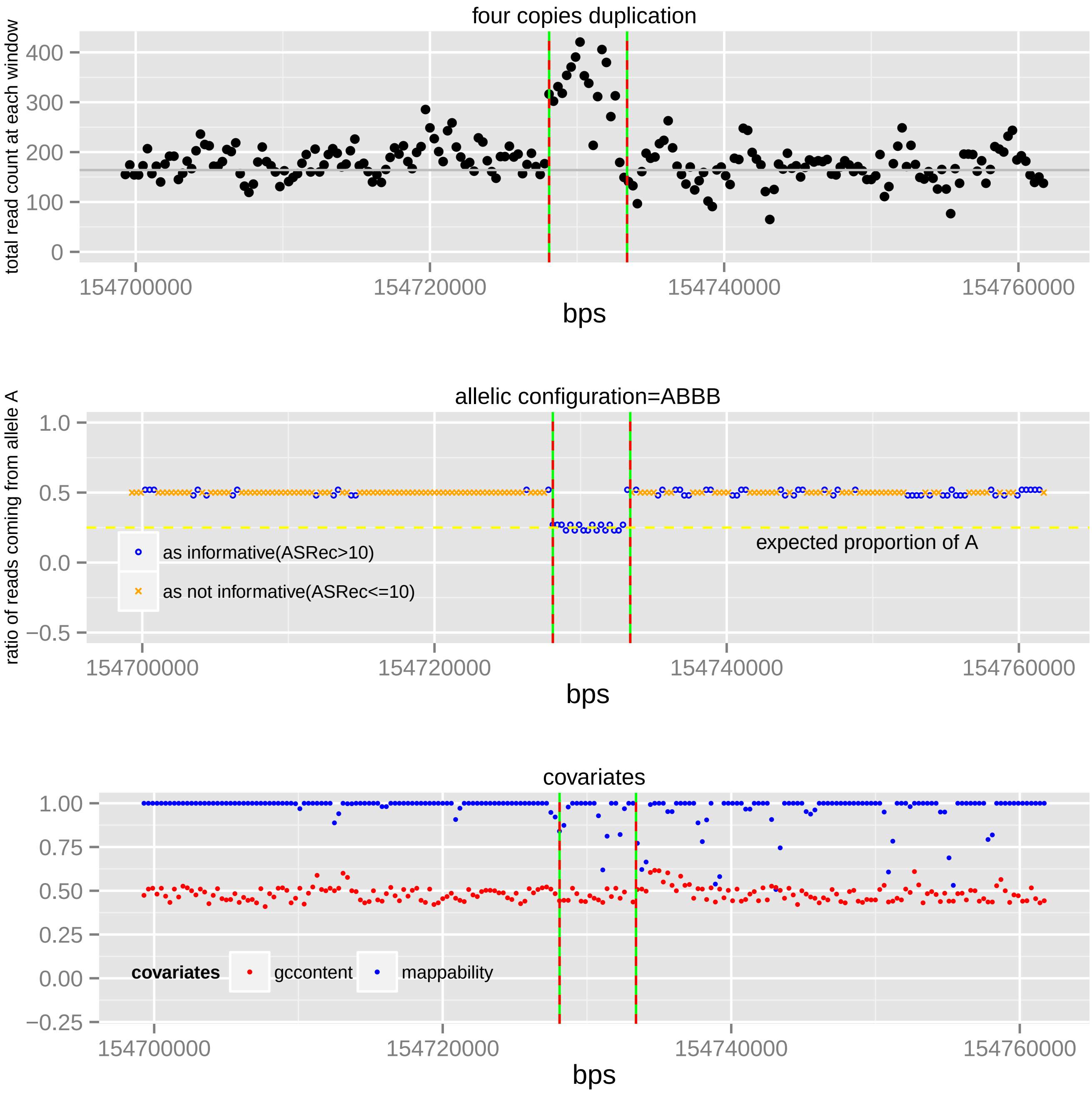

Figure S3: Detected high-confidence deletion after incorporating AS information in WGS data

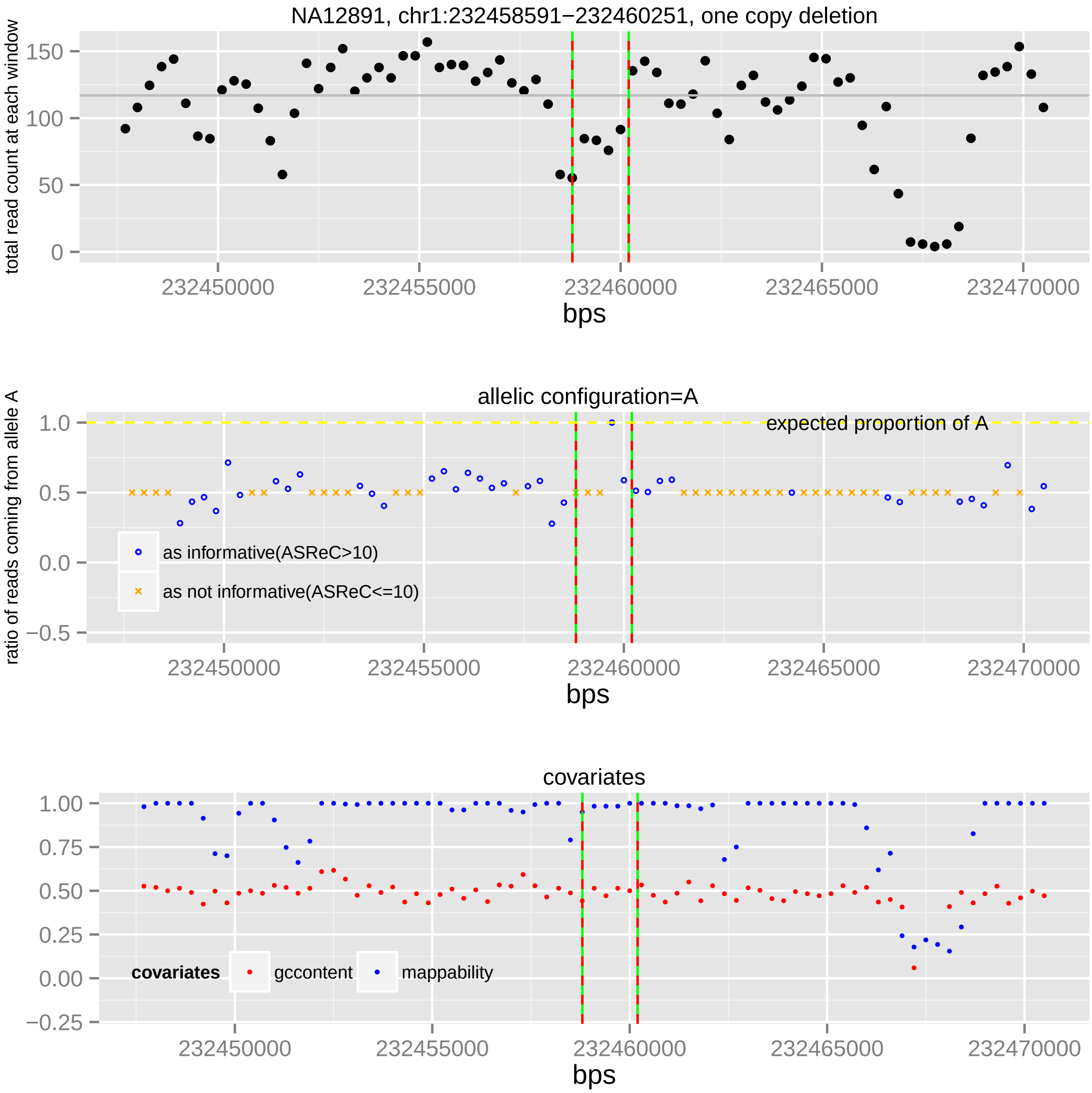

Figure S4: Detected high-confidence deletion after incorporating AS information in WGS data

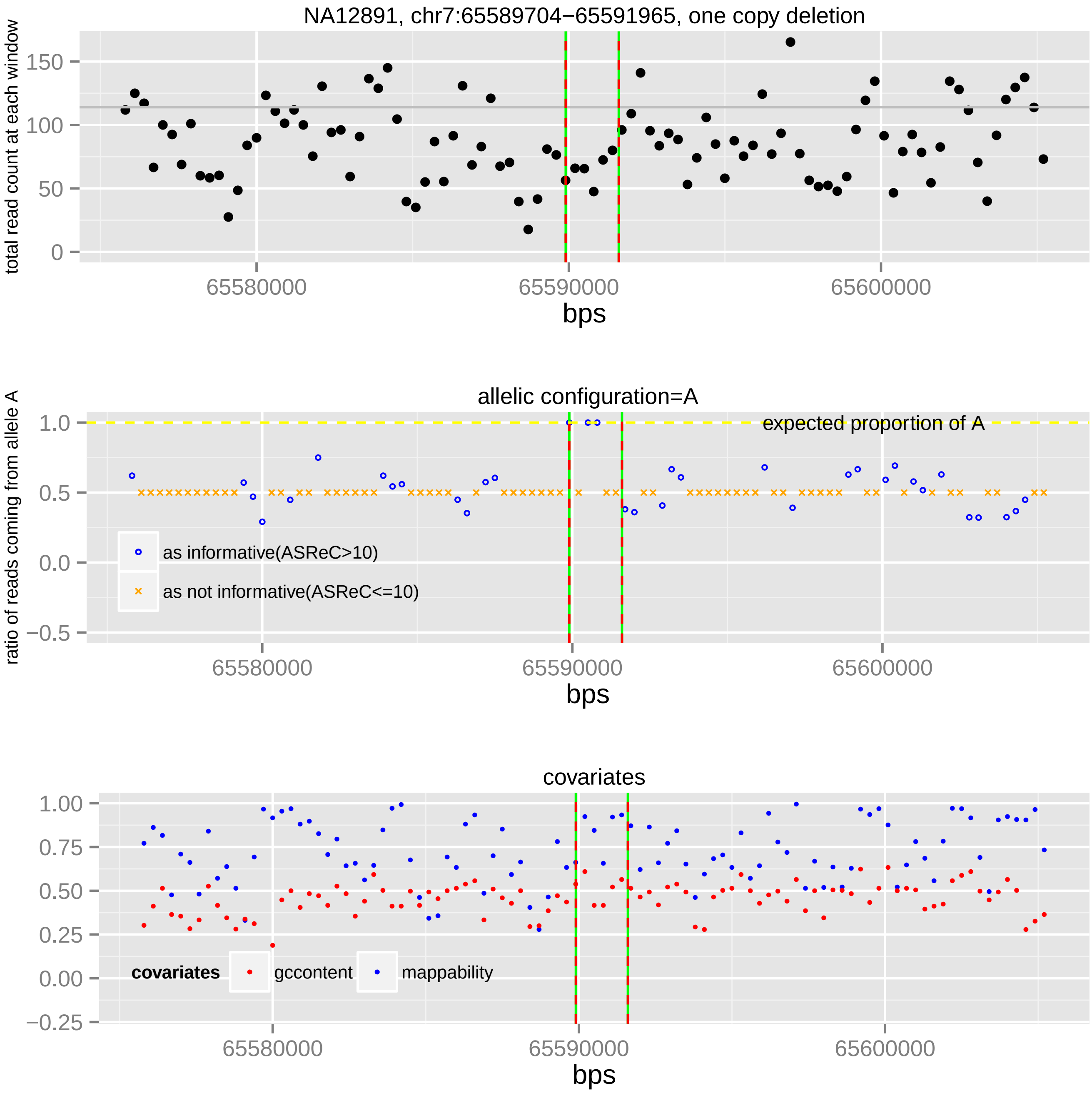

Figure S5: Detected high-confidence deletion after incorporating AS information in WGS data

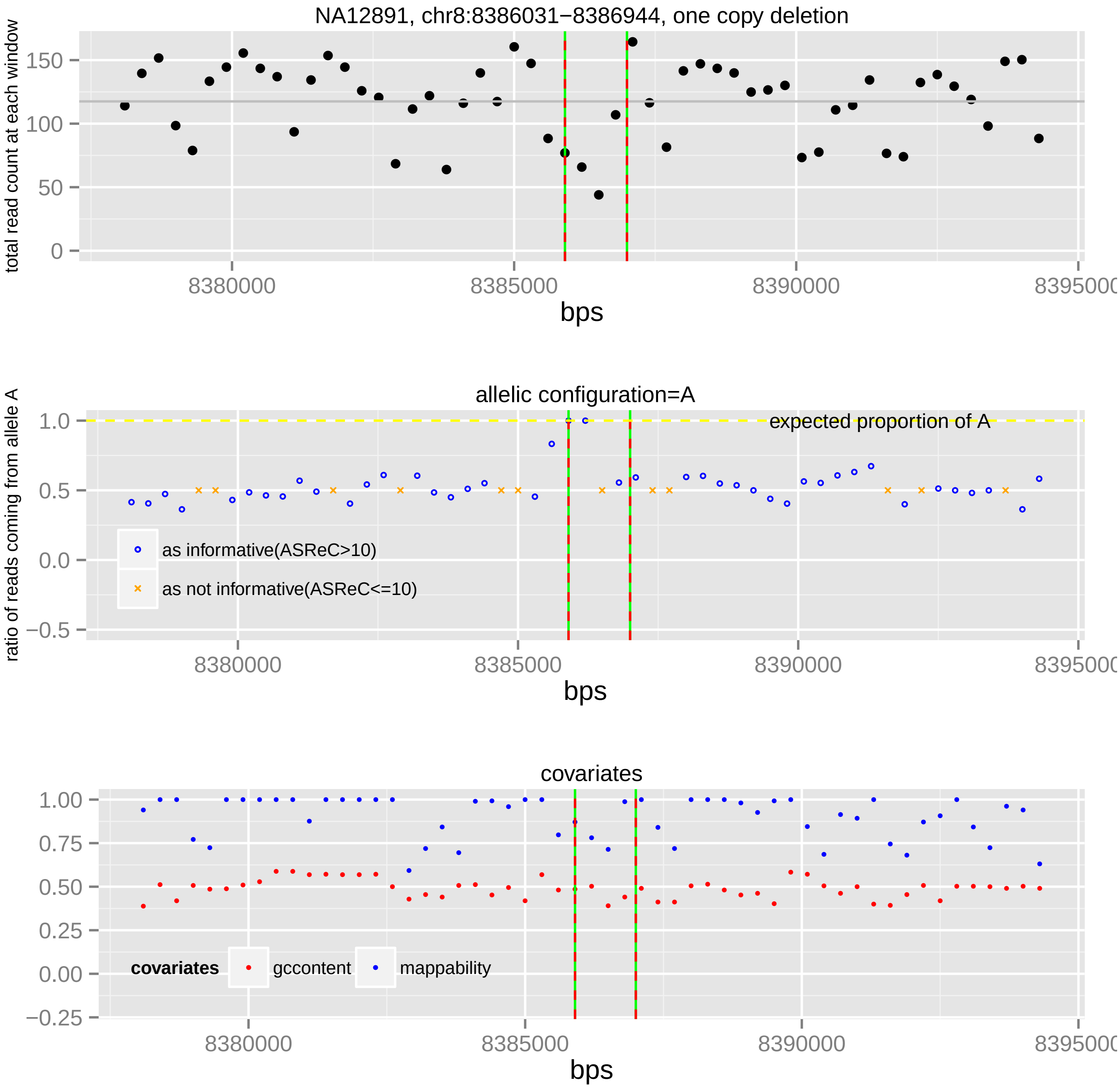

Figure S6: Detected high-confidence deletion after incorporating AS information in WGS data

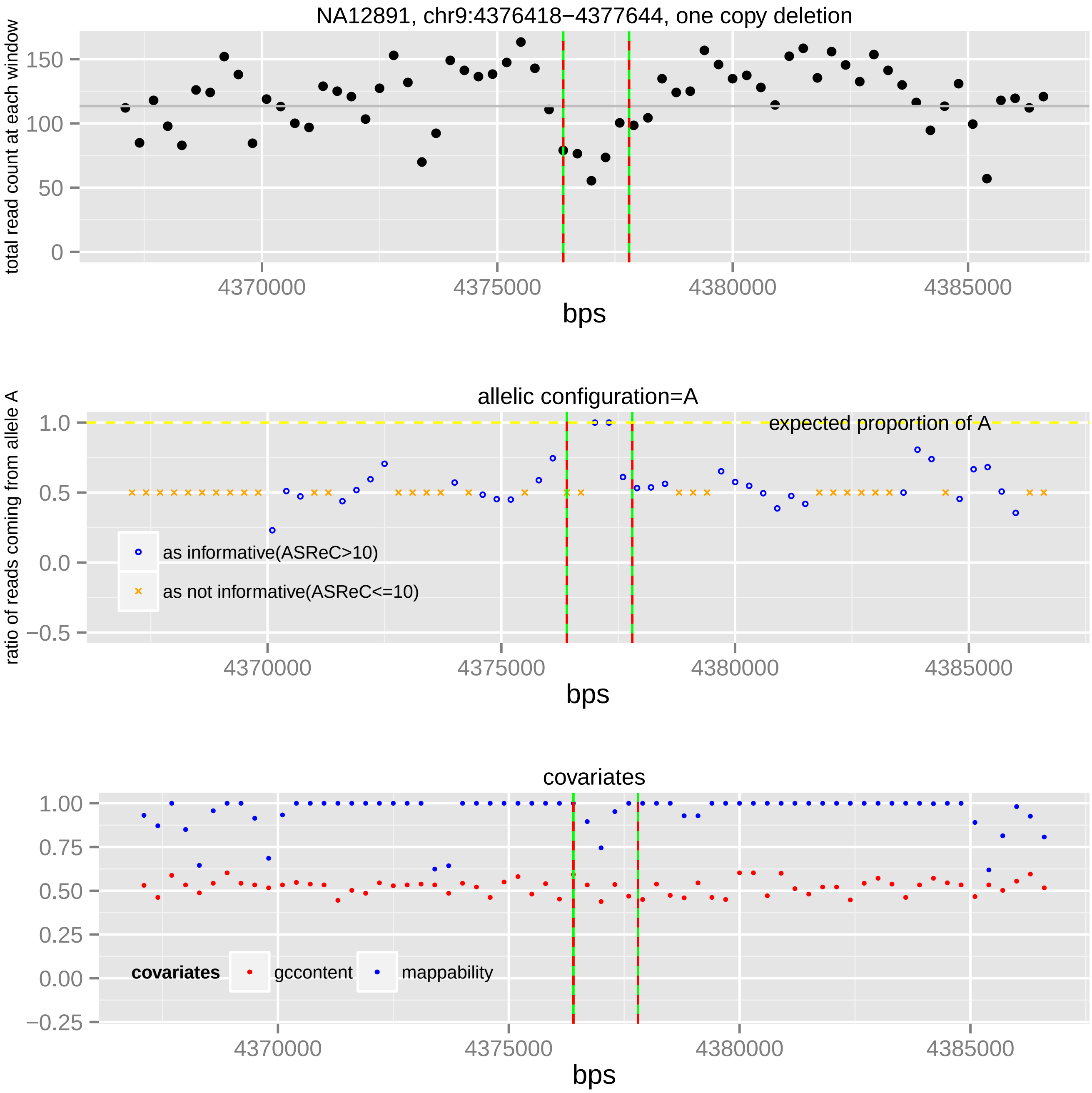

Figure S7: Detected high-confidence deletion after incorporating AS information in WGS data

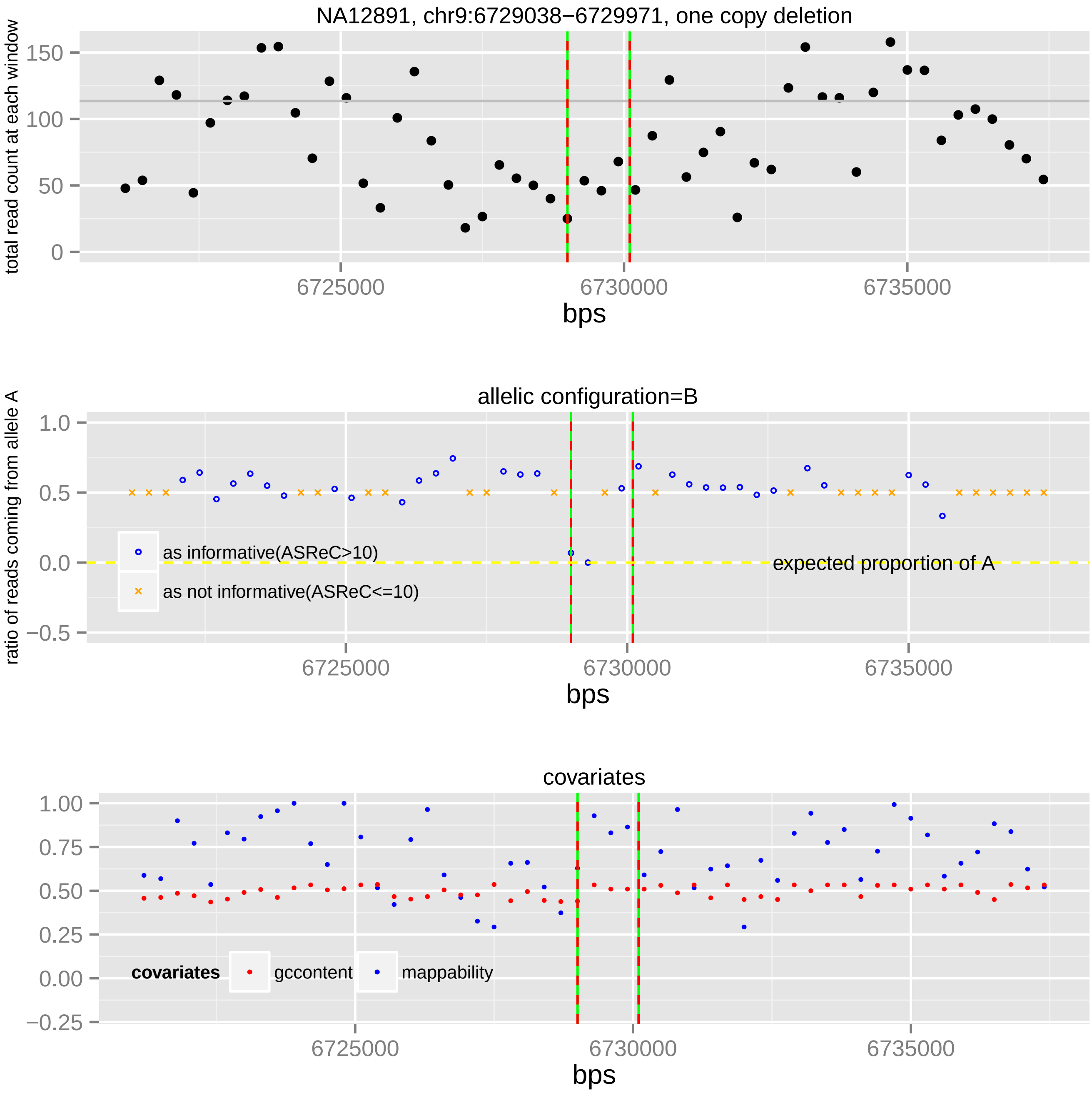

Figure S8: Detected high-confidence deletion after incorporating AS information in WGS data

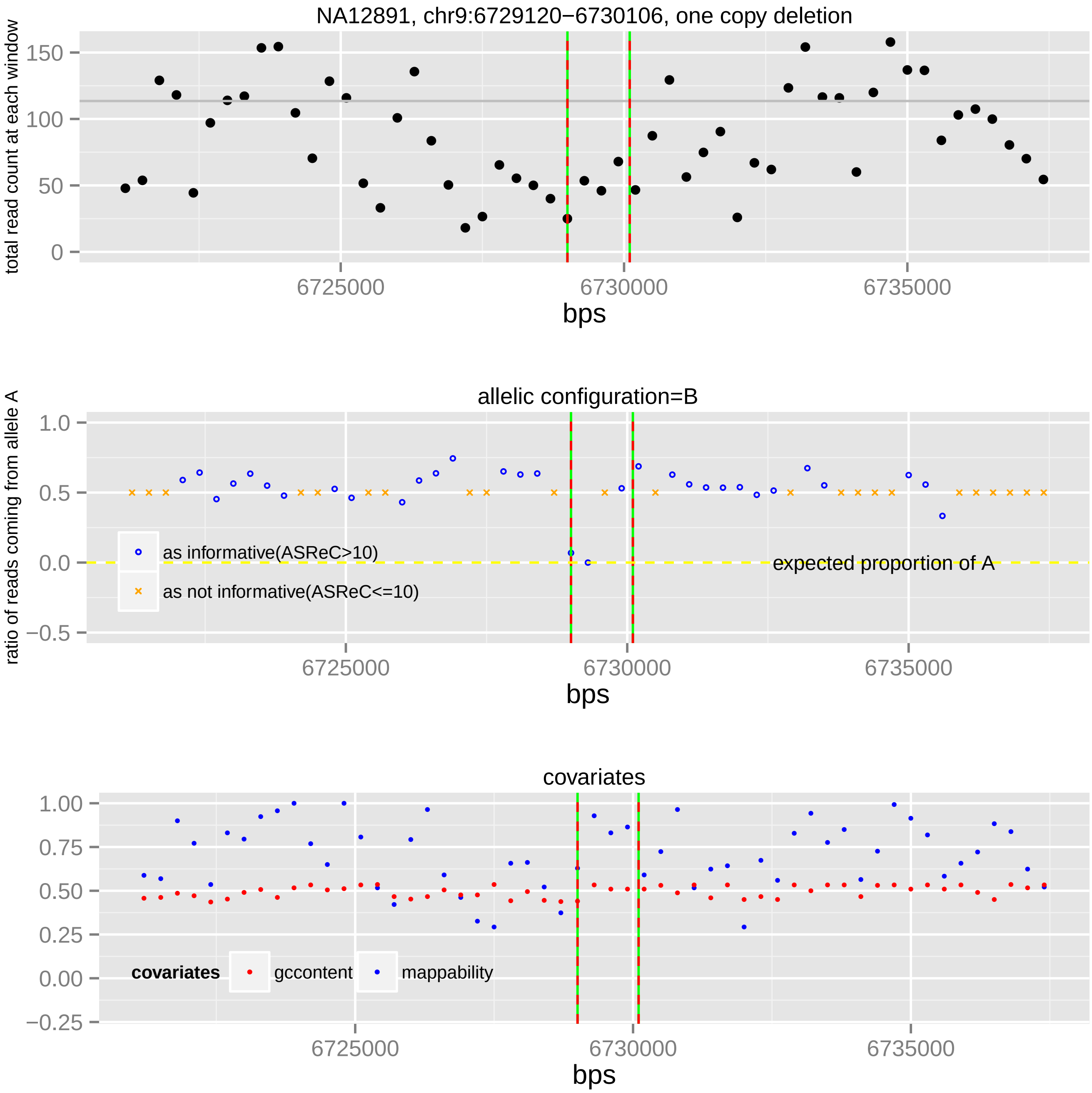

Figure S9: Detected high-confidence deletion after incorporating AS information in WGS data

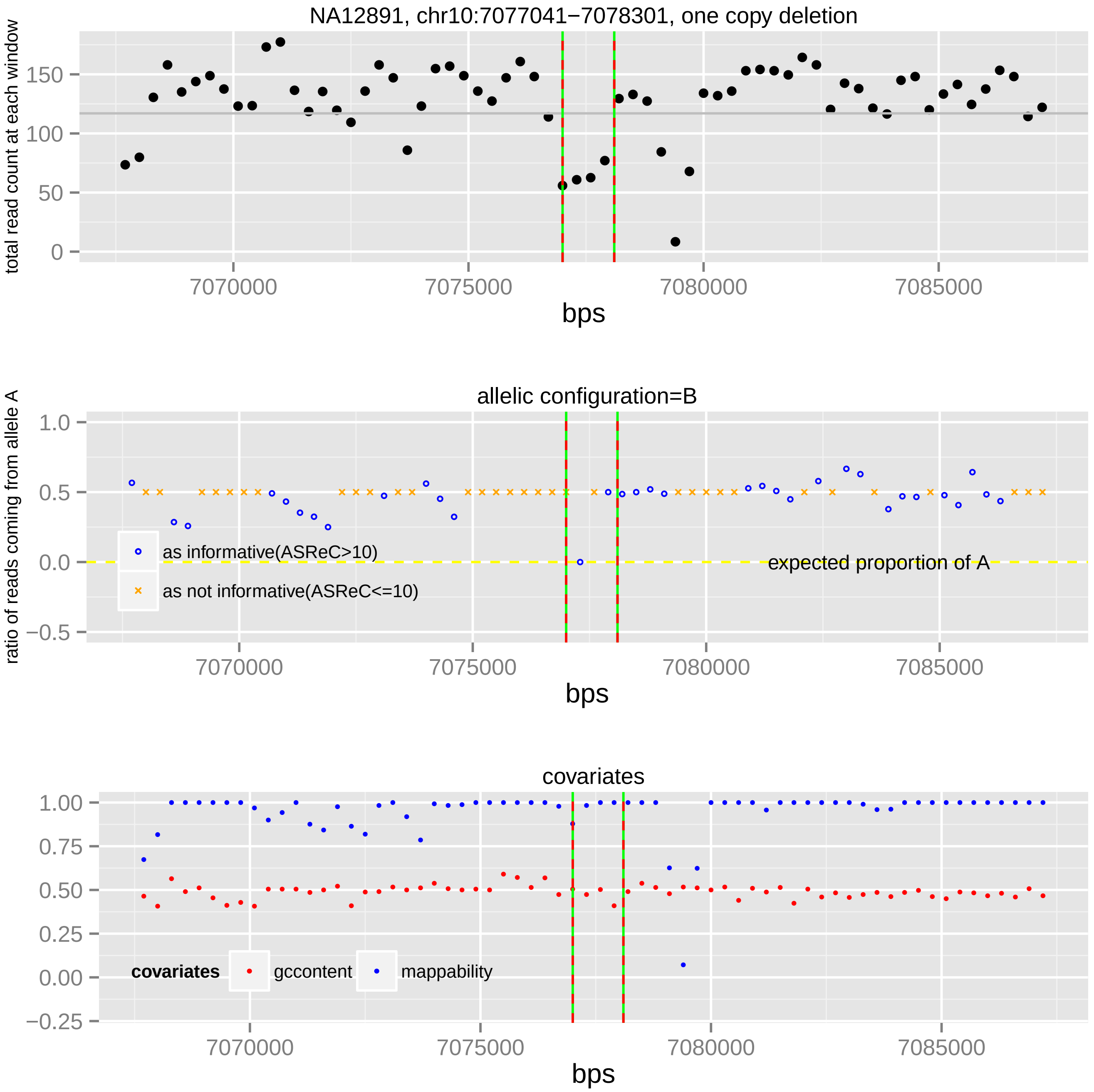

Figure S10: Detected high-confidence deletion after incorporating AS information in WGS data

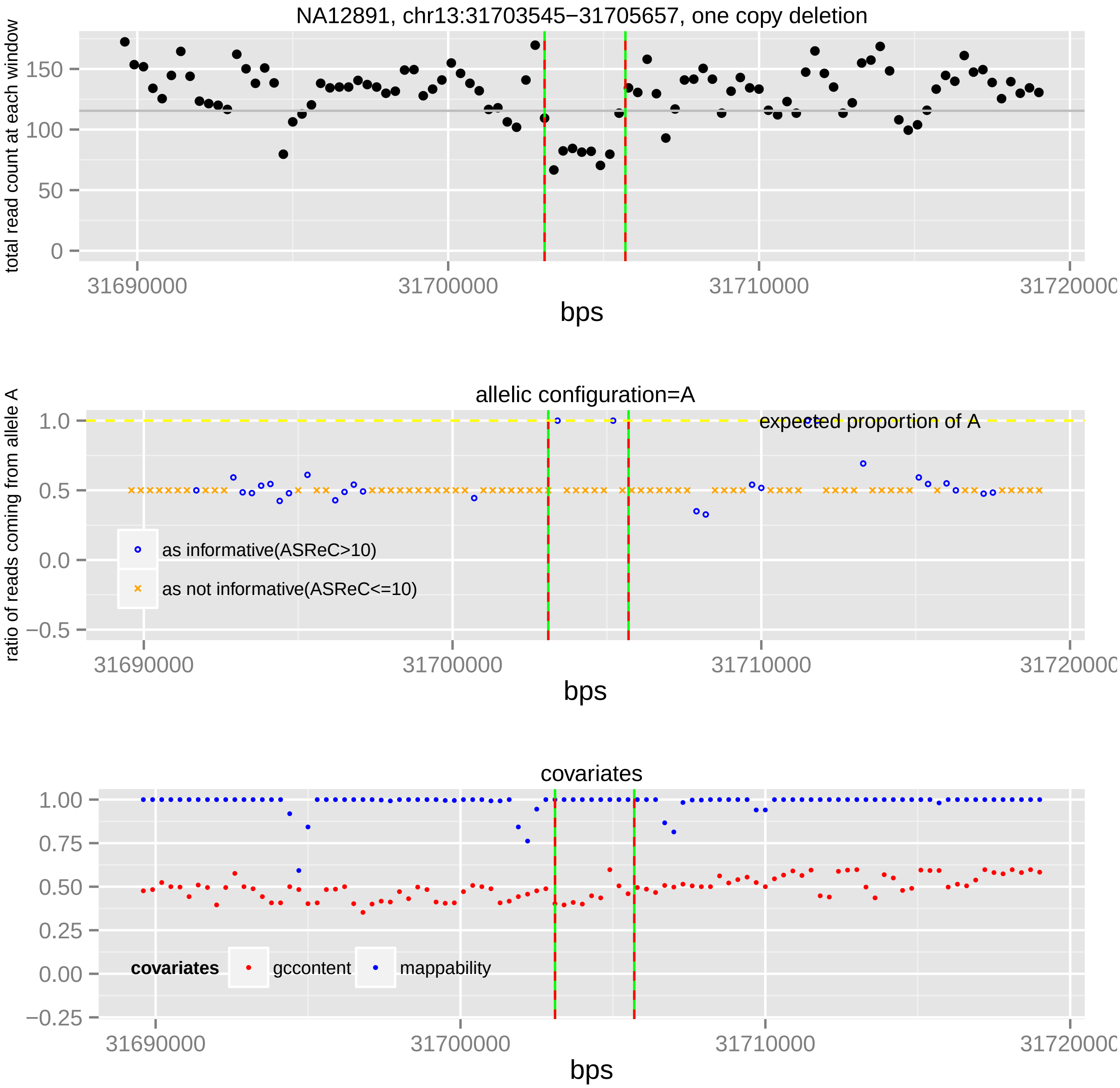

Figure S11: Detected high-confidence deletion after incorporating AS information in WGS data

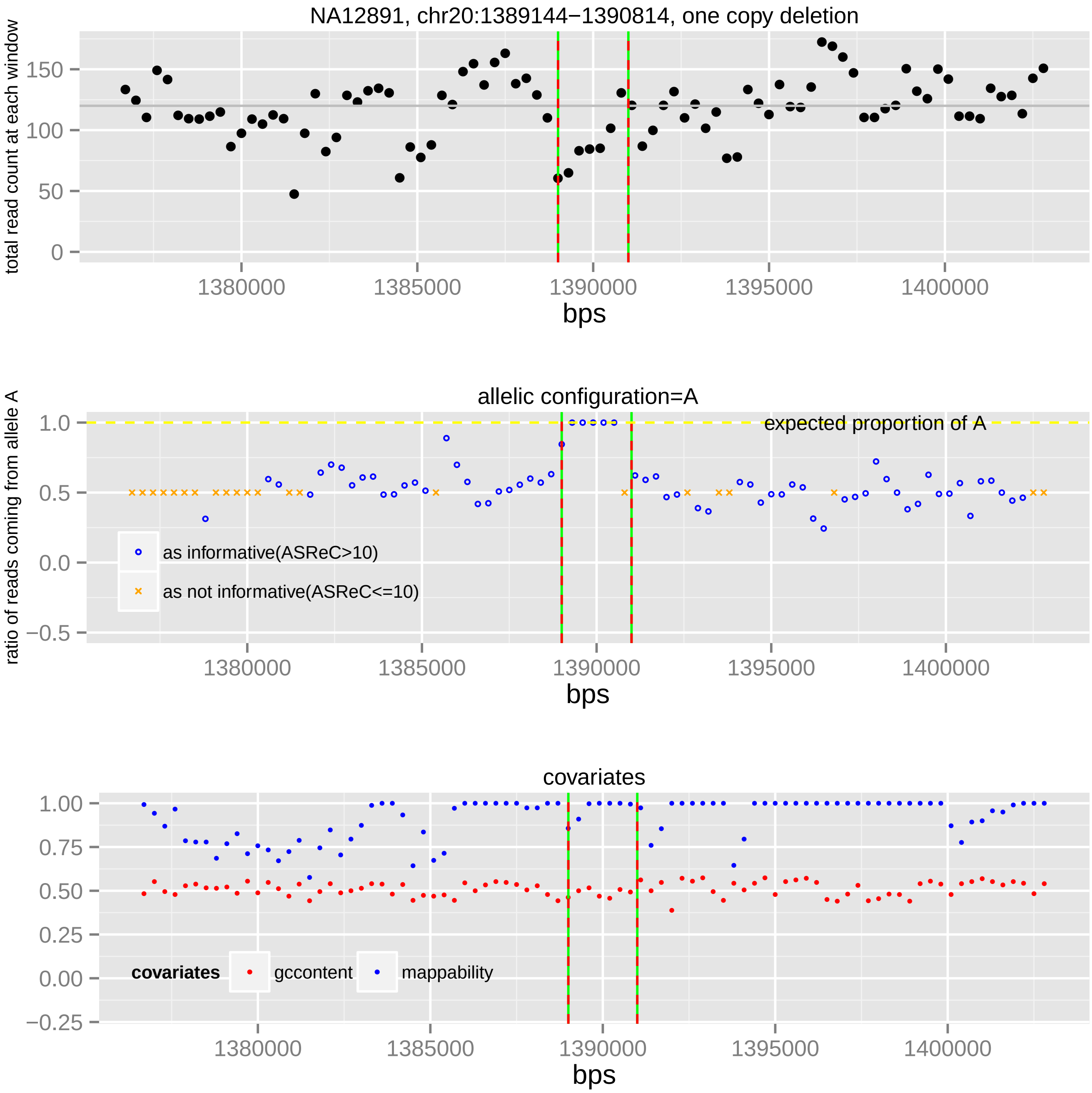

Figure S12: Detected high-confidence deletion after incorporating AS information in WGS data

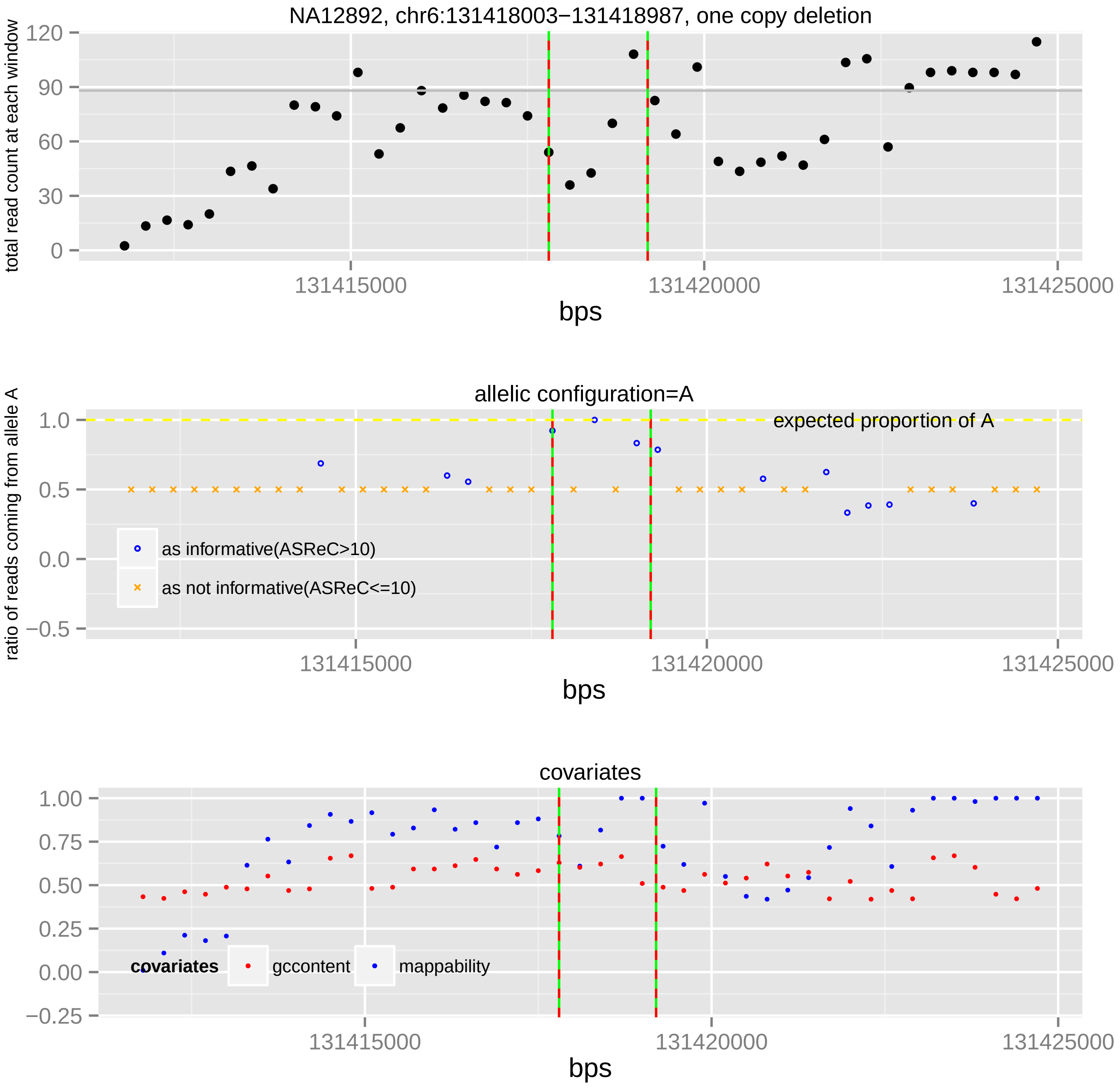

Figure S13: Detected high-confidence deletion after incorporating AS information in WGS data

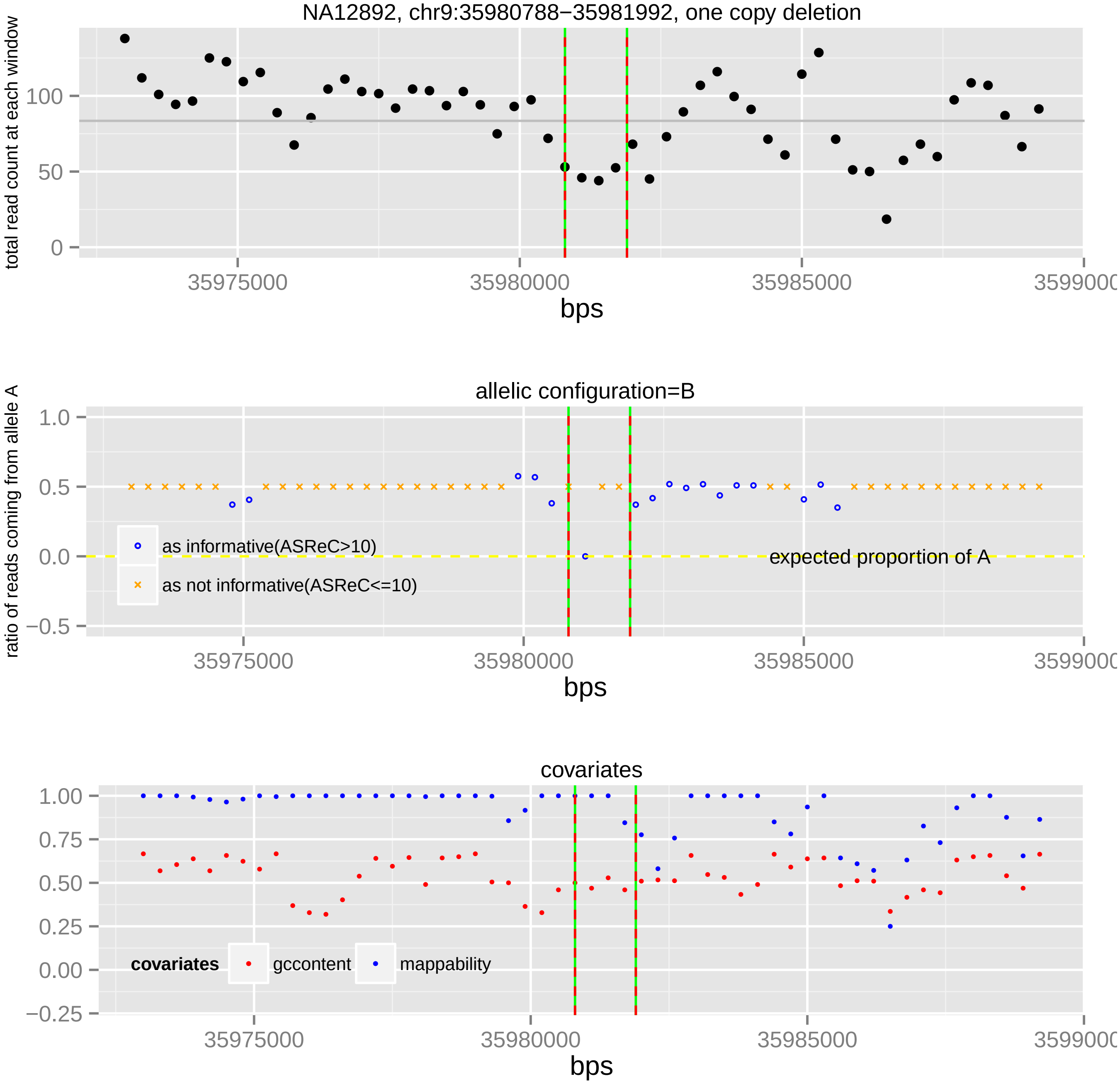

Figure S14: Detected high-confidence deletion after incorporating AS information in WGS data

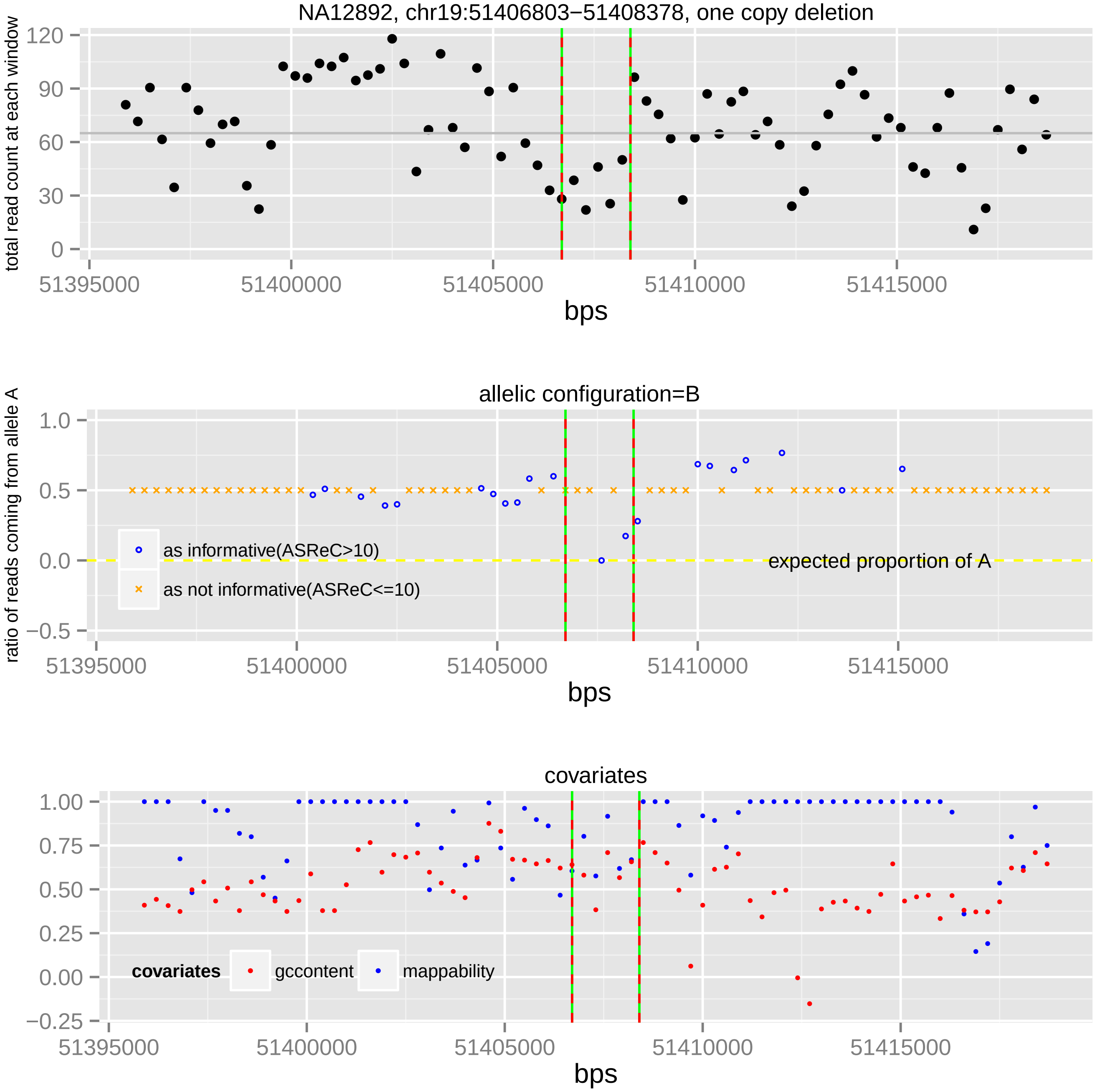

Figure S15: Detected high-confidence deletion after incorporating AS information in WGS data

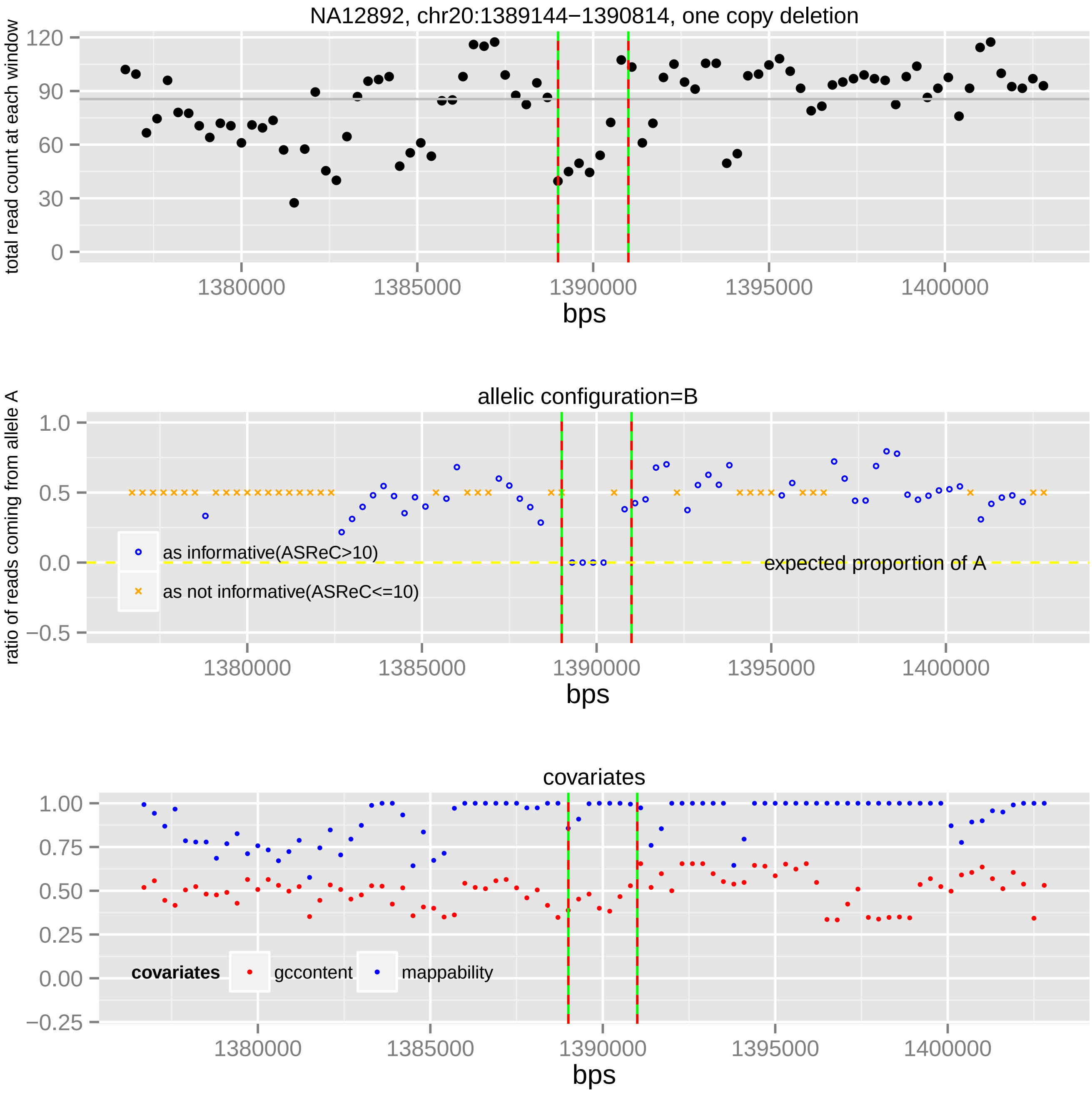

Figure S16: HMM flowchart to infer allele-specific CNV

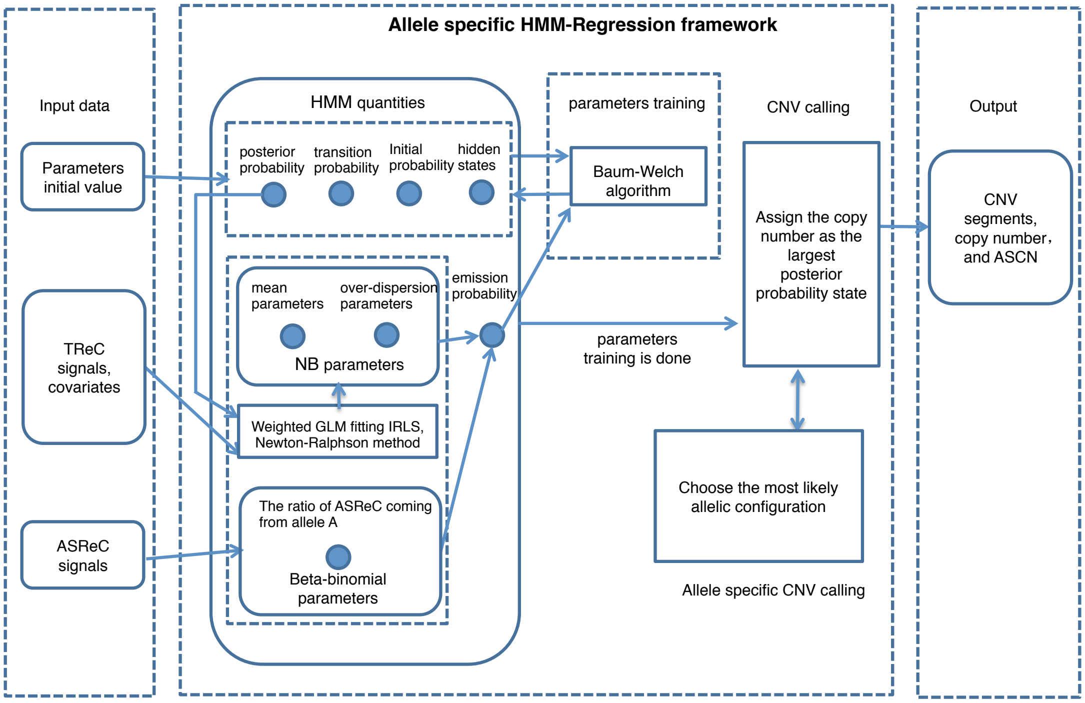

Figure S17: Illustration of sensitivity and FDR calculation

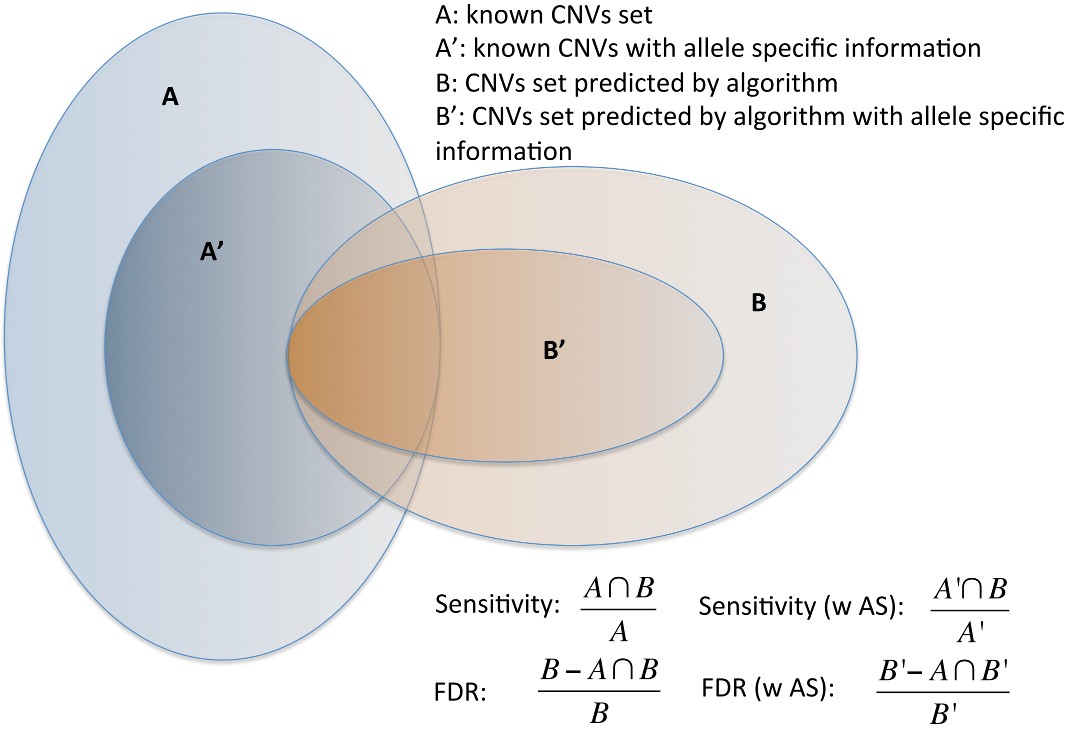

Figure S18: Whole-exome sequencing-simulation flowchart

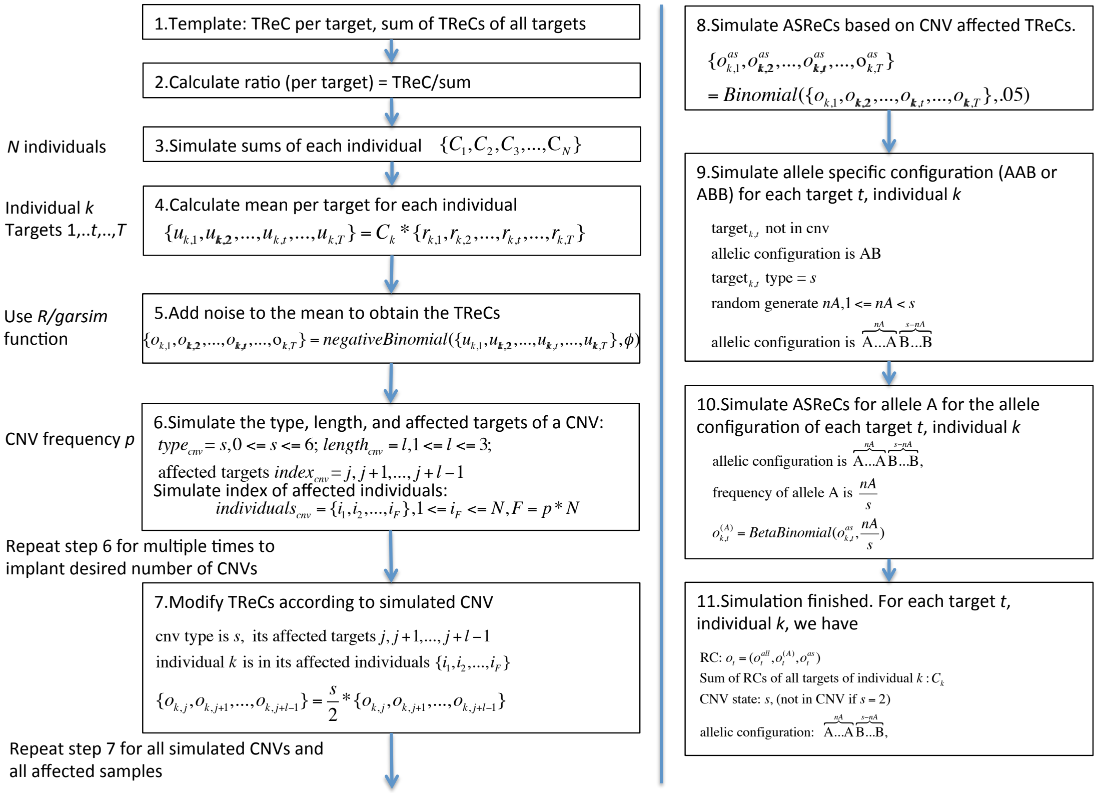

Figure S19: Better estimation of TReC for WES after incorporating AS information

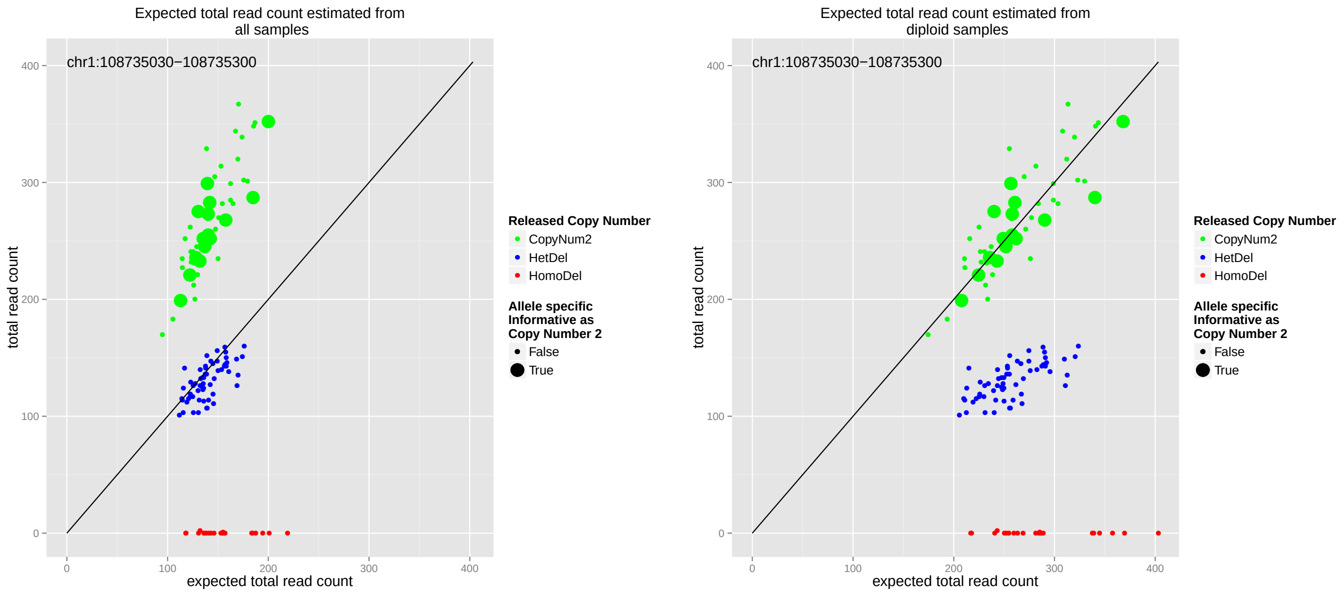

note

Figure S20: Better estimation of TReC for WES after incorporating AS information

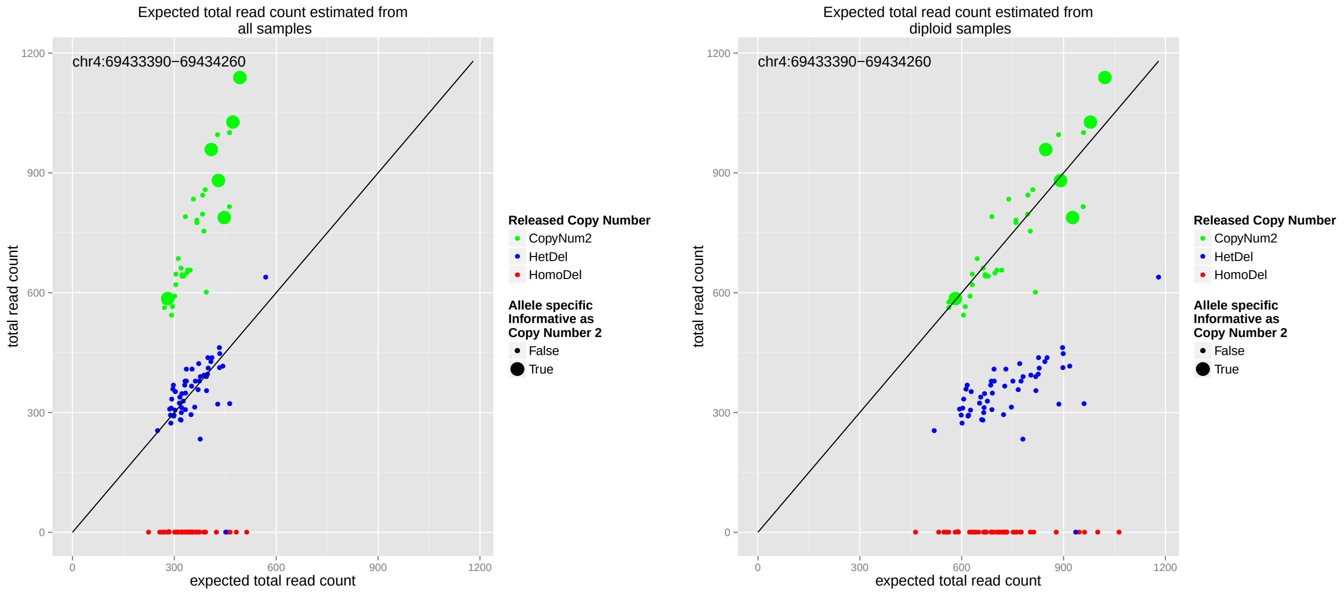

Figure S21: Better estimation of TReC for WES after incorporating AS information

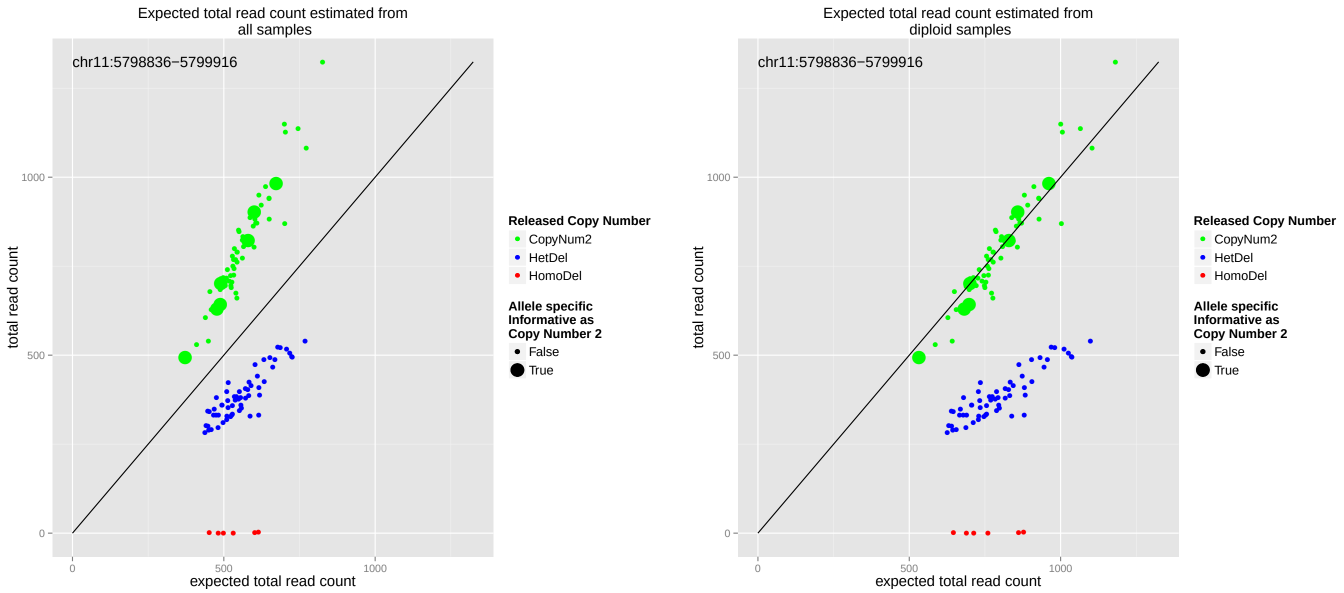

Figure S22: Better estimation of TReC for WES after incorporating AS information

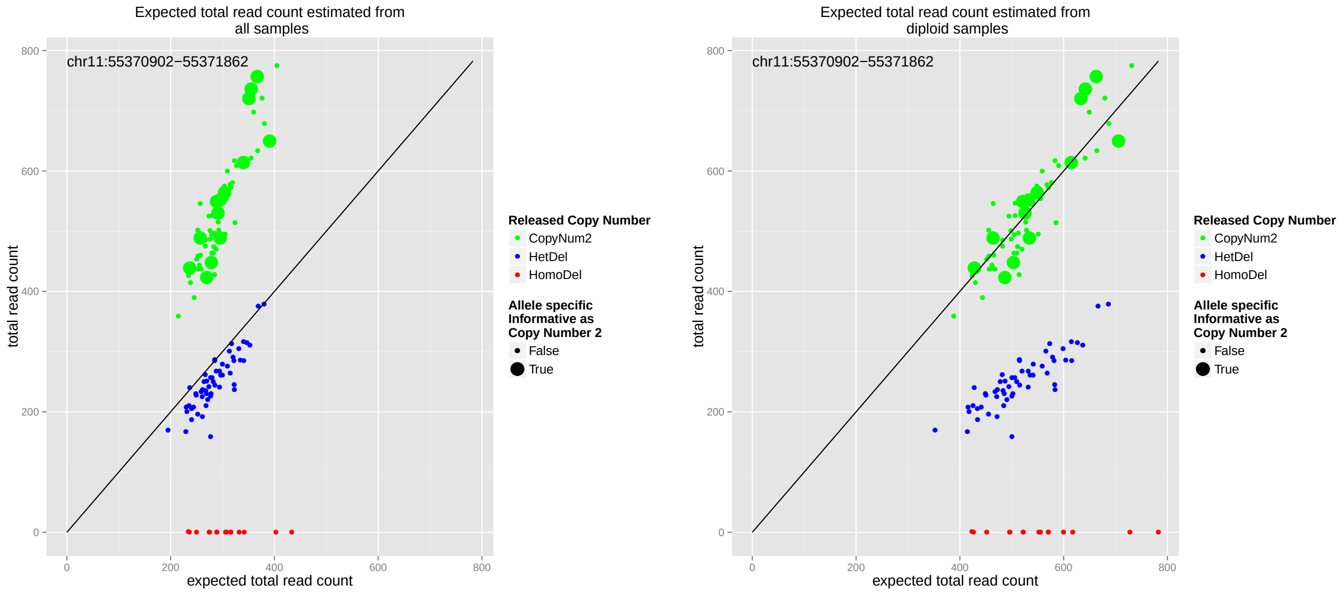

Figure S23: Better estimation of TReC for WES after incorporating AS information

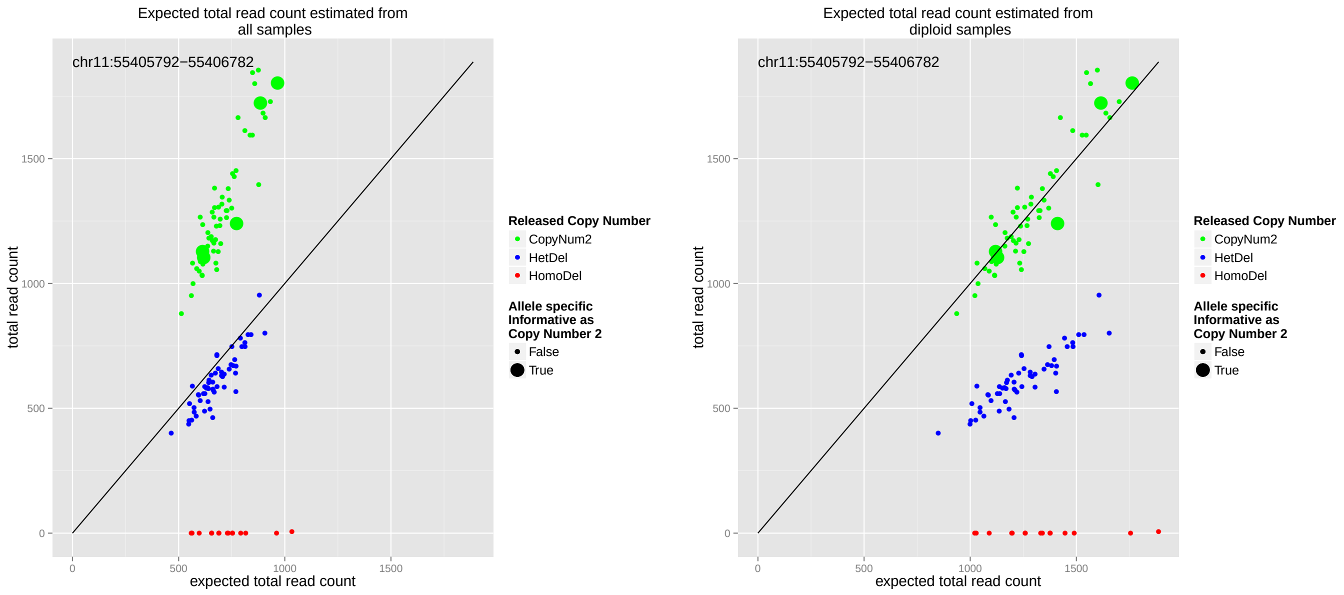

Figure S24: Better estimation of TReC for WES after incorporating AS information

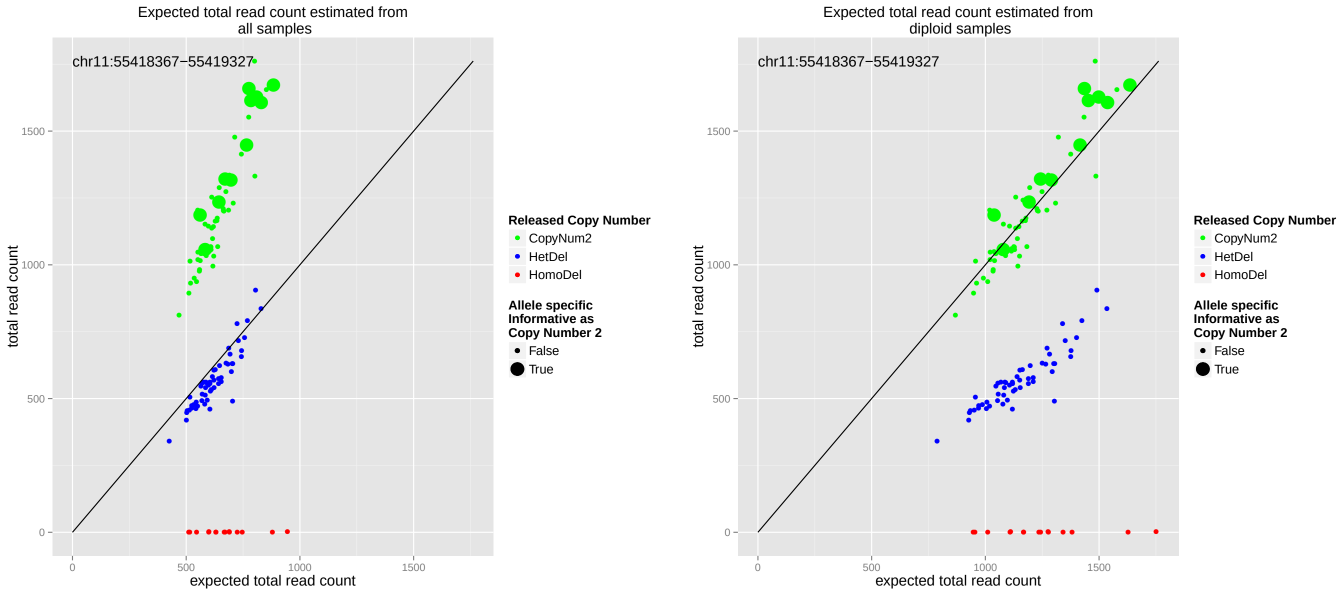

Figure S25: Better estimation of TReC for WES after incorporating AS information

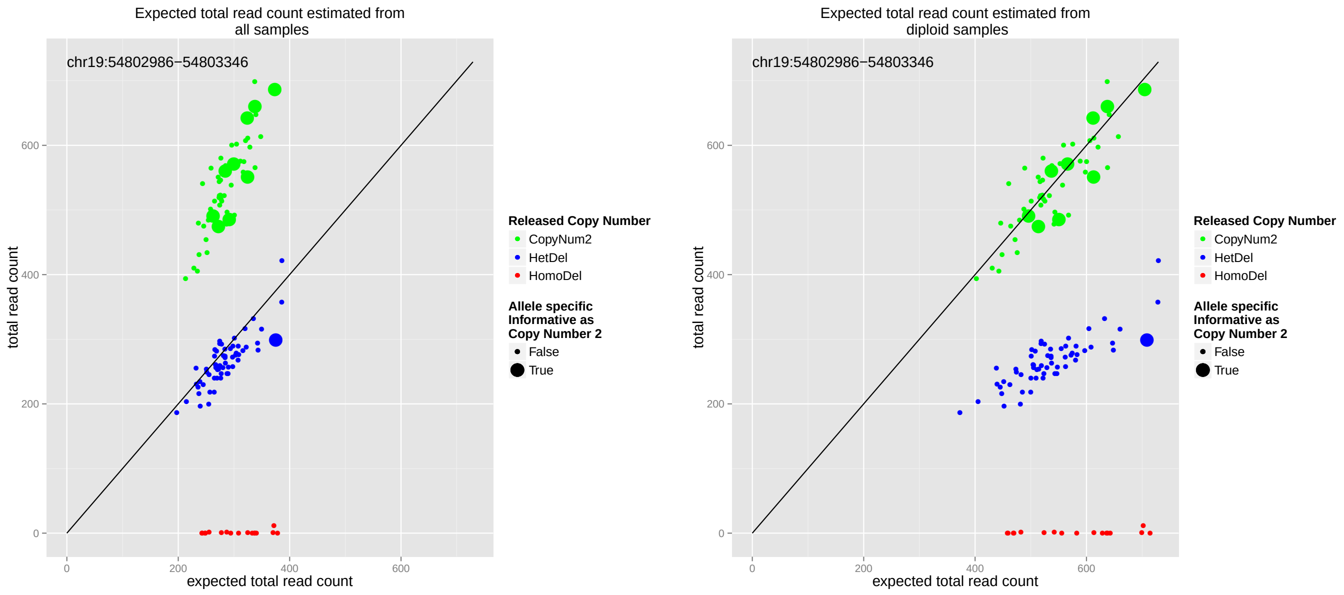

Figure S26: Better estimation of TReC for WES after incorporating AS information

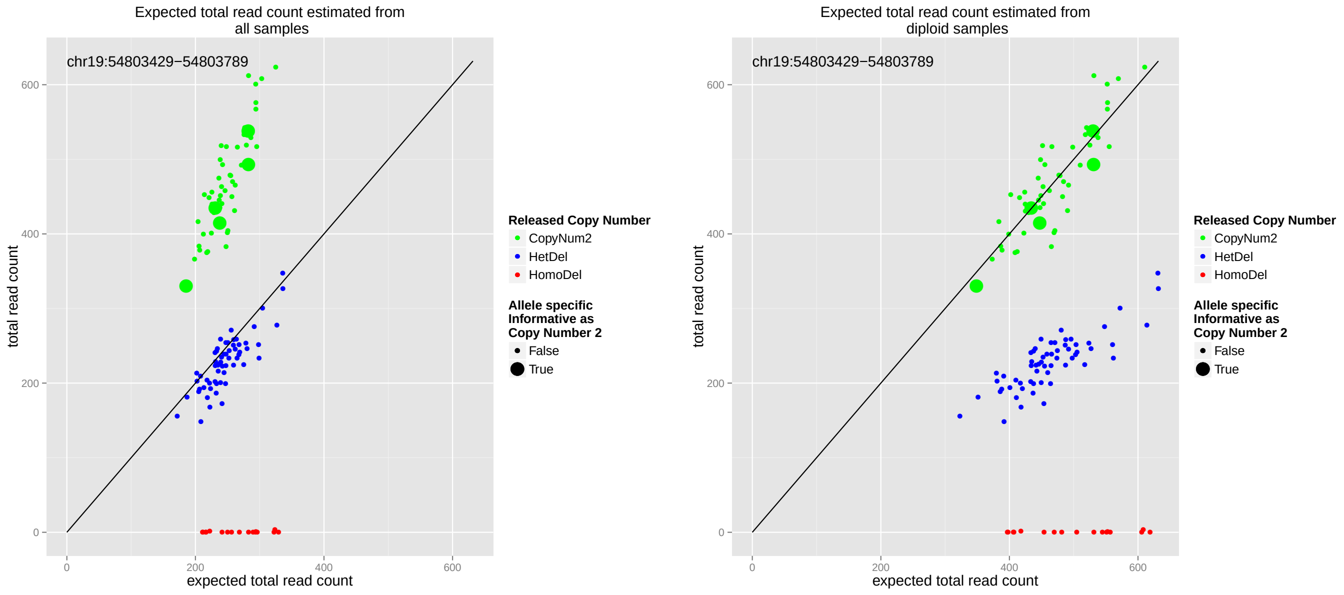

Figure S27: Better estimation of TReC for WES after incorporating AS information

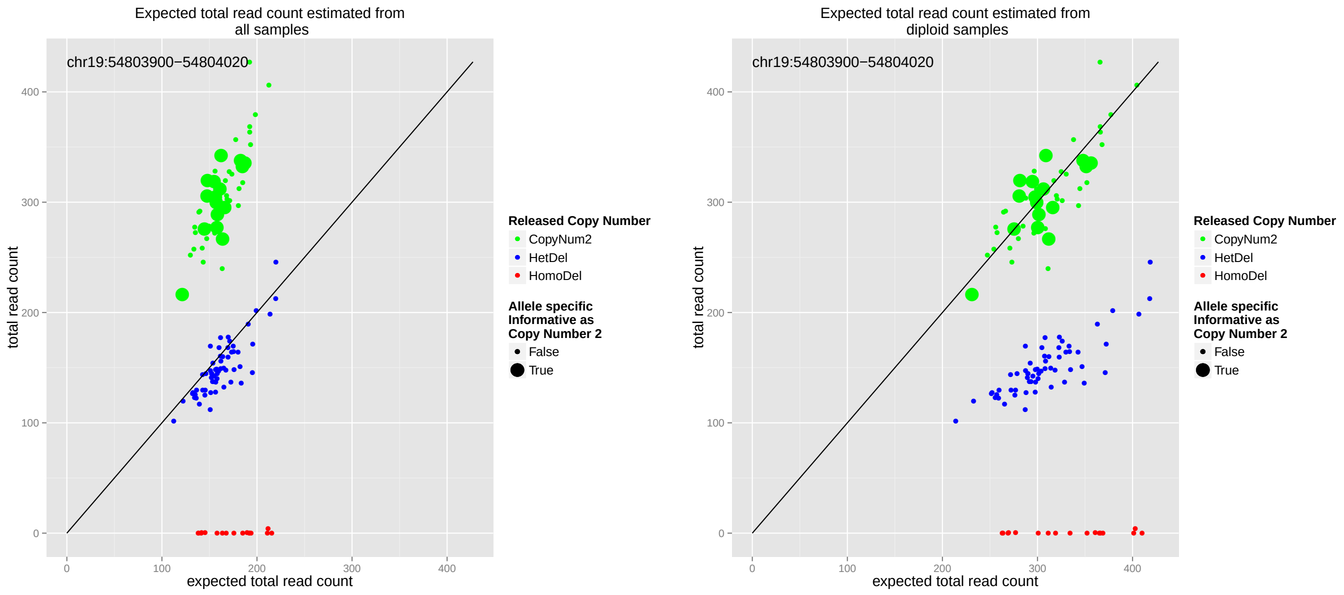

Figure S28: Better estimation of TReC for WES after incorporating AS information

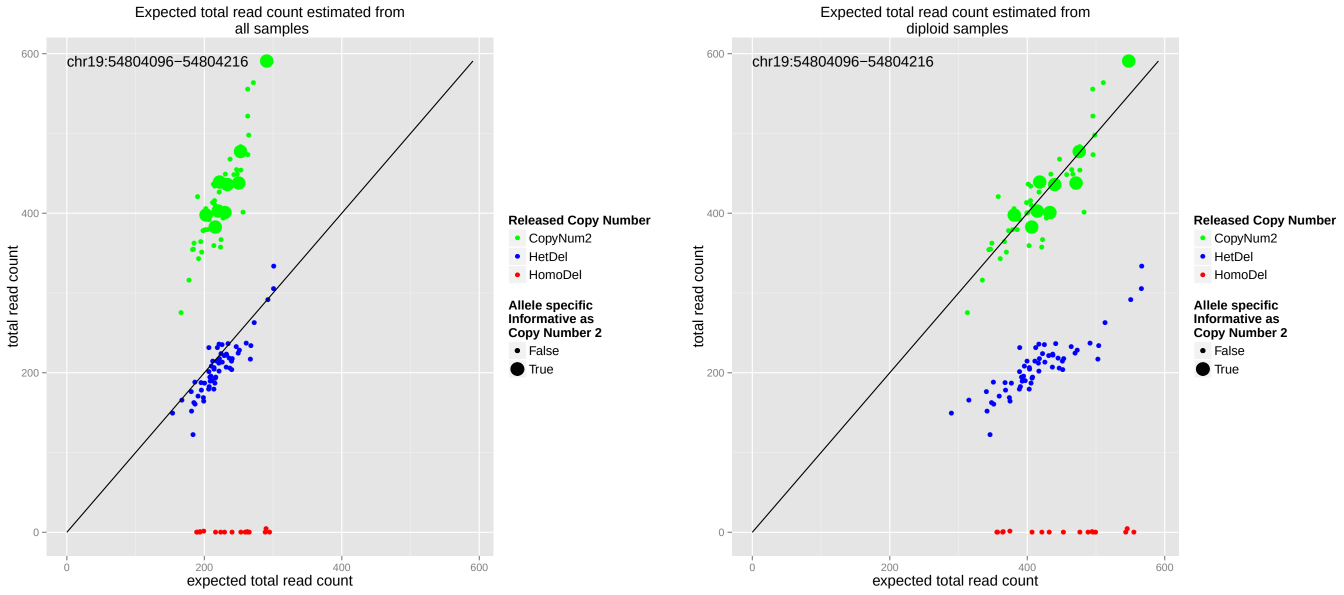

Figure S29: Better estimation of TReC for WES after incorporating AS information

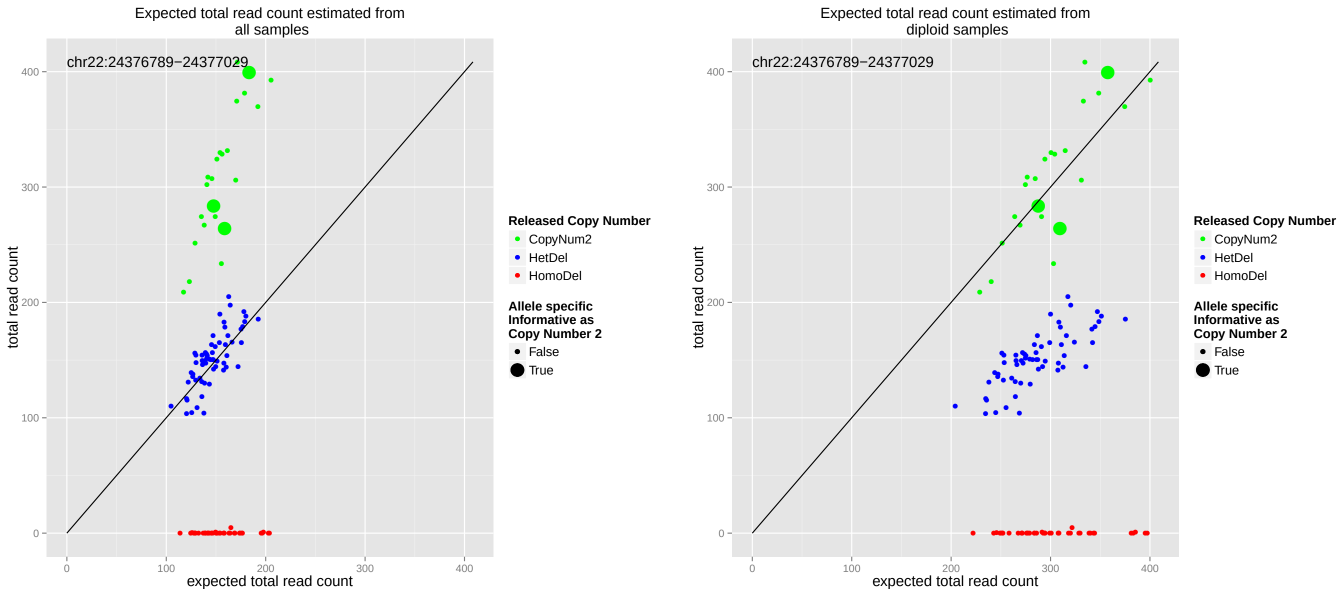

Table S1: Notations

Symbol	Explanation
settings for whole-genome sequencing data	
T	The total number of windows
A, B	Haplotypes A and B. The choice of A between two haplotypes is random.
$O = \{o_1, o_2, ..., o_T\}$	A vector of read count data. Element o_t contains the read count data of the tth window.
$o_t = (o_t^{all}, o_t^{(A)}, o_t^{as})$	read count data of tth window consists of three parts. o_t^{all} is generated from all reads aligned to the tth window, $o_t^{(A)}$ is generated from reads aligned to the tth window carrying heterozygous SNP(s) in haplotype A, and o_t^{as} is generated from reads aligned to the tth either carrying heterozygous SNP(s) in haplotype A or haplotype B.
$X = \{x_1, x_2, ..., x_T\}$	A vector of covariates data. Element x_t contains the covariates of the tth window.
$x_t = (g_t, l_t)$	Covariates of the tth window consists of two parts. g_t is the measurement of GC content of the tth window, and l_t is the measurement of mappability of the tth window.
$\Lambda = \{CN, \Pi, A, E\}$	Parameters of Hidden Markov Model
$CN = 0, 1, ...j, ..., N-1$	Copy number, which is the hidden state of the HMM model. There are N states in total.
$\Pi = (\pi_0, \pi_1, ..., \pi_{N-1})$	Initial probability Π of HMM is a collection of probabilities. Each element π_j is an initial probability of state j.
A	A $N \times N$ matrix, which is the transition probability matrix of the HMM.
$a_{j,z}$	The element at j^{th} column and z^{th} row of matrix A, indicating the probability transiting from state j to state z
E	A $T \times N$ matrix, which is the emission probability matrix of the HMM.
$e_{t,j}$	The element at t^{th} column and j^{th} row of matrix B, indicating the emission probability of state j at the tth window
$e^{allele}(t, j)$	We denote the factor of emission probability of state j at the tth window computed from the allele-specific reads as $e^{allele}(t, j)$
φ	beta-binomial distribution
settings for exome data	
N	number of individuals in exome data.
T	number of targets of exome data
$d_1, d_2, ..., d_N$	the number of reads for each individual.
$O_{k,t}, 1 \le k \le N, 1 \le t \le T$	read counts for exon t of individual k.
$\mu_{k,t}$	the expected read counts for exon t of individual k.
ϕ_k	the over-dispersion parameter for individual k.
NB	negative binomial distribution.

Table S2: allelic configurations and weights for each state

$w_{j,h}$	j=0	j=1	j=2	j=3	j=4	j=5	j=6+
h=0	1 (-)	0.5 (B)	1(AB)	0.5 (ABB)	0.33 (ABBB)	0.25(ABBBB)	0.17(ABBBBB)
h=1	-	0.5 (A)	-	0.5(AAB)	0.33 (AABB)	0.25(AABBB)	0.17(AABBBB)
h=2	-	-	-	-	0.33(AAAB)	0.25(AAABB)	0.17(AAABBB)
h=3	-	-	-	-	-	0.25 (AAAAB)	0.17(AAAABB)
h=4	-	-	-	-	-	-	0.17(AAAAAB)

Table S3: beta-binomial parameters of allelic configurations of states

$p_{j,h}$	j=0	j=1	j=2	j=3	j=4	j=5	j=6+
h=0	0.99	0.01	0.5	0.33	0.25	0.2	0.17
h=1	-	0.99	-	0.67	0.5	0.4	0.33
h=2	-	-	-	-	0.75	0.6	0.5
h=3	-	-	-	-	-	0.8	0.67
h=4	-	-	-	-	-	-	0.83

Table S4: Methodologies comparisons among WGS methods

(a)	AS-GENSENG	GENSENG	CNVnator	ERDS
**Allele-specific CNV**	Y	N	N	N
**Analyzing exome sequencing data**	Y	N	N	N
**Using read-pair information**	N	N	N	Y for <10kbps deletions
**Bias correction**	• 1-step approach.<br>• Correct for GC content, mappability, and additional noise in the data.	• 1-step approach.<br>• Correct for GC content, mappability, and additional noise in the data.	• 2-step approach.<br>• Correct only for GC content.	• 2-step approach.<br>• Correct only for GC content.
**Use of allele-specific information with read-count**	• Use of beta-binomial distribution to model the allelic imbalance, and combine it into emission probability of HMM.	N	N	• Modeling the total number of heterozygous SNPs in each window, and combining it into the emission probability of HMM.
**Segmentation**	HMM-based approach	HMM-based approach	Mean shift with multiple-bandwidth partitioning	HMM-based approach

(b)	Improvements of AS-GENSENG over GENSENG		
	**Source of Data**	**Call ASCN**	**Call from WES data**
GENSENG	Only total read depth	N	N
AS-GENSENG	Combines total read depth and allele-specific read-depth	Y	Y

Table S5: Methodologies comparisons among WES methods

	AS-GENSENG	XHMM	Conifer	ExomeDepth
**Raw Data**	• Total Read Count (TReC)<br>• Allele Specific Read Count (ASReC)	• Depth of Coverage (DOC)	• Read Count (RC)	• Read Count (RC)
**Normalization**	• Compute the ratio value for each exon target as the TReC of the target to the sum of TReCs of all targets for each sample.<br>• Apply ASReC to find a subset of samples which do not have CNV at the given exon.<br>• Median ratio value of the no CNV samples group as the baseline ratio at the exon.<br>• Multiplies baseline ratio with the sum of TReCs of a sample to compute the expected TReC of the sample at a given exon.	• Apply PCA to remove high-variance noise	• Convert RC to RPKM.<br>• Compute mean RPKM value of all samples as the baseline of a given exon (not accurate when common CNV exists).<br>• Convert RPKM to z-score based on the mean value, and the standard deviation of RPKM values of all samples.	• Build an optimized reference set using a beta-binomial distribution.<br>• Compare the read count with the corresponding read count in the built reference set.
**CNV calling**	• Hidden Markov Model	• Hidden Markov Model	• Segmentation based on fixed threshold based on standard deviation.	• Hidden Markov Model
**Absolute copy number**	• Directly model absolute copy number as the hidden states.	• No absolute copy number calls.	• Required external sources of CNV with absolute copy number.	• No absolute copy number calls.

Table S6: Performance comparison on WGS-read-count-simulation data

Simulation [1]	Average Implanted CNVs[2]		Average Number of True Discoveries[3] (Sensitivity)		Average Reported CNVs[4]		Average Number of False Discoveries[5] (FDR)	
	All	w AS	All	w AS	All	w AS	All	w AS
Deletions	121.7	11.8	112.6 (92%)	11.5 (98%)	114	11.6	0.8 (0.7%)	0.1 (0.7%)
Duplications	78.3	15.5	67.5 (86%)	13.9 (90%)	97.8	13.9	29.9 (30.6%)	0 (0.0%)
Overall CNV	200	27.3	180 (90%)	25.3 (93%)	211.7	25.5	30.7 (14.5%)	0.1 (0.3%)

Note:

1. We simulated 100 datasets for performance assessment. 200 CNVs were simulated in each trial. These 200 CNVs were the ground truth of the trial. The reported CNVs set were compared with the ground truth for each trial. We reported the results stratified by two conditions. The first one is the CNV type (deletions, duplications, and overall CNV). And the second one is the allele specific information (All CNVs and allele specific CNV).
2. On average, we simulated 122 deletions (All), and 12 of them were allele specific CNVs (w AS). On the other hand, we simulated 78 duplications (All), and 15 of them were allele specific CNVs. The mean ASReC of each simulated allele specific CNV (w AS) must be larger than 10.
3. A true discovery was an implanted CNV which with >50% bps overlapping with the called CNVs having the same CNV type. Sensitivity was calculated by dividing the true discoveries by the total number of implanted CNVs (ALL). Sensitivity of CNVs with allele specific reads (w AS) was calculated by dividing the number of true discoveries (w AS) by the total number of the implanted CNVs w AS.
4. A reported CNV is characterized as the chromosome, the starting position of its first covered target, and the ending position of its last covered target. A reported CNV is called with allele specific information (w AS) if its mean allele specific read count across all its covered targets is larger than 10.
5. A false discovery is a reported CNV with < 50% bps overlapping with the implanted CNVs with the same CNV type. FDR was calculated by dividing the number of false discoveries by the total number of the reported CNVs (w ALL). FDR (w AS) was calculated by dividing the number of false discoveries (w ALL) by the total number of the reported CNVs (w AS).

Table S7: Performance comparison on WGS-sequencing-read-simulation data

Simulation [1]	Implanted CNVs[2]		Number of True Discoveries[3] (Sensitivity)		Reported CNVs[4]		Number of False Discoveries[5] (FDR)	
	All	w AS	All	w AS	All	w AS	All	w AS
Deletions	119	15	117 (98%)	15 (100%)	119	15	2 (2%)	0 (0%)
Duplications	81	47	76 (94%)	46 (98%)	88	34	12 (14%)	2 (6%)
Overall CNV	200	62	193 (97%)	61 (98%)	207	49	14 (7%)	2 (4%)

Note:

1. We implanted 200 CNVs into the hypothetical chromosome. These 200 CNVs were the ground truth of the trial. The reported CNVs set were compared with the ground truth for each trial. We reported the results stratified by two conditions. The first one is the CNV type (deletions, duplications, and overall CNV). And the second one is the allele specific information (All CNVs and allele specific CNV).
2. We simulated 119 deletions (All), and 15 of them were allele specific CNVs (w AS). On the other hand, we simulated 81 duplications (All), and 47 of them were allele specific CNVs. The mean ASReC of each simulated allele specific CNV (w AS) must be larger than 10.
3. A true discovery was an implanted CNV which with >50% bps overlapping with the called CNVs having the same CNV type. Sensitivity was calculated by dividing the true discoveries by the total number of implanted CNVs (ALL). Sensitivity of CNVs with allele specific reads (w AS) was calculated by dividing the number of true discoveries (w AS) by the total number of the implanted CNVs w AS.
4. A reported CNV is characterized as the chromosome, the starting position of its first covered target, and the ending position of its last covered target. A reported CNV is called with allele specific information (w AS) if its mean allele specific read count across all its covered targets is larger than 10.
5. A false discovery is a reported CNV with < 50% bps overlapping with the implanted CNVs with the same CNV type. FDR was calculated by dividing the number of false discoveries by the total number of the reported CNVs (w ALL). FDR (w AS) was calculated by dividing the number of false discoveries (w ALL) by the total number of the reported CNVs (w AS).

Table S8: Performance comparison with WGS methods on simulation data

CNV type	Implanted CNVs	Number of True Discoveries[1] (Sensitivity)			Predicted CNVs			Number of False Discoveries[2] (FDR)		
		AS-GENS ENG	ERDS	CNVnator	AS-GENS ENG	ERDS	CNVnator	AS-GENS ENG	ERDS	CNVnator
Deletion	119	117 (98%)	113 (95%)	107 (90%)	119	113	138	2 (2%)	0 (0%)	31 (22%)
Duplication	81	76 (94%)	78 (96%)	75 (93%)	88	156	76	12 (14%)	78 (50%)	1 (1%)
Overall	200	193 (97%)	191 (96%)	189 (91%)	207	269	214	14 (7%)	78 (30%)	32 (15%)

Note:

1. A true discovery is an implanted CNV which with ≥ 50% bps overlapping with the predicted CNVs having the same CNV type. Sensitivity is calculated by dividing the true discoveries by the total number of implanted CNVs.
2. A false discovery is a reported CNV with < 50% bps overlapping with the implanted CNVs with the same CNV type. FDR was calculated by dividing the number of false discoveries by the total number of the reported CNVs.

Table S9: Performance comparison on WGS simulation down-sampled data

Simulation Parameter [1]	Deletion				Duplication			
Coverage	#True Discoveries [2]/#Implanted CNV	Sensitivity	#False Discoveries 2/#Discoveries	FDR	#True Discoveries /#Implanted CNV	Sensitivity	#False Discoveries /#Discoveries	FDR
5x	57/119	48%	0/57	0%	52/81	64%	0/52	0%
10x	90/119	77%	2/92	2%	64/81	79%	3/67	4%
20x	111/119	93%	3/114	3%	74/81	92%	5/79	6%
30x	114/119	96%	3/117	3%	75/81	93%	8/83	10%
40x	117/119	98%	2/119	`2%	76/81	94%	12/88	14%

Note:

. We generated sequencing simulation reads from the same hypothetical genome with coverage varying from 5x to 40x.

. A true discovery is defined as an implanted CNV with ≥ 50% reciprocal overlapped by predicted CNVs. A false discovery is defined as a predicted CNV with <50% reciprocal overlapped by implanted CNVs.

Table S10: Performance comparison on WGS empirical down-sampled data

Parameter [1]	NA12891			NA12892		
Coverage	#True Discoveries[2]/# High Confidence Deletions	Sensitivity	#AS-GENSENG deletion calls / Spanned regions in Mbps	#True Discoveries/# High Confidence Deletions	Sensitivity	#AS-GENSENG deletion calls / Spanned regions in Mbps
5x	768/2200	35%	684/6.8	652/2055	32%	601/5
10x	1018/2200	46%	1403/8.4	880/2055	43%	1360/7.3
20x	1178/2200	54%	1817/13.7	1044/2055	51%	1897/11.3
25x	-	-	-	1079/2055	53%	2347/24.3
30x	1222/2200	56%	2302/20.4	-	-	-

Note:

1. The coverage of NA12891 sequencing data provided by 1000GP is 30x. We down-sampled sequencing reads from the 1000GP sequencing data with the target coverage from 5x, 10x, to 20x. The coverage of NA12892 sequencing data provided by 1000GP is 25x. We down-sampled sequencing reads from the 1000GP sequencing data with the target coverage from 5x, 10x, to 20x.
2. A true discovery is defined as a high-confidence CNV with ≥ 50% reciprocal overlapped by predicted CNVs. We used the volume of calls (number, spanned bps) as a surrogate measurement of false discoveries.

Table S11: Performance comparison on WES-simulation data

Simulation Parameter [1]	Average Implanted CNVs [2]		Average Number of True Discoveries [3] (Sensitivity)				Average Predicted CNVs [4]				Average Number of False Discoveries [5] (FDR)			
CNV frequency	Deletions (w AS)	Duplications (w AS)	Deletions		Duplications		Deletions		Duplications		Deletions		Duplications	
			All	w AS	All	w AS	All	w AS	All	w AS	All	w AS	All	w AS
0.01	10 (2.3)	10 (8.1)	8.9 (89%)	2.1 (95%)	9.1 (91%)	7.5 (93%)	9.0	2.0	9.5	7.5	0.01 (0%)	0 (0%)	0.0 (0%)	0 (0%)
0.05	50 (10.4)	50 (40.3)	40.3 (81%)	9.5 (91%)	45.6 (91%)	37.7 (93%)	44.0	9.0	47.2	36.6	0.03 (0%)	0.01 (0%)	0.04 (0%)	0.01 (0%)
0.1	20 (3.7)	20 (15.6)	16.4 (82%)	3.4 (91%)	17.9 (89%)	14.6 (93%)	17.6	3.2	18.7	14.2	0.01 (0%)	0 (0%)	0.01 (0%)	0 (0%)
0.2	40 (8.5)	40 (30.6)	34 (85%)	7.8 (92%)	35.9 (90%)	28.1 (92%)	36.0	7.5	38.7	28.3	0.44 (1.3%)	0 (0%)	0.03 (0%)	0.01 (0%)

Note:

1. The CNV frequency is the only parameter used in the simulation. It determines the number of individuals having the CNV in the population. We obtained total read counts and real read counts from chr11 of individual HG00264 as the template. We then sampled *N* individuals from a total of 100 individuals. *N* is determined by the CNV frequency. Only the targets of the selected individuals will be affected. If the CNV is a duplication, the read count would be increased, and if the CNV is a deletion, the read count would be decreased accordingly.
2. When CNV frequency equals 0.01 (and 0.05), we simulated 1000 deletions, and 1000 duplications. After counting for CNV frequency, each individual will have around 10 (and 50) deletions and 10 (and 50) duplications on average. When CNV frequency equals 0.1 (and 0.2), we simulated 200 deletions and 200 duplications. After counting for CNV frequency, each individual has around 20 deletions (and 40 deletions), and 20 duplications (and 40 duplications). We also simulated allele specific read counts (AS) for each target. It is a random number sampled from a binomial distribution (n=read count of the target, p=0.05.) The CNV is called with allele specific information (w AS) if the mean value of AS of all targets in the CNV is larger than 10.
3. A true discovery was an implanted CNV which with >=1bps overlapping with the called CNVs having the same CNV type. Sensitivity was calculated by dividing the true discoveries by the total number of implanted CNVs (ALL). Sensitivity of CNVs with allele specific reads (w AS) was calculated by dividing the number of true discoveries (w AS) by the total number of the implanted CNVs w AS.
4. A predicted CNV is characterized as the chromosome, the starting position of its first covered target, and the ending position of its last covered target. A reported CNV is called with allele specific information (w AS) if its mean allele specific read count across all its covered targets is larger than 10.
5. A false discovery is a predicted CNV with no bps overlapping with the implanted CNVs with the same CNV type. FDR was calculated by dividing the number of false discoveries by the total number of the reported CNVs (w ALL). FDR (w AS) was calculated by dividing the number of false discoveries (w ALL) by the total number of the reported CNVs (w AS).

Table S12: Performance comparison with WES methods on WES-simulation data

Simulation Parameter [1]	Average Implanted CNVs[2]	Average Number of True Discoveries[3] (Sensitivity)				Average Predicted CNVs[4]				Average Number of False Discoveries[5] (FDR)			
CNV frequency		AS-GENSENG	Exome Depth[6]	Conifer[7]	XHMM[8]	AS-GENSENG	Exome Depth[6]	Conifer[7]	XHMM[8]	AS-GENSENG	Exome Depth[6]	Conifer[7]	XHMM[8]
1%	20	17.91 (89.6%)	16.48 (82.4%)	10.66 (53.3%)	17.53 (87.7%)	18.54	16.41	10.5	17.63	0.01 (0%)	0.01 (0%)	0.04 (0.4%)	0.34 (1.9%)
5%	100	85.86 (85.9%)	35.13 (35.13)	29.63 (29.63%)	5.99 (5.99%)	91.32	33.28	27.95	5.78	0.07 (0%)	0.00 (0%)	0.02 (0%)	0.07 (1.2%)
10%	40	34.33 (85.8%)	24.36 (60.9%)	4.99 (12.5%)	0.82 (2.1%)	36.28	24.36	5.49	0.83	0.02 (0%)	0.00 (0%)	0.08 (1.5%)	0.01 (1.2%)
20%	80	69.89 (87.4%)	27.36 (34.2%)	1.04 (1.3%)	0.12 (0.2%)	73.67	27.38	1.57	0.13	0.47 (1%)	0.02 (0%)	0.37 (23.5%)	0.01 (7.7%)

Note:

1. The CNV frequency is the only parameter used in the simulation. It determines the number of individuals having the CNV in the population. We obtained total read counts and real read counts from chr11 of individual HG00264 as the template. For each CNV, we first selected 1-3 consecutive targets. We then sampled *N* individuals from a total of 100 individuals. *N* is determined by the CNV frequency. Only the targets of the selected individuals will be affected. If the CNV is a duplication, the read count would be increased, and if the CNV is a deletion, the read count would be decreased accordingly.
2. When CNV frequency equals 1% and 5%, we simulated 1000 deletions, and 1000 duplications. After counting for CNV frequency, each individual will have around 10 (and 50) deletions and 10 (and 50) duplications on average. When CNV frequency equals 10% and 20%, we simulated 200 deletions and 200 duplications. After counting for CNV frequency, each individual has around 20 deletions (and 40 deletions), and 20 duplications (and 40 duplications).
3. A true discovery is an implanted CNV which with ≥ 1 bps overlapping with the called CNVs having the same CNV type. Sensitivity is calculated by dividing the true discoveries by the total number of implanted CNVs.
4. A reported CNV is characterized as the chromosome, the starting position of its first covered target, and the ending position of its last covered target
5. A false discovery is a reported CNV without any bps overlapping with the implanted CNVs with the same CNV type. FDR was calculated by dividing the number of false discoveries by the total number of the reported CNVs.
6. ExomeDepth chooses the reference samples from all samples assuming no CNV in the reference samples. Thus it suggests to detect rare CNV as the assumption may not hold in common CNV case. In this study, we observed that the sensitivity drops when the CNV frequency increases. In contrast, AS-GENSENG applied AS information to identify the copy number two reference as the baseline immune to the common CNV. As a result, we observed that the performance of AS-GENSENG being consistent among difference CNV frequencies.
7. Conifer applies SVD to remove noise in WES data. The number of removed components reflects its assumption on the variances contributed by system bias and by CNV. It removes more components for rare CNV as much of the variance is contributed by system bias. In contrast, it removes less number of components for common CNV as more variance is contributed by CNV in this case. However, the common CNV is unknown until the CNVs are detected. In this study, we observed the sensitivity drops when the CNV frequency increases. In contrast, AS-GENSENG applied AS information to identify the copy number two reference as the baseline immune to the common CNV. As a result, we observed that the performance of AS-GENSENG being consistent among difference CNV frequencies.
8. XHMM applies PCA to normalize the WES data. In the next step it applies a HMM to infer CNV. Its focus is rare CNV.
